# Abnormal Amyloid-β Duration, Tau, and Neurodegeneration in Cranial Images

**DOI:** 10.1001/jamanetworkopen.2025.31093

**Published:** 2025-09-09

**Authors:** Henry Gilreath Stephenson, Rebecca Langhough, Erin Jonaitis, Lianlian Du, Carol Van Hulle, Nathaniel A. Chin, Ozioma C. Okonkwo, Cynthia M. Carlsson, Sanjay Asthana, Bradley T. Christian, Sterling C. Johnson, Barbara B. Bendlin, Tobey J. Betthauser

**Affiliations:** 1 School of Medicine and Public Health, University of Wisconsin–Madison, Madison; 2 Neuroscience Training Program, University of Wisconsin–Madison, Madison; 3 Wisconsin Alzheimer’s Disease Research Center, University of Wisconsin–Madison, Madison; 4 Wisconsin Alzheimer’s Institute, University of Wisconsin–Madison, Madison; 5 Department of Neurological Sciences, Rush University Medical Center, Chicago, Illinois; 6 Veterans Administration William S. Middleton Memorial Veterans Hospital, Madison, Wisconsin; 7 Department of Medical Physics, University of Wisconsin–Madison, Madison; 8 Department of Psychiatry, University of Wisconsin–Madison, Madison

## Abstract

**Importance:**

It is unclear whether the duration of amyloid-β (Aβ) pathology is associated with neurodegeneration and whether this depends on the presence of tau.

**Objective:**

To examine the association of longitudinal atrophy with Aβ positron emission tomography (PET)–positivity (Aβ+) and the estimated duration of Aβ+ (Aβ+ duration), controlling for tau-positivity.

**Design, Setting, and Participants:**

Data for this longitudinal cohort study were drawn from the Wisconsin Registry for Alzheimer Prevention and the Wisconsin Alzheimer Disease Research Center Clinical Core Study. Participants who had 2 or more magnetic resonance imaging (MRI) visits, 1 Pittsburgh compound B (PiB) PET visit, and 1 MK-6240 PET visit with other covariates available were included. A replication analysis was conducted using data from the OASIS-3 dataset. All data were collected between June 1, 2009, and January 22, 2025. Follow-up times ranged from 1.0 to 13.0 years (median [IQR], 8.8 [5.9-10.6] years).

**Exposures:**

Sampled iterative local approximation (SILA)-estimated PiB PET uptake at baseline MRI was used to estimate Aβ+ and its duration at baseline MRI. MK-6240 PET uptake in the entorhinal cortex was used to create tau-positive and tau-negative groups using a threshold of 1.27 standardized uptake value ratio.

**Main Outcomes and Measures:**

SILA-based modeling of PiB PET data was used to obtain estimated Aβ+ duration at baseline MRI age. Linear mixed-effects models tested differences in atrophy between Aβ+ vs Aβ− individuals and with Aβ+ duration, controlling for individuals who were tau-positive (measured via MK-6240 tau PET) at their most recent PET scan within their MRI visits. *Z*-scored volumes in temporo-parietal regions of interest associated with AD dementia were assessed via a robust normative approach.

**Results:**

A total of 95 Aβ+ (median [IQR] age, 66.4 [61.1-70.4] years; 59 female [62.11%]) and 275 Aβ− (median [IQR] age, 60.2 [55.7-64.6] years; 183 [66.55%] female) individuals were included. Aβ+ (partial η^2^, 0.015-0.043) and tau-positivity (partial η^2^, 0.018-0.100) were independently associated with neurodegeneration, with generally small effect sizes. Aβ+ duration was associated with atrophy in a greater number of regions of interest than Aβ+ status alone with somewhat larger effect sizes (partial η^2^, 0.013-0.056). Results were mostly similar in the OASIS-3 dataset.

**Conclusions and Relevance:**

In this longitudinal cohort study, subtle neurodegeneration was observed soon after the onset of Aβ pathology. These results may be consistent with Aβ pathology as a pathological state.

## Key Points

**Question:**

Is regional neurodegeneration associated with the timeline of amyloid-β (Aβ) accumulation?

**Findings:**

In this cohort study that included 370 predominantly cognitively unimpaired individuals with Aβ and tau positron emission tomography alongside longitudinal magnetic resonance imaging data, subtle, accelerated brain region atrophy was observed with Aβ-positivity and Aβ-positivity duration, controlling for individuals who later became tau-positive.

**Meaning:**

These findings suggest that neurodegeneration may begin soon after the onset of abnormal Aβ pathology.

## Teaser

This cohort study investigates whether the presence or duration of amyloid-β pathology is associated with neurodegeneration and whether this association depends on the presence of tau in a sample of adults who were predominately cognitively unimpaired at baseline imaging.

## Introduction

There is considerable controversy about whether amyloid-β (Aβ) plaques are benign or pathological in nature. Recently proposed guidelines from the Alzheimer Association Working Group consider anyone who harbors abnormal core 1 biomarkers, including abnormal levels of Aβ positron emission tomography (PET) uptake, as having Alzheimer disease (AD).^1,2^ In contrast, International Working Group guidelines^3^ state that a diagnosis of AD cannot be made without symptoms, arguing that abnormal core 1 biomarkers are not associated with a markedly higher risk of symptomatic AD compared with normal levels. Similarly, studies investigating the association between Aβ and longitudinal atrophy have shown limited associations between the 2, particularly in the absence of tau, suggesting that Aβ-positivity (Aβ+) on its own is a largely benign finding prior to the development of tau pathology.^4,5^ However, other studies have found accelerated atrophy among individuals with emerging Aβ pathology for whom little tau would be expected.^6,7^ Furthermore, studies to date have not directly investigated longitudinal rates of atrophy in the context of how long Aβ+ individuals have harbored Aβ, precluding strong conclusions about how soon after onset of Aβ pathology regional atrophy can be detected and the extent to which this depends on tau.

The purpose of this study was to investigate whether Aβ+ and the duration of Aβ+ (Aβ+ duration) are associated with regional neurodegeneration in a predominantly cognitively unimpaired (CU) sample with generally short Aβ+ duration. It was hypothesized that Aβ+ in this sample with early pathology would be associated with an accelerated pattern of AD-like atrophy that worsened with longer durations of Aβ+, controlling for individuals who became tau-positive within their observation period.

## Methods

### Study Design and Sample Selection

Descriptions of participant selection, analyses, and the results obtained follow the Strengthening the Reporting of Observational Studies in Epidemiology (STROBE) reporting guideline for cohort studies. eFigure 1 in Supplement 1 provides a flowchart of the sample selection. Data were drawn from the Wisconsin Registry of Alzheimer Prevention (WRAP) and the Wisconsin Alzheimer Disease Research Center Clinical Core Study (ADRC) cohorts, 2 longitudinal cohort studies at the University of Wisconsin–Madison. All participants or their caregivers provided informed written consent according to the Declaration of Helsinki.^8^ Study procedures were approved by the University of Wisconsin–Madison Institutional Review Board. Data included in this analysis were collected between June 1, 2009, and January 22, 2025. Race and ethnicity were assessed to more fully characterize the cohort's demographic makeup. Self-reported data were used to assign individuals to White (non-Hispanic) or other groups (race and ethnicity other than non-Hispanic White) based on each participant's primary reported ethnicity.

Participants were included if they had (1) at least 2 T1-weighted volumetric imaging visits, (2) 1 carbon 11–labeled Pittsburgh compound B positron emission tomography ([^11^C] PiB PET), (3) 1 fluorine-18-MK-6240 PET^9^ imaging visit within their longitudinal magnetic resonance imaging (MRI) period (and up to 2 years after their final MRI), (3) a cognitive status or diagnosis within 2 years of their baseline MRI, and (4) apolipoprotein E *(APOE)* genotyping. Participants with missing data were not included. Cognitive status or diagnosis was determined via multidisciplinary consensus review^10,11^ according to the National Institute on Aging –Alzheimer Association guidelines. Sampled iterative local approximation (SILA) modeling was used to determine Aβ+ duration at baseline MRI scan using each individual’s latest [^11^C] PiB-PET scan (see PET Imaging section for details). Individuals were considered tau-positive or tau-negative based on a volume-weighted standardized uptake value ratio (SUVR) of 1.27 in the entorhinal cortex at their most recent PET visit within their longitudinal MRI observation period because ^18^F-MK-6240 was not available at baseline MRI for most individuals due to the relative novelty of this tracer. This approach resulted in a sample of 95 Aβ+ individuals (11 who were cognitively impaired [CI], including both mild cognitive impairment [MCI] and dementia, and 49 who were tau-positive), and 275 Aβ− cognitively unimpaired (CU) individuals (10 CI, 22 tau-positive), with 1519 observations total and a median (IQR) follow-up of 8.8 (5.9-10.6) years.

A replication analysis was carried out using data from the Open Access Series of Imaging Studies 3 (OASIS-3) dataset, which was filtered according to the same aforementioned inclusion criteria. This resulted in a sample of 32 individuals with Aβ+ (5 CI, including both MCI and dementia, 16 tau-positive), and 159 Aβ− CU individuals (4 CI, 15 tau-positive), with 673 observations total and a median (IQR) follow-up of 6.1 (3.5-9.0) years. See Replication Analysis section for details.

### Longitudinal Volumetric MRI

Volumetric data were collected using T1-weighted imaging sequences acquired on 1 of 2 3-Tesla Discovery MR750 (GE Healthcare) MRI scanners with either an 8-channel Excite (GE Healthcare) or 32-channel (Nova Medical) head coil. A longitudinal segmentation pipeline was used with SPM12 software to initialize processing of individual volumes using a within-person longitudinal template to increase reliability and sensitivity to longitudinal changes. The eMethods 1 in Supplement 1 provides longitudinal processing details, and eTables 1 and 2 in Supplement 1 give sequencing details and scanner and head coil numbers.

### Region of Interest Selection

**Figure 1.**
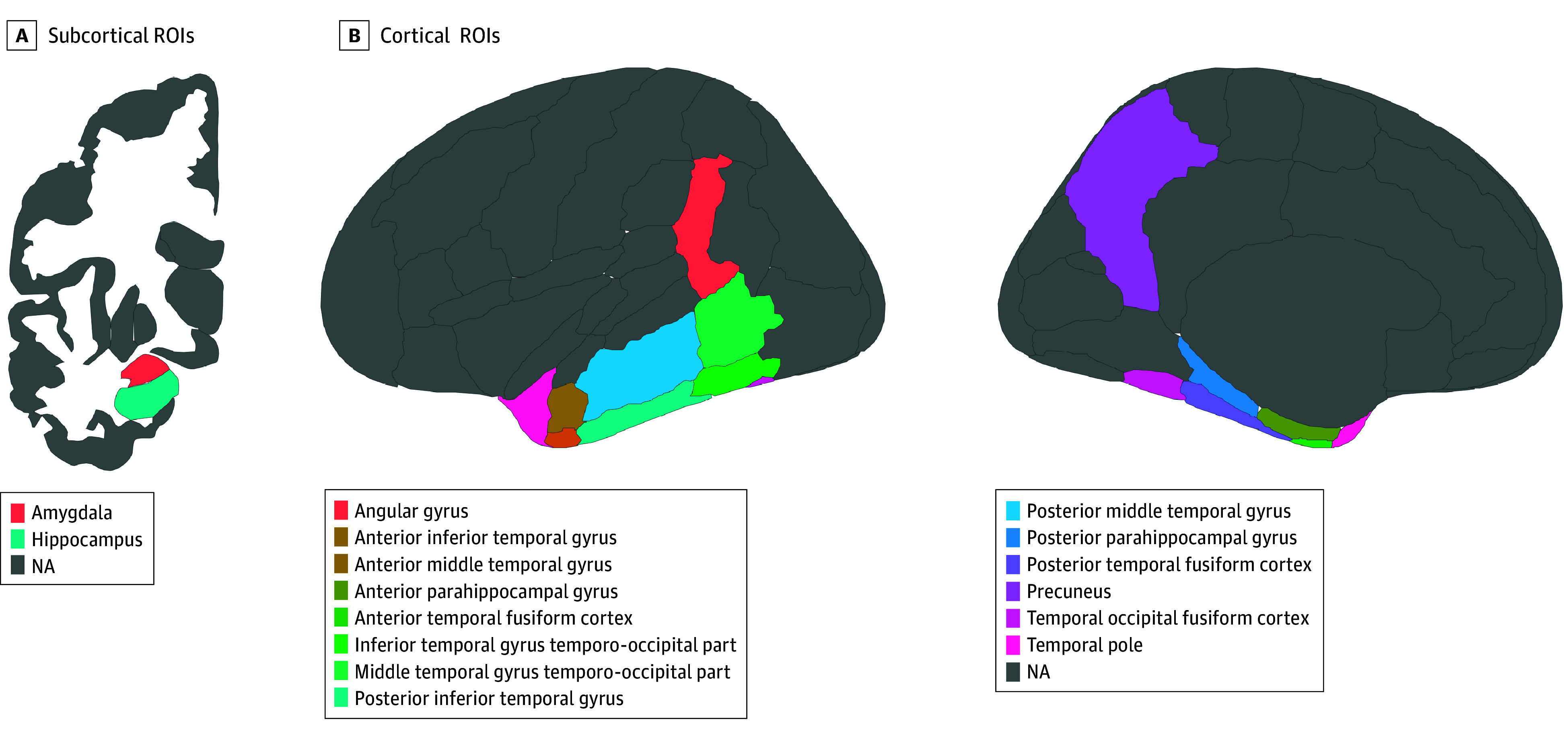
Harvard-Oxford Atlas Subcortical and Cortical Regions of Interest (ROIs) Selected for Analysis Based on Sensitivity to Alzheimer Disease–Associated Neurodegeneration NA indicates not applicable.

Temporo-parietal regions of interest (ROIs) were selected based on prior work from members of our group and previous research on ROIs that delineate AD dementia cases from controls.^12,13^ These ROIs included the amygdala, hippocampus, parahippocampal gyri (anterior and posterior), temporal fusiform cortices (anterior, posterior, and temporal-occipital parts), temporal pole, inferior and middle temporal gyri (anterior, posterior, and temporal-occipital parts), angular gyrus, and precuneus (Figure 1). Left and right volumes were averaged to reduce the number of comparisons.

### PET Imaging

All participants underwent ^11^C-PiB PET and ^18^F-MK-6240 PET imaging at the University of Wisconsin–Madison Waisman Brain Imaging Lab. Details regarding radiopharmaceutical production, acquisition protocols, and quantification of ^11^C-PiB and ^18^F-MK-6240 images have been described previously.^14,15,16^ Methods did not differ across WRAP and ADRC cohorts. Aβ burden was assessed as a global average PiB distribution volume ratio (DVR) across 8 bilateral cortical regions using cerebellum gray matter as a reference region.^17^ The SILA algorithm was used to obtain estimated Aβ+ onset age for each participant as previously described.^18^ Briefly, SILA can be used to estimate the time course of Aβ PET pathology by modeling longitudinal change rates at each level of tracer uptake. By estimating how much pathology is predicted to change over time across the range of uptake values, the time course of pathology can be reconstructed relative to a given threshold for positivity and used to determine whether and for how long an individual has been Aβ+. SILA has previously been shown to be highly accurate for the forward and backward prediction of Aβ+ and Aβ− across multiple cohorts.^18^ The SILA model used in the present analyses was derived using a larger WRAP and ADRC sample with longitudinal PET data (eTable 3 and eFigure 2 in Supplement 1). Each participant’s most recent PiB DVR was used to estimate the age of Aβ+ onset, which was the age at which each participant was estimated to reach a DVR of 1.16 or higher or 17.1 centiloids.^19^ Individuals with an estimated baseline MRI Aβ+ duration of at least 0 were considered Aβ+ while those with an estimated baseline MRI Aβ+ duration less than 0 were considered Aβ−. Some Aβ− individuals were projected to convert to Aβ+ within their longitudinal MRI period. These individuals were excluded unless they had longitudinal MRIs after the estimated Aβ+ onset, in which case they were considered Aβ+, and only these scans were used. This procedure was used in order to remove Aβ+ converters from the Aβ− sample and to enhance statistical power to detect changes associated with early Aβ+ (N = 33; median estimated Aβ+ conversion 3.2 [IQR, 1.7-5.0] years after first MRI). Entorhinal tau was assessed using a volume-weighted average of left and right anterior parahippocampal gyri SUVR, with the inferior cerebellar gray matter as a reference region. Tau-positivity was defined as an SUVR of 1.27 or higher.^20^

### Robust Normative *z* Scoring

Similar to previous work from members of our group,^12,21,22,23,24^ a robust normative approach was used to identify an Aβ−, tau-negative, and CU sample free from abnormal atrophy and *z* score volumes according to this group. This was done to regress out projected effects of age and other covariates from volume measures and to rescale longitudinal trajectories to represent abnormality relative to this control group. See eMethods 2 in Supplement 1 for further details and explanation of the *z* scores.

### Longitudinal Atrophy, Aβ+, and Aβ+ Duration

To test whether Aβ+ was associated with accelerated atrophy, models projecting *z*-scored volumes were estimated with Aβ+ vs Aβ− at baseline MRI, time, and an interaction between baseline MRI Aβ+ vs Aβ− and time. To ensure that the results of Aβ+ or Aβ− were not associated with the higher prevalence of impairment in Aβ+ vs Aβ− individuals, diagnosis (CI vs CU) and an interaction between diagnosis and time were added as covariates. To assess whether the results were associated with tau, tau-positive vs tau-negative, and an interaction between tau-positive vs tau-negative and time were added as well. To assess the association between atrophy and progressive increases in Aβ+ duration, models were repeated substituting Aβ+ duration (0 in Aβ− individuals, 0+ in Aβ+ individuals) for Aβ+ and Aβ−.

### Sensitivity Analyses

To further ensure that the results were not associated with CI individuals in the Aβ+ group and to assess the results without possible associations with non-AD pathology in the Aβ− CI group, all analyses were repeated with CU individuals only. To ensure that results were not associated with differences in atrophy at the level of the intercept (ie, by individuals who started at low volume), analyses were repeated with baseline volumes subtracted from each individual’s longitudinal volumes.

### Replication Analyses

To replicate these findings, additional data were drawn from the OASIS-3 dataset. See eFigure 3 in Supplement 1 for a flow diagram of sample selection. See LaMontagne et al^25^ for details on OASIS-3 imaging methods and FreeSurfer-based processing for ^11^C-PiB PET and ^18^F-AV-45 (florbetapir) Aβ PET, and ^18^F-AV-1451 (flortaucipir) tau PET. eMethods 1 and 3 in Supplement 1 provide details on MRI processing and tau-positive cut points. eTable 4 and eFigure 4 in Supplement 1 give details on SILA modeling. eMethods 1 in Supplement 1 provides details on MRI processing. The *z* scoring and statistical analyses were conducted as described in the Statistical Analysis section. See eMethods 1 and 3 in Supplement 1 for details. Sensitivity analyses examining CU individuals only were not conducted due to the lower sample size in the OASIS-3 dataset.

### Statistical Analysis

Data analysis and visualization were performed in R version 4.3.1 (R Project for Statistical Computing).^26^ Mean (SD) was used for normally distributed variables while median (IQR) was used for non-normally distributed variables. Visualization was conducted using the ggplot and ggseg packages.^27,28^ Analyses used linear mixed-effects models, implemented using the lmerTest package.^29,30^ Following *z* scoring, models were adjusted for *APOE4* status (carrier vs noncarrier) and included random intercepts for participant and random slopes for time (years since baseline). Results were considered significant at a 2-sided *P* < .05 (false discovery rate–corrected^31^). Effect sizes were assessed using partial η^2^ estimates from the effectsize package,^32^ benchmarked according to the recommendations of Cohen (small, partial η^2^ ≥0.02 to <0.13; medium, ≥0.13 to <0.26; and large, ≥0.26).^33^

## Results

### Sample Characteristics

A total of 95 Aβ+ individuals (median [IQR] age, 66.4 [61.1-70.4] years; 59 female [62.11%]; 36 [37.89%] male) and 275 Aβ− individuals (median [IQR] age, 60.2 [55.7-64.6] years; 183 [66.55%] female; 92 [33.45%] male) were included in this analysis. In the Aβ+ sample, 87 (91.58%) were non-Hispanic White and 8 (8.42%) belonged to other racial and ethnic groups, and in the Aβ− sample, 251 (91.27%) were non-Hispanic White and 24 (8.73%) belonged to other racial and ethnic groups. Follow-up times ranged from 1.0 to 13.0 years (median [IQR], 8.8 [5.9-10.6] years). The Aβ+ sample was predominantly CU (84 of 95) with only 2 individuals diagnosed with dementia, 8 with MCI, and 1 diagnosed as other. The Aβ− sample was mostly CU (265 of 275), with only 1 diagnosed with dementia, 6 with MCI, and 3 as other. The groups differed significantly in terms of *APOE4* status, CI, number of tau-positive, age at baseline MRI, length of longitudinal follow-up, and tau burden at baseline such that the Aβ+ group had a higher proportion of *APOE4* carriers, CI, and tau-positivity, was older, had shorter longitudinal follow-up, and had higher entorhinal SUVR (Table).

**Table.** Sample Characteristics

Characteristic	Whole sample, median (IQR) (N = 370)	Aβ− sample, median (IQR) (n = 275)	Aβ+ sample, median (IQR) (n = 95)	*P* value^a^
Sex, No. (%)				
Female	242 (65.41)	183 (66.54)	59 (62.11)	.51
Male	128 (34.59)	92 (33.45)	36 (37.89)	
Race and ethnicity, No. (%)^b^				
Non-Hispanic White	338 (91.35)	251 (91.27)	87 (91.58)	.99
Other^c^	32 (8.65)	24 (8.73)	8 (8.42)	
*APOE4* positive, No. (%)	152 (41.08)	87 (31.64)	65 (68.42)	<.001
Cognitively impaired, No. (%)	21 (5.68)	10 (3.64)	11 (11.58)	.009
Tau-positive, No. (%)	71 (19.19)	22 (8.00)	49 (51.58)	<.001
Baseline Aβ+ duration, y	−13.66 (1.24 to 7.51)	−17.87 (−29.05 to −11.55)	3.57 (1.24 to 7.51)	NA
PiB DVR used for SILA	1.10 (1.41 to 1.75)	1.07 (1.03 to 1.12)	1.56 (1.41 to 1.75)	NA
Age at baseline MRI, mean (SD), y	61.8 (7.34)	60.3 (7.0)	66.2 (6.7)	<.001
Length of follow-up, y	8.8 (5.9 to 10.6)	9.2 (6.6 to 10.9)	7.1 (3.7 to 9.2)	<.001
No. of visits	4 (3 to 5)	4 (3 to 5)	4 (2 to 5)	.04
Entorhinal MK-6240 SUVR	1.04 (0.95 to 1.20)	1 (0.92 to 1.1)	1.28 (1.08 to 1.95)	<.001
Baseline hippocampal volume, mL	3.67 (3.39 to 3.97)	3.71 (3.42 to 4.00)	3.59 (3.23 to 3.87)	.68
Aβ PET age difference at baseline MRI, y	8.89 (6.07 to 11.08)	9.54 (6.71 to 11.24)	7.30 (4.13 to 9.61)	<.001
Tau PET age difference at baseline MRI, y	8.24 (5.81 to 10.25)	8.57 (6.16 to 10.53)	7.58 (3.20 to 9.29)	<.001
Aβ PET age difference at final MRI, y	0.01 (−0.02 to 1.25)	0.01 (−0.01 to 1.37)	0 (−0.04 to 0.85)	.28
Tau PET age difference at final MRI, y	0 (−0.18 to 0.11)	0 (−0.57 to 0.11)	0 (−0.01 to 0.10)	.10
Diagnosis or MRI age difference, y	1.51 (0 to 1.82)	1.53 (0.04 to 1.83)	1.37 (0 to 1.79)	.22

Abbreviations: Aβ+, amyloid-β–positive; DVR, distribution volume ratio; MRI, magnetic resonance imaging; NA, not applicable; PET, positron emission tomography; PiB, Pittsburgh compound B; SILA, sampled iterative local approximation; SUVR, standardized uptake value ratio.

^a^ *P* values taken from unpaired samples *t* tests or χ^2^ tests comparing Aβ+ and Aβ− groups, as appropriate for each variable and distribution. Age, sex, and intracranial volume were added as covariates when comparing hippocampal volumes in these models. Age and sex were added as covariates when comparing entorhinal MK-6240 SUVR values.

^b^ White vs other race and ethnicity was assigned based on each participant’s self-reported primary race.

^c^ The other race and ethnicity category included individuals who reported race and ethnicity other than non-Hispanic White.

### Robust Normative *z* Scoring

After initial *z* scoring of CU individuals in the Aβ− control group, 32 of 244 had abnormally low volume (indicator of the presence of neurodegeneration [N+]) in at least 1 ROI and were removed from the final *z* scoring sample. See eTables 5 and 6 in Supplement 1 for regional model outputs. eFigure 5 in Supplement 1 shows counts of N+ by ROI in the initial *z* scoring sample. eFigures 6 and 7 in Supplement 1 provide uncorrected volumes and *z*-scored volumes by ROI in the final *z* scoring sample.

### Longitudinal Atrophy and Aβ+

**Figure 2.**
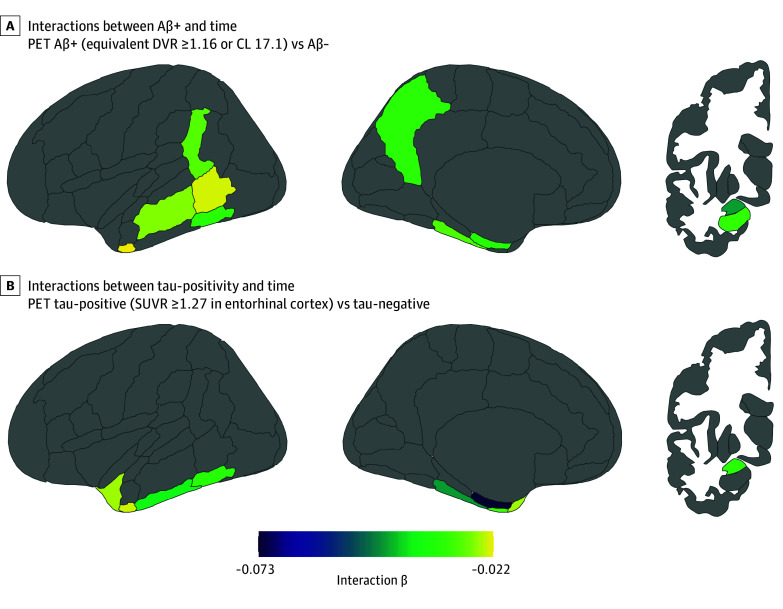
Regional Interactions of Amyloid-Beta–Positivity (Aβ+) and Tau-Positivity With Time A, β Estimates of interactions between Aβ+ and time that survived false discovery rate correction (Aβ+ n = 95). B, β Estimates of interactions between tau-positivity and time that survived false discovery rate correction (tau-positive n = 71). CL indicates centiloids; DVR, distribution volume ratio; PET, positron emission tomography; and SUVR, standardized uptake value ratio.

**Figure 3.**
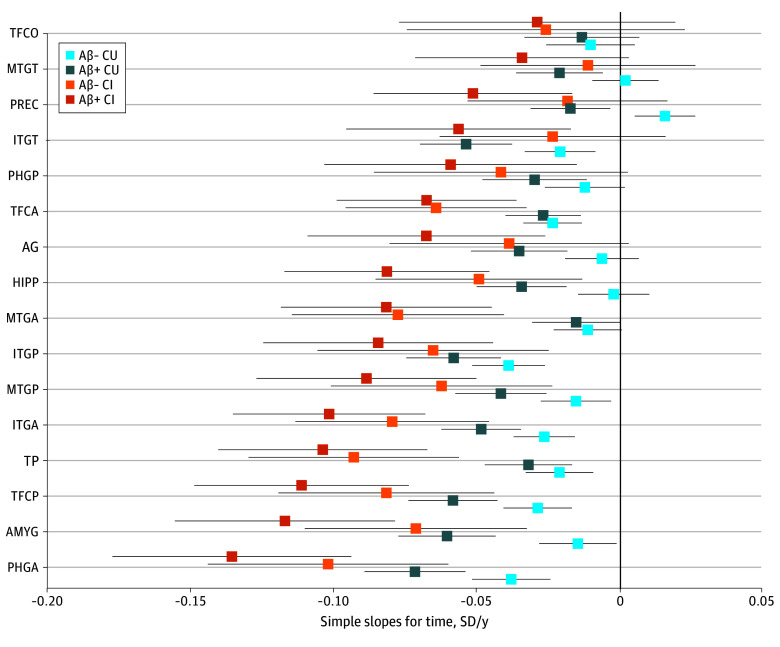
Regional Simple Slopes for Change in *z* Scores Among Individuals Amyloid-β–Negative (Aβ−) and Cognitively Unimpaired (CU), Aβ-Positive (Aβ+) and CU, Aβ− and Cognitively Impaired (CI), and Aβ+ and CI Points show simple slopes while lines show the lower and upper bounds of 95% CIs. AG represents angular gyrus, AMGY, amygdala; HIPP, hippocampus; IGTA, anterior inferior temporal gyrus; ITGP, posterior inferior temporal gyrus; ITGT, inferior temporal gyrus temporo-occipital part; PHGA, anterior parahippocampal gyrus; PHGP, posterior parahippocampal gyrus; PREC, precuneus; MTGA, anterior middle temporal gyrus; MGTG, middle temporal gyrus temporo-occipital part; MTGP, posterior middle temporal gyrus; TFCA, anterior temporal fusiform cortex; TFCP, posterior temporal fusiform cortex; TFCO, temporal occipital fusiform cortex; and TP, temporal pole.

Aβ+ was associated with accelerated atrophy as evidenced by significant interactions between Aβ+ vs Aβ− and time in 10 of 16 ROIs. Effect sizes in these ROIs were small, with a few negligible effects (partial η^2^, 0.015-0.043). The top 4 ROIs by effect size were the amygdala (slope, −0.046 [95% CI, −0.068 to −0.023]; partial η^2^, 0.043), precuneus (slope, −0.033 [95% CI, −0.051 to −0.015]; partial η^2^, 0.034), temporal-occipital inferior temporal gyrus (slope, −0.033 [95% CI, −0.054 to −0.012]; partial η^2^, 0.029), and hippocampus (slope, −0.032 [95% CI, −0.052 to −0.012]; partial η^2^, 0.025). There was a significant interaction between tau positivity vs negativity and time in 8 of 16 ROIs, with generally small effect sizes (partial η^2^, 0.018-0.100). The largest effect size was in the anterior parahippocampal gyrus (slope, −0.073 [95% CI, −0.056 to −0.011]; partial η^2^, 0.100) (Figure 2). CI individuals showed significantly accelerated atrophy in 9 of 16 ROIs relative to CU individuals, with generally small effect sizes (partial η^2^, 0.013-0.034). Simple slopes analyses for time showed that atrophy increased in a stepwise fashion from Aβ− CU to Aβ+ CU, AΒ− CI, and Aβ+ CI (Figure 3). eFigures 8 and 9 in Supplement 1 provide full depictions of Aβ+ and tau-positive results with simple slopes and plots showing projected trajectories over time. eTable 7 in Supplement 1 gives the slopes, confidence intervals, uncorrected and false discovery rate–corrected *P* values, and effect sizes for all interaction terms. There was no main effect of Aβ+ or tau-positivity in any ROI, indicating that neither was associated with differences in baseline volume; however, diagnosis was associated with lower baseline volume in a few ROIs (eTable 8 in Supplement 1 gives full results of the main effects).

### Longitudinal Atrophy and Aβ+ Duration

**Figure 4.**
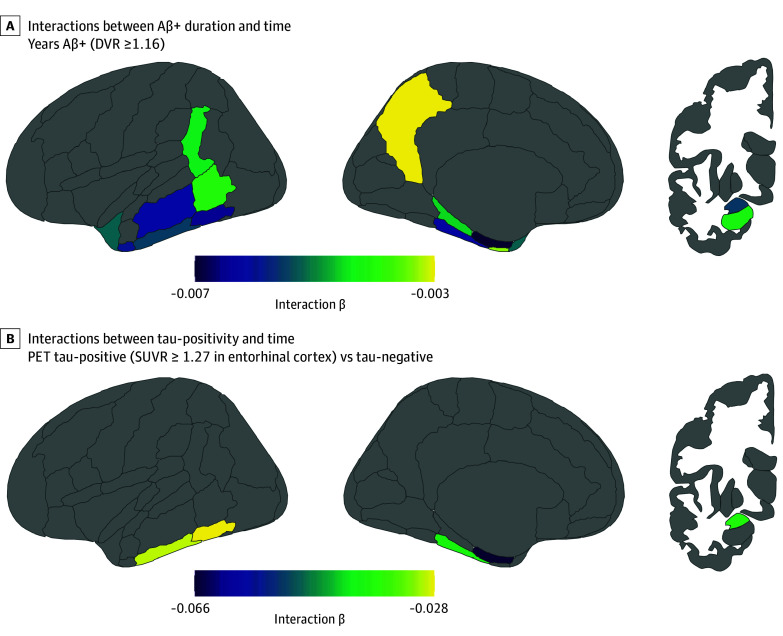
Regional Interactions Between Amyloid-Beta–Positivity (Aβ+) Duration and Tau-Positivity With Time A, β estimates of interactions between Aβ+ duration and time that survived false discovery rate correction (Aβ+ n = 95). B, β estimates of interactions between tau-positivity and time that survived false discovery rate correction (tau-positive n = 71). DVR indicates distribution volume ratio; PET, positron emission tomography; and SUVR, standardized uptake value ratio.

Aβ+ duration was a median (IQR) of 3.6 (1.2-7.5) years among individuals who showed Aβ+ at baseline MRI and was associated with accelerated atrophy in 14 of 16 ROIs. Effect sizes were generally small (partial η^2^, 0.013-0.056). The top 4 ROIs by effect size were the anterior inferior temporal gyrus (slope, −0.006 [95% CI, −0.008 to −0.003]; partial η^2^, 0.056), temporo-occipital inferior temporal gyrus (slope, −0.006 [95% CI, −0.009 to −0.003]; partial η^2^, 0.050), posterior temporal fusiform cortex (slope, −0.006 [95% CI, −0.009 to −0.003]; partial η^2^, 0.048), and posterior middle temporal gyrus (slope, −0.006 [95% CI, −0.009 to −0.003]; partial η^2^, 0.045). There was a significant interaction between tau-positivity vs tau-negativity and time in 5 of 16 ROIs, with small effect sizes (partial η^2^, 0.021-0.083) (Figure 4). CI individuals showed significantly accelerated atrophy in 5 of 16 ROIs relative to CU individuals, with negligible to small effect sizes (partial η^2^, 0.014-0.025). eFigures 10 and 11 and eTable 9 in Supplement 1 give full depictions of Aβ+ duration and tau-positive results. There was no main effect of Aβ+ duration or tau-positivity and a few main effects of diagnosis (eTable 10 in Supplement 1).

### Sensitivity Analyses

Our results remained largely unchanged with the exclusion of CI individuals. Both Aβ+ and Aβ+ duration were associated with accelerated atrophy, controlling for tau, and there was no main effect of either (eFigures 12-15 and eTables 11-14 in Supplement 1). The results were similar when subtracting baseline volumes from longitudinal trajectories (eFigures 16-19 and eTables 15-18 in Supplement 1).

### Replication Analyses

eTable 19 in Supplement 1 provides sample characteristics, and eTables 20 and 21 in Supplement 1 give *z* scoring model outputs. eFigure 20 in Supplement 1 includes N+ counts by ROI in the initial *z* scoring sample. See eFigures 21 and 22 in Supplement 1 for uncorrected volumes and *z*-scored volumes by ROI in the final *z*-scoring sample. The results were largely similar in the OASIS-3 dataset. Baseline Aβ+ was associated with accelerated atrophy in 14 of 16 ROIs, controlling for tau-positivity. Unlike the primary analysis, baseline Aβ+ duration was associated with change in fewer ROIs (6 of 16) than binary Aβ+ and Aβ−. eFigures 23 and 24 and eTables 22 to 25 in Supplement 1 provide the full results from the Aβ+ and Aβ+ duration analyses.

## Discussion

This cohort study examined the association between Aβ pathology and duration of Aβ plaque pathology as measured by Aβ PET imaging with longitudinal atrophy in a sample of predominantly CU individuals. Our results indicated that Aβ+ individuals showed more rapid volume loss than AΒ− individuals in several medial temporal lobe and neocortical regions, even after controlling for individuals who were later observed to be tau-positive. The same result was observed in an independent replication cohort drawn from the OASIS-3 dataset. Furthermore, continuous Aβ+ duration was associated with broader changes in the expected AD-like pattern, although this was not the case in the replication dataset. Notably, accelerated atrophy was observed in Aβ+ individuals in the absence of CI. These results suggest that even among a predominantly CU sample, Aβ+ is associated with abnormal atrophy in brain regions consistent with an AD-like pattern of atrophy that may worsen with longer exposure to abnormal Aβ accumulation independently of tau.

These results agree with prior research suggesting that neurodegeneration in AD affects temporo-parietal regions that generally reflect the spread of tau neurofibrillary tangle pathology (NFT).^34^ Studies have shown a longitudinal spread of atrophy from the medial temporal lobe to the broader neocortex in the progression from MCI to AD.^35^ Similar longitudinal atrophy has been observed in Aβ+ CU individuals as well.^36,37^ Studies that have examined longitudinal atrophy with Aβ and tau PET have found that tau is associated with stronger, more spatially extensive atrophy.^4,5^ This finding suggests a relationship between Aβ deposition and neurodegeneration that depends primarily on tau. In the present analyses, we showed a similar pattern of temporo-parietal neurodegeneration alongside Aβ+ and Aβ+ duration, but changes persisted even after controlling for subsequent tau-positivity in the entorhinal cortex. Taken together, these results suggest an association between early Aβ pathology and atrophy in tau-accumulating regions that precedes the development of NFTs. It is plausible that atrophy in AD occurs in 2 phases: subtle atrophy following Aβ+, possibly mediated by increases in glial activation^38^ or prefilamentous tau hyperphosphorylation in distant, tangle-bearing regions,^39,40^ followed by stronger atrophy with longer Aβ+ duration and the onset of NFT pathology in these ROIs.

Previous work from members of our group has shown that increasing Aβ+ duration is associated with faster cognitive decline, a greater risk of cognitive impairment, and the spread of NFT accumulation from early Braak stage regions to later ones.^20,34,41^ Our study complements these findings by showing that harboring Aβ plaques for longer is also associated with accelerated volume loss, even after controlling for tau. To date, studies examining the association between AD pathology and longitudinal atrophy have not defined abnormal Aβ in the time domain. Given the long preclinical window of AD, understanding when atrophic change occurs based on how long an individual has been exposed to abnormal Aβ accumulation will be key for future treatment efforts. Most strikingly, we showed here that significant atrophy was detectable in individuals with generally short Aβ+ duration (median [IQR], 3.6 [1.2-7.5] years). This finding suggests that critical atrophic processes, which are associated with an increased risk of cognitive decline and dementia,^42,43^ may occur early in the time course of Aβ accumulation, possibly necessitating clinical intervention within the first decade of Aβ+.

### Limitations

This study has limitations. First, our sample consisted predominantly of non-Hispanic, White individuals. Second, baseline tau measures would have been useful to more accurately parse contributions of tau. Furthermore, previous work from members of our group and others has shown spatial variability in the progression of tau pathology, which may not be present in the entorhinal cortex in all cases.^44,45^ Additionally, our study did not take into account non-AD neuropathological conditions, such as vascular disease, limbic-predominant age-related TDP-43 encephalopathy neuropathologic change,^46^ or Lewy body disease,^47^ that may have added to atrophy in an additive or synergistic fashion.^48,49^

## Conclusions

This cohort study found that among a predominantly CU sample, Aβ+ and Aβ+ duration were associated with subtle neurodegeneration in and beyond the medial temporal lobe, even controlling for tau-positivity. These results expand existing models of neurodegeneration in AD and suggest that abnormal Aβ deposition itself may be associated with neurodegenerative change, although more research is needed to understand its pathological nature.

## Notes

Supplement 1. **eMethods 1.** Longitudinal volumetric MRI **eMethods 2.** Robust Normative z-scoring **eMethods 3.** Replication analysis **eFigure 1.** Flowchart for selected participants **eTable 1.** T1-weighted MRI acquisition details **eTable 2.** T1-weighted MRI scanner and head coil-specific counts **eTable 3.** Sample characteristics of the group used for SILA modeling **eFigure 2.** SILA-based modeling of PiB PET data **eFigure 3.** Flowchart for selected participants in the OASIS-3 dataset **eTable 4.** Sample characteristics of the group used for SILA modeling in the OASIS-3 dataset **eFigure 4.** SILA-based modeling of Aβ PET data in the OASIS-3 dataset **eTable 5.** Results from initial z-scoring model **eTable 6.** Results from final robust z-scoring model **eFigure 5.** Regional counts of N+ among A- individuals **eFigure 6.** Unadjusted volumes in the final z-scoring sample **eFigure 7.** Z-scored volumes in the final z-scoring sample **eFigure 8.** Regional effects of Aβ+ **eFigure 9.** Regional effects of T+ **eTable 7.** Results for each interaction term (Aβ+/- analysis) **eTable 8.** Results for each main effect (Aβ+/- analysis) **eFigure 10.** Regional effects of Aβ+ duration **eFigure 11.** Regional effects of T+ from the Aβ+ duration analysis **eTable 9.** Results for each interaction term (Aβ+ duration analysis) **eTable 10.** Results for each main effect (Aβ+ duration analysis) **eFigure 12.** Regional effects of Aβ+ with CU individuals only **eFigure 13.** Regional effects of T+ with CU individuals only **eTable 11.** Results for each interaction term (Aβ+/- analysis) with CU individuals only **eTable 12.** Results for each main effect (Aβ+/- analysis) with CU individuals only **eFigure 14.** Regional effects of Aβ+ duration in CU individuals only **eFigure 15.** Regional effects of T+ from the Aβ+ duration analysis with CU individuals only **eTable 13.** Results for each interaction term (Aβ+ duration analysis) in CU individuals only **eTable 14.** Results for each main effect (Aβ+ duration analysis) in CU individuals only **eFigure 16.** Regional effects of Aβ+ with baseline volumes subtracted **eFigure 17.** Regional effects of T+ with baseline volumes subtracted **eTable 15.** Results for each interaction term (Aβ+/- analysis) with baseline volumes subtracted **eTable 16.** Results for each main effect (Aβ+/- analysis) with baseline volumes subtracted **eFigure 18.** Regional effects of Aβ+ duration with baseline volumes subtracted **eFigure 19.** Regional effects of T+ from the Aβ+ duration analysis with baseline volumes subtracted **eTable 17.** Results for each interaction term (Aβ+ duration analysis) with baseline volumes subtracted **eTable 18.** Results for each main effect (Aβ+ duration analysis) with baseline volumes subtracted **eTable 19.** Sample characteristics of the OASIS-3 participants used for the replication analysis **eTable 20.** Results from initial z-scoring model in the OASIS-3 dataset **eTable 21.** Results from final robust z-scoring model in the OASIS-3 dataset **eFigure 20.** Regional counts of N+ among A- individuals in the OASIS-3 dataset **eFigure 21.** Unadjusted volumes in the final z-scoring sample in the OASIS-3 dataset **eFigure 22.** Z-scored volumes in the final z-scoring sample in the OASIS-3 dataset **eFigure 23.** Results from the Aβ+ analysis in the OASIS-3 dataset **eFigure 24.** Results from the Aβ+ duration analysis in the OASIS-3 dataset **eTable 22.** Results for each interaction term in the OASIS-3 dataset (Aβ+/- analysis) **eTable 23.** Results for each main effect in the OASIS-3 dataset (Aβ+/- analysis) **eTable 24.** Results for each interaction term in the OASIS-3 dataset (Aβ+ duration analysis) **eTable 25.** Results for each main effect in the OASIS-3 dataset (Aβ+ duration analysis)

Supplement 2.
Data Sharing Statement
