# Supplementary material for: Abnormal Amyloid-β Duration, Tau, and Neurodegeneration in Cranial Images
Source: JAMA Netw Open. 2025 Sep 9;8(9):e2531093. doi: 10.1001/jamanetworkopen.2025.31093 (PMC12421349; doi:10.1001/jamanetworkopen.2025.31093)
Supplement: Supplement 1. — eMethods 1. Longitudinal volumetric MRI eMethods 2. Robust Normative z-scoring eMethods 3. Replication analysis eFigure 1. Flowchart for selected participants eTable 1. T1-weighted MRI acquisition details eTable 2. T1-weighted MRI scanner and head coil-specific counts eTable 3. Sample characteristics of the group used for SILA modeling eFigure 2. SILA-based modeling of PiB PET data eFigure 3. Flowchart for selected participants in the OASIS-3 dataset eTable 4. Sample characteristics of the group used for SILA modeling in the OASIS-3 dataset eFigure 4. SILA-based modeling of Aβ PET data in the OASIS-3 dataset eTable 5. Results from initial z-scoring model eTable 6. Results from final robust z-scoring model eFigure 5. Regional counts of N+ among A- individuals eFigure 6. Unadjusted volumes in the final z-scoring sample eFigure 7. Z-scored volumes in the final z-scoring sample eFigure 8. Regional effects of Aβ+ eFigure 9. Regional effects of T+ eTable 7. Results for each interaction term (Aβ+/- analysis) eTable 8. Results for each main effect (Aβ+/- analysis) eFigure 10. Regional effects of Aβ+ duration eFigure 11. Regional effects of T+ from the Aβ+ duration analysis eTable 9. Results for each interaction term (Aβ+ duration analysis) eTable 10. Results for each main effect (Aβ+ duration analysis) eFigure 12. Regional effects of Aβ+ with CU individuals only eFigure 13. Regional effects of T+ with CU individuals only eTable 11. Results for each interaction term (Aβ+/- analysis) with CU individuals only eTable 12. Results for each main effect (Aβ+/- analysis) with CU individuals only eFigure 14. Regional effects of Aβ+ duration in CU individuals only eFigure 15. Regional effects of T+ from the Aβ+ duration analysis with CU individuals only eTable 13. Results for each interaction term (Aβ+ duration analysis) in CU individuals only eTable 14. Results for each main effect (Aβ+ duration analysis) in CU individuals only eFigure 16. Regional effects of Aβ+ with baseline volume [file jamanetwopen-e2531093-s001.pdf]

# Supplementary Online Content

Stephenson HG, Langhough R, Jonaitis E, et al. Abnormal amyloid-β duration, tau, and neurodegeneration in cranial images. *JAMA Netw Open*. 2025;8(9):e2531093. doi:10.1001/jamanetworkopen.2025.31093

**eMethods 1.** Longitudinal volumetric MRI

**eMethods 2.** Robust Normative z-scoring

**eMethods 3.** Replication analysis

**eFigure 1.** Flowchart for selected participants

**eTable 1.** T1-weighted MRI acquisition details

**eTable 2.** T1-weighted MRI scanner and head coil-specific counts

**eTable 3.** Sample characteristics of the group used for SILA modeling

**eFigure 2.** SILA-based modeling of PiB PET data

**eFigure 3.** Flowchart for selected participants in the OASIS-3 dataset

**eTable 4.** Sample characteristics of the group used for SILA modeling in the OASIS-3 dataset

**eFigure 4.** SILA-based modeling of Aβ PET data in the OASIS-3 dataset

**eTable 5.** Results from initial z-scoring model

**eTable 6.** Results from final robust z-scoring model

**eFigure 5.** Regional counts of N+ among A- individuals

**eFigure 6.** Unadjusted volumes in the final z-scoring sample

**eFigure 7.** Z-scored volumes in the final z-scoring sample

**eFigure 8.** Regional effects of A+

**eFigure 9.** Regional effects of T+

**eTable 7.** Results for each interaction term (A+/- analysis)

**eTable 8.** Results for each main effect (A+/- analysis)

**eFigure 10.** Regional effects of A+ duration

**eFigure 11.** Regional effects of T+ from the A+ duration analysis

© 2025 Stephenson HG et al. *JAMA Network Open*.

**eTable 9.** Results for each interaction term (A+ duration analysis)

**eTable 10.** Results for each main effect (A+ duration analysis)

**eFigure 12.** Regional effects of A+ with CU individuals only

**eFigure 13.** Regional effects of T+ with CU individuals only

**eTable 11.** Results for each interaction term (A+/- analysis) with CU individuals only

**eTable 12.** Results for each main effect (A+/- analysis) with CU individuals only

**eFigure 14.** Regional effects of A+ duration in CU individuals only

**eFigure 15.** Regional effects of T+ from the A+ duration analysis with CU individuals only

**eTable 13.** Results for each interaction term (A+ duration analysis) in CU individuals only

**eTable 14.** Results for each main effect (A+ duration analysis) in CU individuals only

**eFigure 16.** Regional effects of A+ with baseline volumes subtracted

**eFigure 17.** Regional effects of T+ with baseline volumes subtracted

**eTable 15.** Results for each interaction term (A+/- analysis) with baseline volumes subtracted

**eTable 16.** Results for each main effect (A+/- analysis) with baseline volumes subtracted

**eFigure 18.** Regional effects of A+ duration with baseline volumes subtracted

**eFigure 19.** Regional effects of T+ from the A+ duration analysis with baseline volumes subtracted

**eTable 17**. Results for each interaction term (A+ duration analysis) with baseline volumes subtracted

**eTable 18.** Results for each main effect (A+ duration analysis) with baseline volumes subtracted

**eTable 19.** Sample characteristics of the OASIS-3 participants used for the replication analysis

**eTable 20.** Results from initial z-scoring model in the OASIS-3 dataset

**eTable 21.** Results from final robust z-scoring model in the OASIS-3 dataset

**eFigure 20.** Regional counts of N+ among A- individuals in the OASIS-3 dataset

© 2025 Stephenson HG et al. *JAMA Network Open*.

**eFigure 21.** Unadjusted volumes in the final z-scoring sample in the OASIS-3 dataset

**eFigure 22.** Z-scored volumes in the final z-scoring sample in the OASIS-3 dataset

**eFigure 23.** Results from the A+ analysis in the OASIS-3 dataset

**eFigure 24.** Results from the A+ duration analysis in the OASIS-3 dataset

**eTable 22.** Results for each interaction term in the OASIS-3 dataset (A+/- analysis)

**eTable 23.** Results for each main effect in the OASIS-3 dataset (A+/- analysis)

**eTable 24.** Results for each interaction term in the OASIS-3 dataset (A+ duration analysis)

**eTable 25.** Results for each main effect in the OASIS-3 dataset (A+ duration analysis)

**eReferences.**

This supplementary material has been provided by the authors to give readers additional information about their work.

© 2025 Stephenson HG et al. *JAMA Network Open*.

## eMethods 1. Longitudinal volumetric MRI

A longitudinal pipeline in SPM12 was implemented to ensure accurate, consistent processing of images within-individuals. Two or more longitudinal images per participant were non-linearly registered to a within-participant average image in an unbiased manner using Serial Longitudinal Registration[1]. The resultant average image was then segmented using Unified Segmentation to provide longitudinal tissue probability maps (TPM) of gray matter (GM), white matter (WM), and CSF[2]. The TPMs were then non-linearly coregistered with an MNI152-space template using Population to ICBM registration in the DARTEL toolbox to obtain coregistration with the Harvard-Oxford cortical and subcortical atlases[3]. TPMs and Harvard-Oxford atlases were then projected back to the native space of each longitudinal image using inverse MNI and longitudinal warps, and a light 2mm FWHM smoothing kernel was applied to the warped longitudinal TPMs. In the native space, images were segmented using Unified Segmentation with coregistered longitudinal TPMs as priors. ROI-level volumes were derived by taking the sum of GM probabilities within ROIs. Total intracranial volume (ICV) was derived by calculating total volume of SPM12's ICV mask in native space. The mean of these volumes collected at each longitudinal time point was used within participants as their ICV.

MRIs from the OASIS-3 dataset used in the replication analysis were processed under a highly similar longitudinal pipeline with two main differences: 1) there was no segmentation for the individual timepoints. GM volume at each time point was taken from the longitudinal GM TPM warped to native space. 2) ICV was taken from the average image only, and this value was used to model ICV.

## eMethods 2. Robust normative z-scoring

We used a robust normative approach to identify an A-T- CU sample free from abnormal atrophy and z-score volumes according to age-related changes in this sample. Visits within 244 A-T- CU individuals were randomly selected from each individual's longitudinal dataset. Random selection was done to ensure adequate sampling of both head coils given that most individuals switched from the 8-channel to the 32-channel coil during their observation period. ROI volumes were entered into a linear regression predicting regional volumes with age, sex, intracranial volume (ICV), and head coil as covariates. Parameter estimates from these models and their root mean square error (RMSE) were then used to z-score regional volumes using the following formula:

Z-Scored Volume = (Observed Volume – Predicted Volume) / RMSE

To isolate a robust normative sample, we removed A-T- CU individuals with z-scored volumes < -2 SD in one or more ROIs. The above model was applied again to unadjusted volumes from this reduced sample. The resultant parameter estimates and RMSE's were used to z-score volumes for the entire sample. Higher order terms were added as appropriate.

In this case, z-scores represent abnormality relative to what we would predict given an individual's demographics and details of their image acquisition. An individual with an observed volume equal to their predicted volume would have a z-score of 0, suggesting normal volume given their age, controlling for sex, ICV, and head coil. If an individual had a lower volume than predicted, their score would be negative, suggesting abnormal volume given their age, sex, ICV, and head coil. Longitudinally, if an individual's atrophy did not exceed the age-related atrophy predicted by the model, their score would remain at 0. If their volume loss exceeded predicted age-related volume loss, their score would become more negative over time.

We also investigated non-linearity in age-related decline[4] and modification of age-related atrophy by sex[5] in the final control sample. All models were FDR-corrected. The quadratic effect of age was significant in the hippocampus and amygdala as well as the posterior parahippocampal gyrus and temporal-occipital fusiform gyrus such that the effect of age was worse at older ages. Therefore, the term was added to z-scoring models for these ROIs. The interaction between age and sex was not significant in any ROI.

## eMethods 3. Replication analysis

Because OASIS-3 includes both 11C-PiB PET and 18F-AV-45 (florbetapir) PET data, SILA was run using a CL value of 17.1 for global Aβ burden as opposed to SUVR or DVR. T+ was defined according to an SUVR of ≥ 1.33. This cut-point was derived via Gaussian mixture modeling of entorhinal tau levels in the mixtools package[6]. FreeSurfer ROIs used to derive global Aβ CL and entorhinal tau. See LaMontagne et al. for details.[7]

© 2025 Stephenson HG et al. *JAMA Network Open*.

Z-scoring methods were mostly identical for the replication analysis in the OASIS-3 dataset. All T1-weighted images in the dataset were collected using a 16-channel head coil. As a result, age, sex,  and ICV were the only covariates used for z-scoring in this sample. There were no non-linear effects of age. There was an interaction between age and sex in the temporal pole.

© 2025 Stephenson HG et al. *JAMA Network Open*.

**eFigure 1.** Flowchart for selected participants.

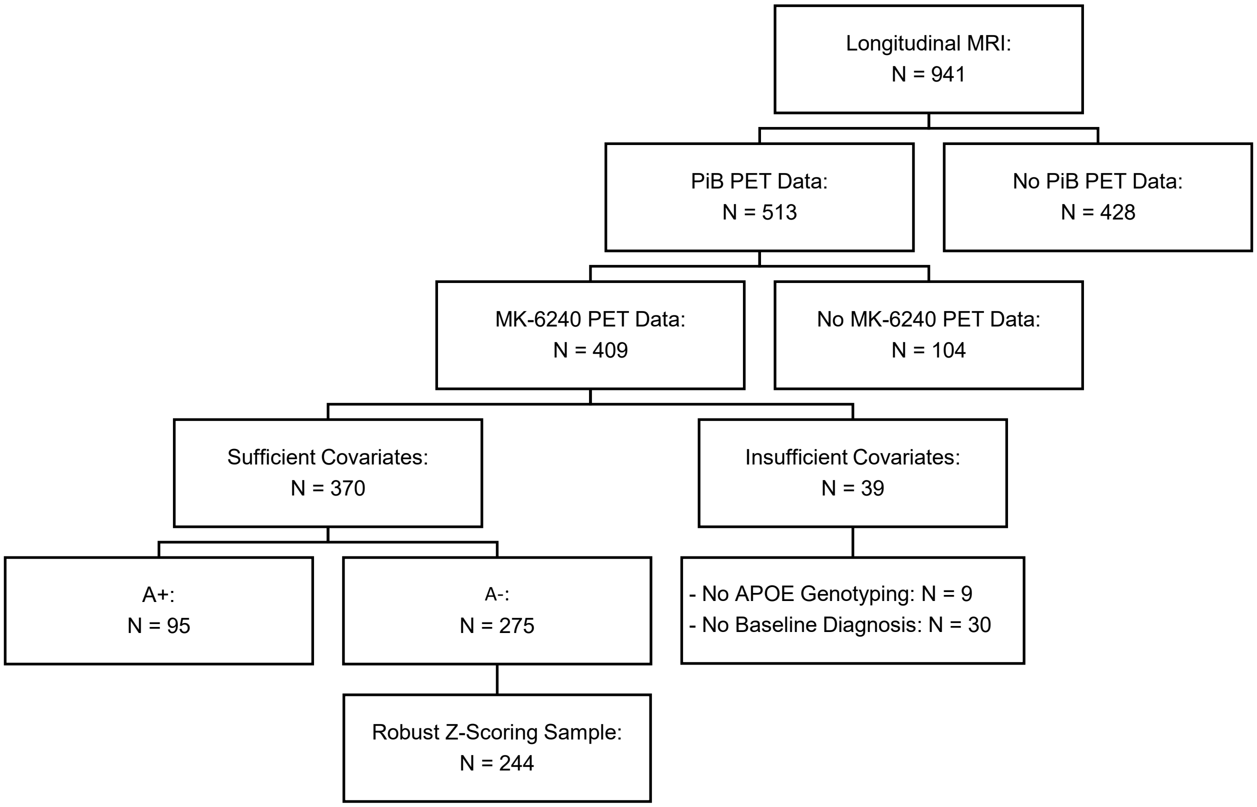


© 2025 Stephenson HG et al. *JAMA Network Open*.

**eTable 1.** T1-weighted MRI acquisition details.

| Series | Scanning Sequence | Sequence Variant | TR | TE | TI | Slice Thickness | Flip Angle | Acquisition Matrix | Field of View | N |
|---|---|---|---|---|---|---|---|---|---|---|
| 1 | GR | SS/SP/SK | 7.35 | 3.04 | 400 | 1 | 11 | 0/256/256/0 | 100 | 311 |
| 2 | GR | SS/SK | 8.16 | 3.18 | 450 | 1 | 12 | 0/256/256/0 | 100 | 786 |
| 3 | GR | SS/SK | 6.68 | 2.94 | 450 | 1 | 12 | 0/256/256/0 | 100 | 226 |
| 5 | GR | SS/SP/SK | 6.68 | 2.94 | 400 | 1 | 11 | 0/256/256/0 | 100 | 196 |

**eTable 2.** T1-weighted MRI scanner and head coil-specific counts.

| MRI Scanner | MRI Head Coil | N |
|---|---|---|
| GE MR750 #1 | 8-channel Excite (GE Healthcare) | 129 |
| GE MR750 #1 | 32-channel (Nova Medical) | 274 |
| GE MR750 #2 | 8-channel Excite (GE Healthcare) | 544 |
| GE MR750 #2 | 32-channel (Nova Medical) | 572 |

**eTable 3.** Sample characteristics of the group used for SILA modeling.

| N | % Female | Age at Last PET Scan | Length of Follow-up (Years), mean(SD) | Number of Visits, mean(SD) |
|---|---|---|---|---|
| 507 | 344 | 69.51(7.35) | 4.78(4.07) | 2.92(1.16) |

© 2025 Stephenson HG et al. *JAMA Network Open*.

## eFigure 2. SILA-based modeling of PiB PET data

**A**: Observed longitudinal PiB PET DVR values used for SILA modeling plotted according to age at scan. Horizontal black lines represent the A+ threshold of 1.16 DVR. **B**: Observed longitudinal PiB PET DVR values plotted according to estimated A+ duration at each PiB PET scan. Note that only each participant's most recent value was used to model duration.

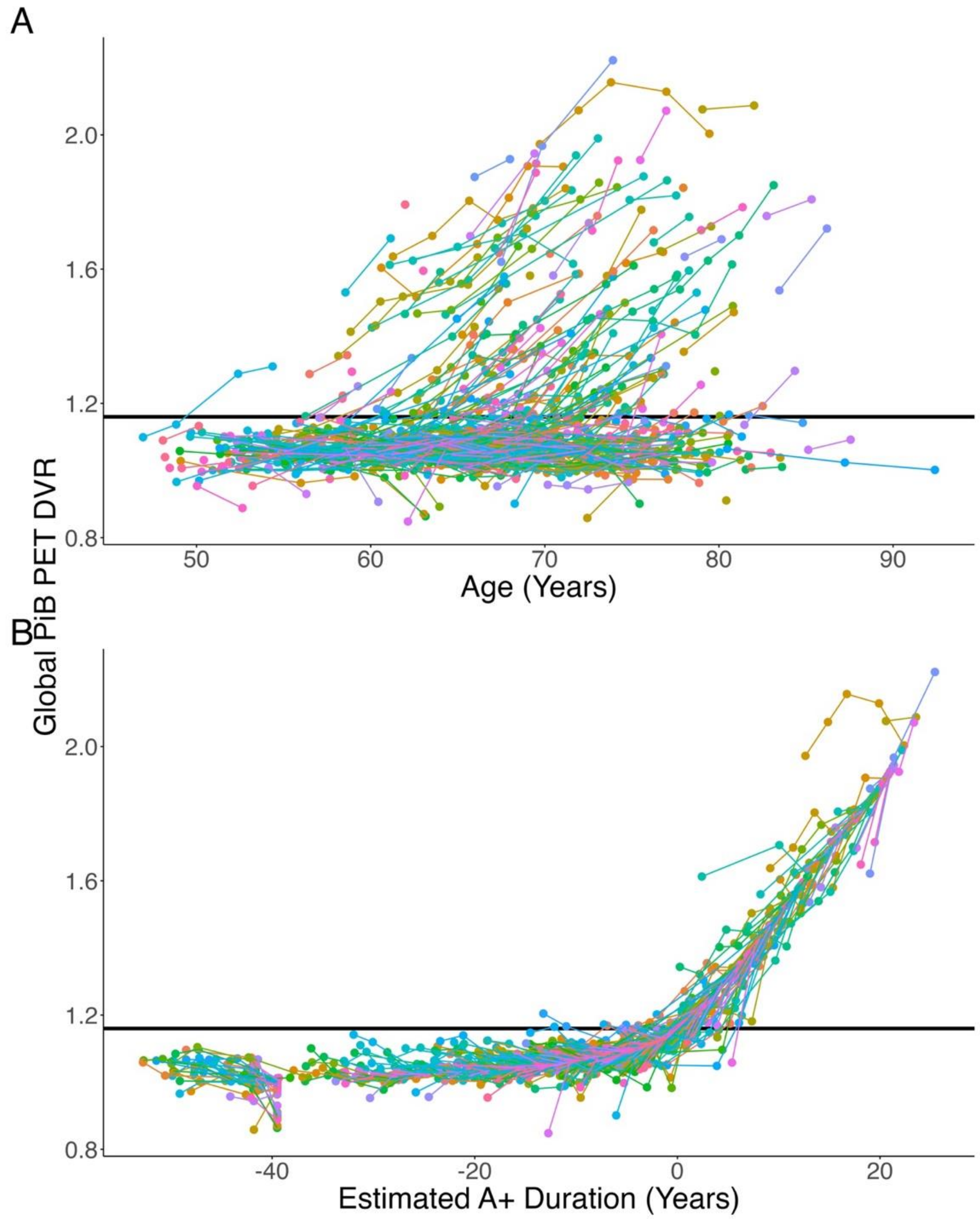


© 2025 Stephenson HG et al. *JAMA Network Open*.

**eFigure 3.** Flowchart for selected participants from the OASIS-3 dataset

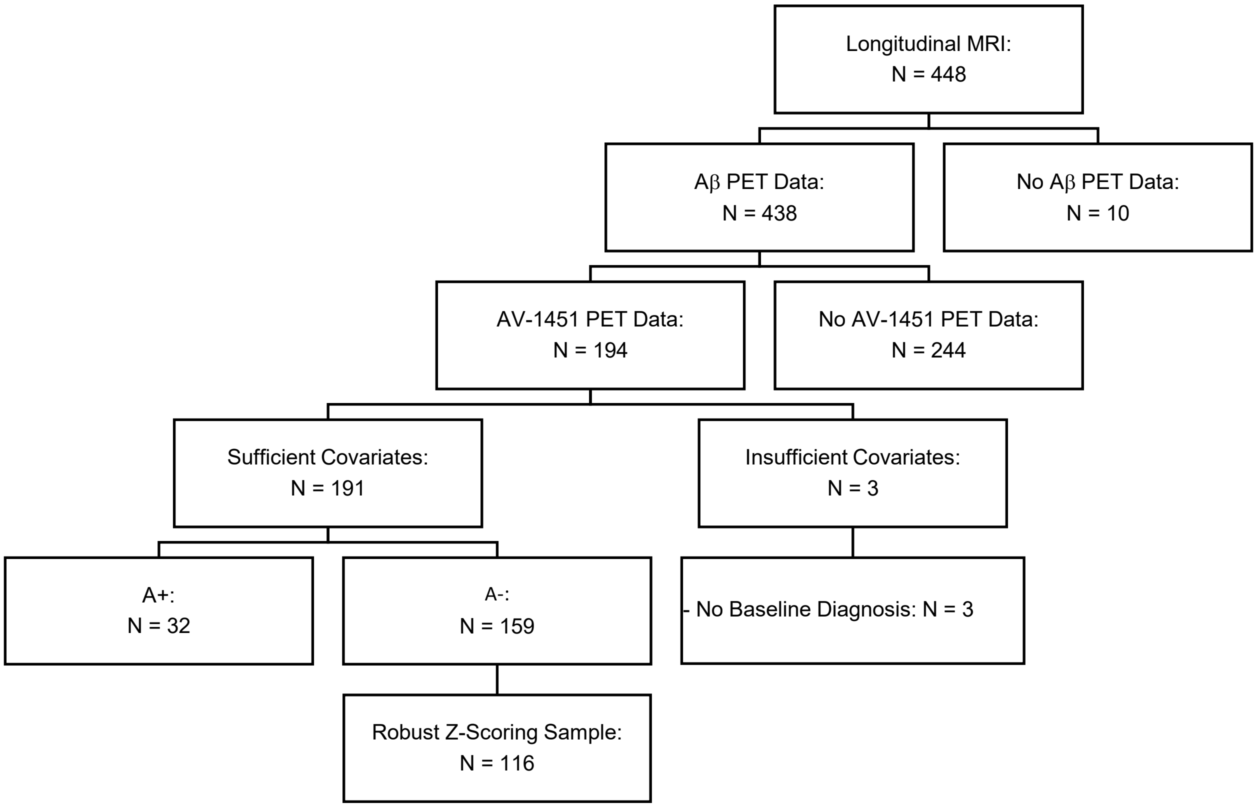


© 2025 Stephenson HG et al. *JAMA Network Open*.

**eTable 4.** Sample characteristics of the group used for SILA modeling. FBP: florbetapir

| N | % Female | Age at Last PET | Length of Follow-up (Years), mean(SD) | Number of Visits, mean(SD) | No. PiB/FBP Scans |
|---|---|---|---|---|---|
| 357 | 62.47% | 72.29(7.85) | 5.48(2.97) | 2.97(1.15) | 679/378 |

© 2025 Stephenson HG et al. *JAMA Network Open*.

**eFigure 4.** SILA-based modeling of Aβ PET data in the OASIS-3 dataset

**A**: Observed longitudinal Aβ PET CL values used for SILA modeling plotted according to age at scan. Horizontal black lines represent the A+ threshold of 17.1 CL. **B**: Observed longitudinal Aβ PET CL values plotted according to estimated A+ duration at each Aβ PET scan. Note that only each participant's most recent value was used to model duration.

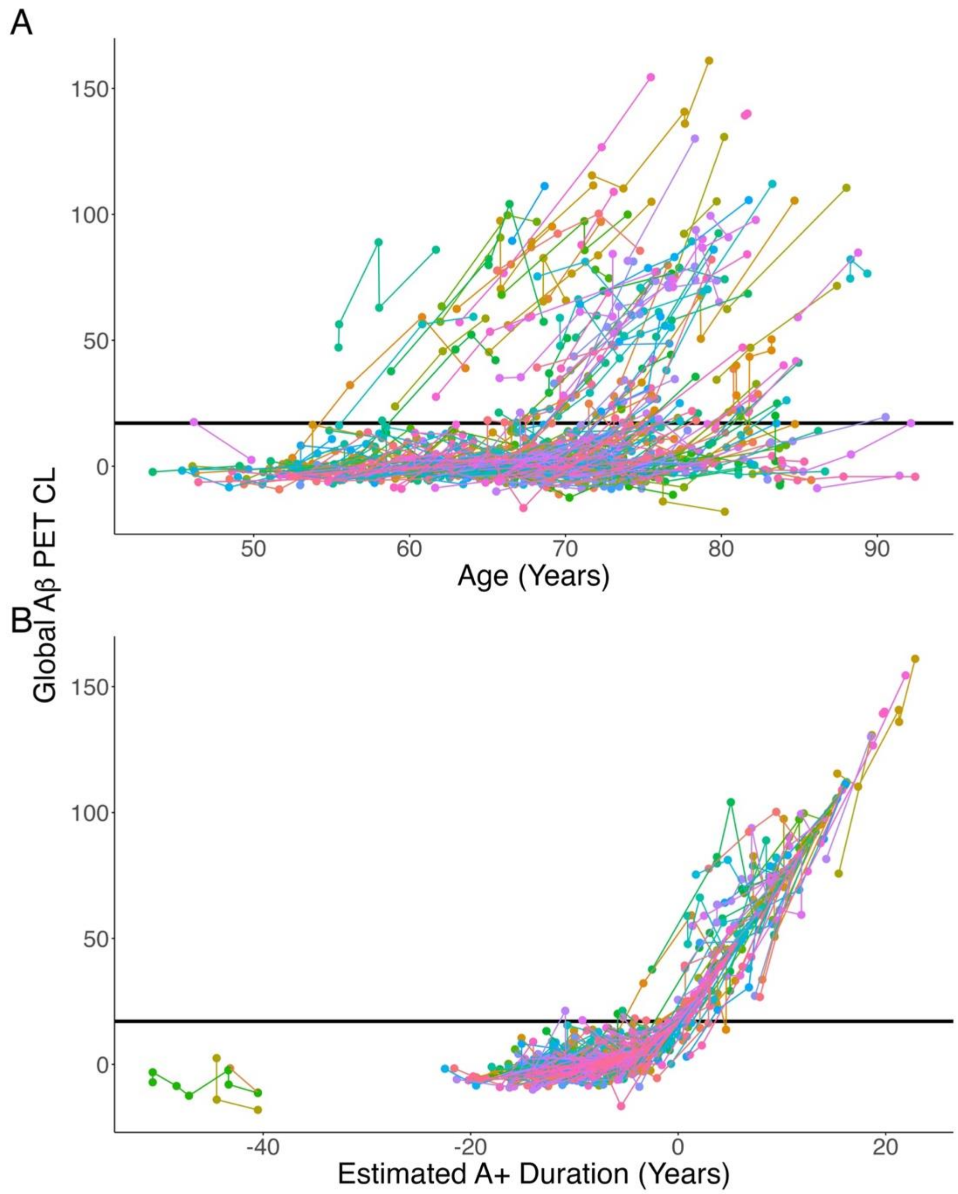


© 2025 Stephenson HG et al. *JAMA Network Open*.

**eTable 5.** Results from initial z-scoring model. Bolded values signify significance at $p < 0.05$ (FDR-corrected).

| ROI | Unadjusted Volume (SD) | $\beta_{int}$ | $\beta_{sex}$ | $\beta_{ICV}$ | $\beta_{head\ coil}$ | $\beta_{age}$ | RMSE |
|---|---|---|---|---|---|---|---|
| AG | 4.324(0.55) | **3.826** | -0.111 | **0.001** | **0.19** | **-0.029** | 0.452 |
| ITGA | 1.761(0.28) | **1.385** | **0.21** | **0** | 0.006 | **-0.006** | 0.234 |
| ITGP | 4.635(0.61) | **3.732** | 0.167 | **0.002** | -0.121 | **-0.023** | 0.489 |
| ITGT | 3.395(0.45) | **2.311** | **0.151** | **0.001** | 0.055 | **-0.016** | 0.343 |
| MTGA | 1.808(0.27) | **1.279** | 0.042 | **0.001** | -0.02 | **-0.007** | 0.237 |
| MTGP | 5.226(0.65) | **5.152** | 0.023 | **0.002** | 0.065 | **-0.035** | 0.542 |
| MTGT | 3.803(0.47) | **3.442** | 0.05 | **0.001** | 0.048 | **-0.024** | 0.38 |
| PHGA | 4.986(0.59) | **4.054** | 0.139 | **0.002** | 0.126 | **-0.031** | 0.427 |
| PHGP | 2.712(0.29) | **2.594** | 0.037 | **0.001** | 0.008 | **-0.017** | 0.219 |
| PREC | 9.758(1.28) | **8.48** | -0.082 | **0.004** | -0.119 | **-0.066** | 1 |
| TFCA | 1.714(0.3) | **1.401** | **0.132** | **0.001** | 0.026 | **-0.011** | 0.245 |
| TFCP | 4.177(0.49) | **3.678** | **0.251** | **0.001** | 0.063 | **-0.025** | 0.353 |
| TFCO | 4.322(0.48) | **4.071** | **0.186** | **0.001** | **0.145** | **-0.028** | 0.351 |
| TP | 9.786(1.3) | **7.191** | **0.601** | **0.003** | 0.108 | **-0.024** | 1.121 |
| HIPP | 3.69(0.44) | **4.099** | 0.03 | **0.001** | 0.084 | **-0.027** | 0.37 |
| AMYG | 1.736(0.21) | **1.793** | 0.03 | **0.001** | 0.038 | **-0.013** | 0.166 |

**eTable 6.** Results from final robust z-scoring model. Bolded values signify significance at $p < 0.05$ (FDR-corrected).

| ROI | Unadjusted Volume (SD) | $\beta_{int}$ | $\beta_{sex}$ | $\beta_{ICV}$ | $\beta_{head\ coil}$ | $\beta_{age}$ | $\beta_{age^2}$ | RMSE |
|---|---|---|---|---|---|---|---|---|
| AG | 4.381(0.524) | **3.444** | -0.14 | **0.002** | **0.222** | **-0.03** | | 0.392 |
| ITGA | 1.791(0.277) | **1.171** | **0.183** | **0.001** | -0.007 | **-0.007** | | 0.213 |
| ITGP | 4.705(0.59) | **2.995** | 0.044 | **0.002** | -0.13 | **-0.023** | | 0.422 |
| ITGT | 3.426(0.429) | **1.917** | 0.108 | **0.002** | 0.055 | **-0.015** | | 0.302 |
| MTGA | 1.835(0.263) | **1.113** | 0.021 | **0.001** | -0.007 | **-0.007** | | 0.222 |
| MTGP | 5.311(0.62) | **4.694** | -0.083 | **0.002** | 0.116 | **-0.037** | | 0.47 |
| MTGT | 3.849(0.451) | **3.103** | -0.004 | **0.002** | 0.069 | **-0.024** | | 0.337 |
| PHGA | 5.045(0.565) | **3.309** | 0.057 | **0.002** | 0.129 | **-0.028** | | 0.361 |
| PHGP | 2.741(0.272) | -0.424 | 0.009 | **0.001** | 0.037 | **0.069** | **-0.0006** | 0.179 |
| PREC | 9.908(1.196) | **7.96** | -0.253 | **0.004** | -0.079 | **-0.07** | | 0.847 |
| TFCA | 1.738(0.295) | **1.039** | **0.104** | **0.001** | 0.013 | **-0.011** | | 0.224 |
| TFCP | 4.231(0.474) | **2.917** | **0.162** | **0.002** | 0.072 | **-0.024** | | 0.286 |
| TFCO | 4.365(0.455) | 0.504 | **0.157** | **0.002** | **0.19** | **0.074** | **-0.0007** | 0.29 |
| TP | 9.91(1.254) | **6.139** | 0.41 | **0.004** | 0.108 | **-0.026** | | 1.031 |
| HIPP | 3.744(0.395) | -1.687 | -0.019 | **0.001** | 0.092 | **0.141** | **-0.001** | 0.299 |
| AMYG | 1.762(0.193) | -0.219 | 0.005 | **0.001** | 0.045 | **0.044** | **-0.0004** | 0.135 |

© 2025 Stephenson HG et al. *JAMA Network Open*.

**eFigure 5.** Regional counts of N+ among A- individuals

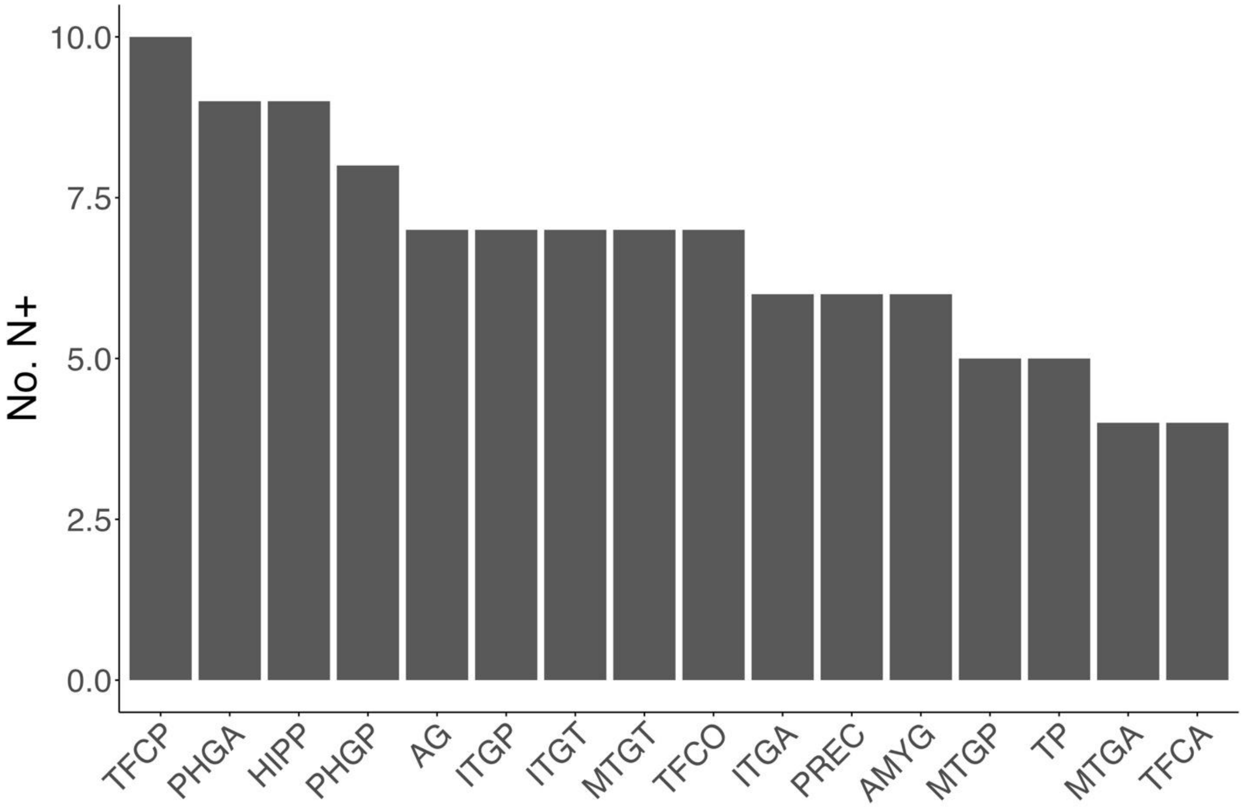


© 2025 Stephenson HG et al. *JAMA Network Open*.

**eFigure 6.** Unadjusted volumes in the final z-scoring sample. Separate lines and colors denote different participants.

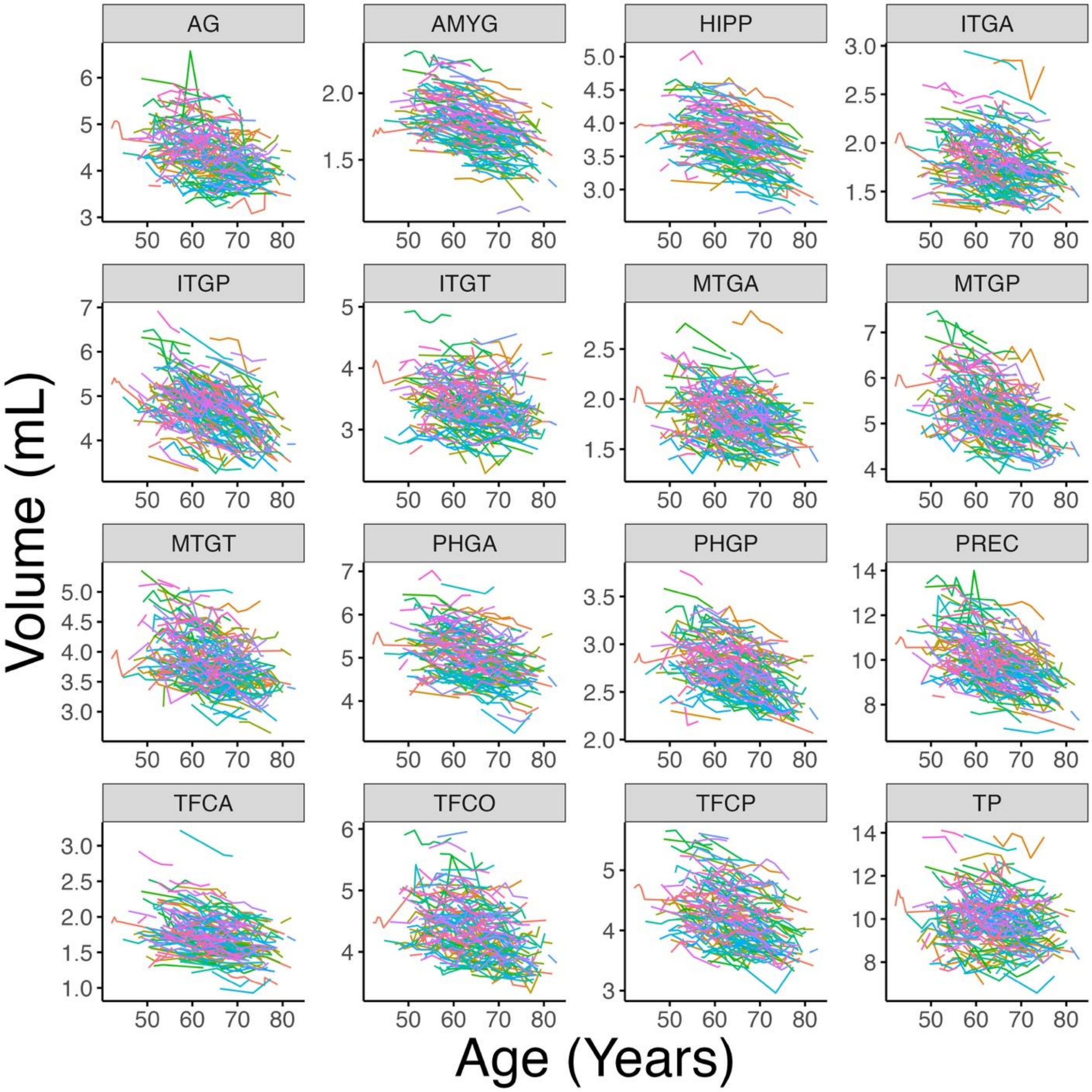


© 2025 Stephenson HG et al. *JAMA Network Open*.

**eFigure 7.** Z-scored volumes in the final z-scoring sample. Separate lines and colors denote different participants.

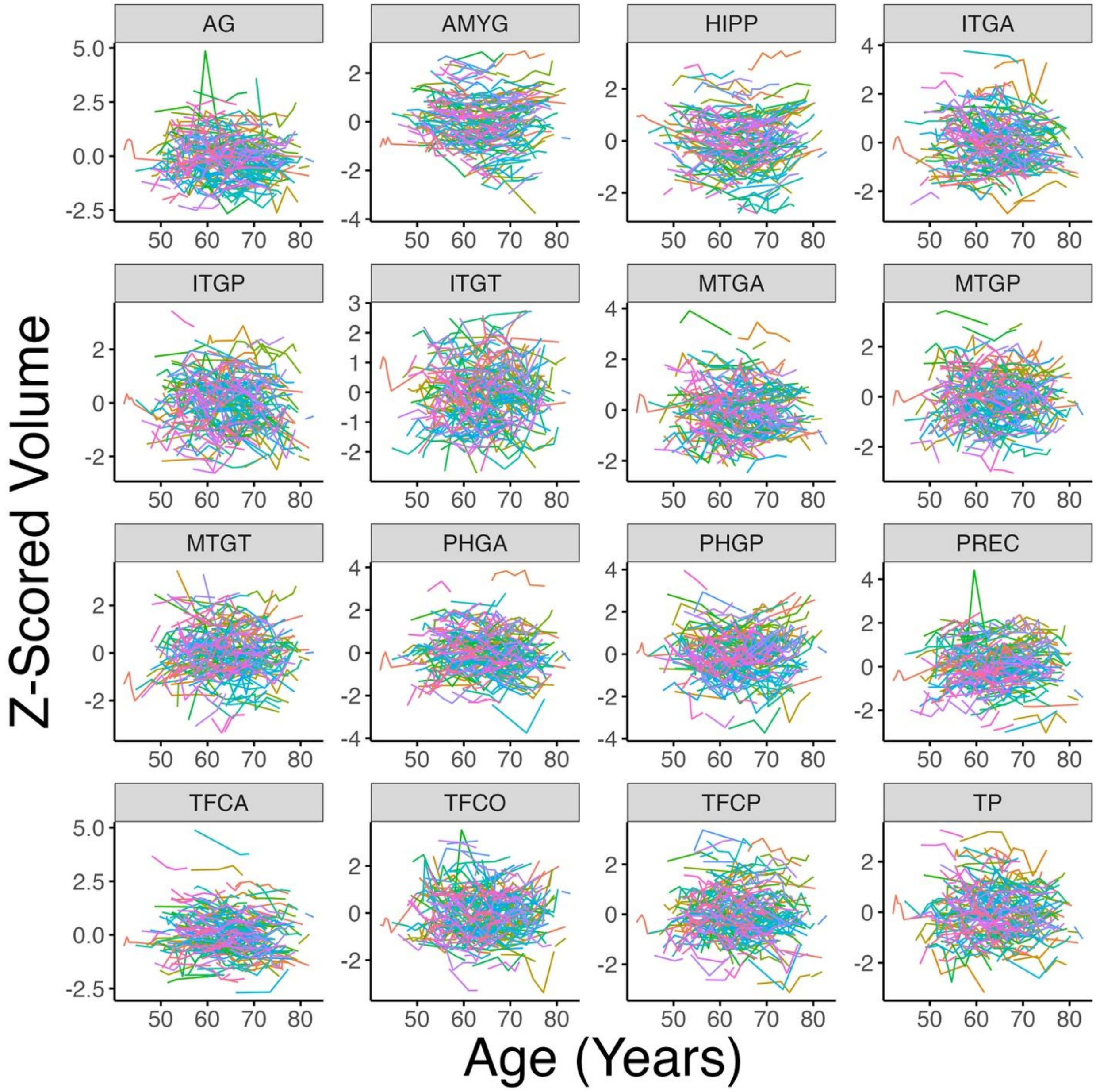


© 2025 Stephenson HG et al. *JAMA Network Open*.

## **eFigure 8.** Regional effects of A+

**A**: β estimates of interactions between A+ and time that survived FDR correction. **B**: Forest plot of simple slopes for time by group arranged by observed magnitude in A+ group. More negative numbers indicate worse effects of time. Points show simple slopes while lines show the lower and upper bounds of 95% confidence intervals. **C**: Interaction plots depicting the effects of time for A+ vs. A- groups.

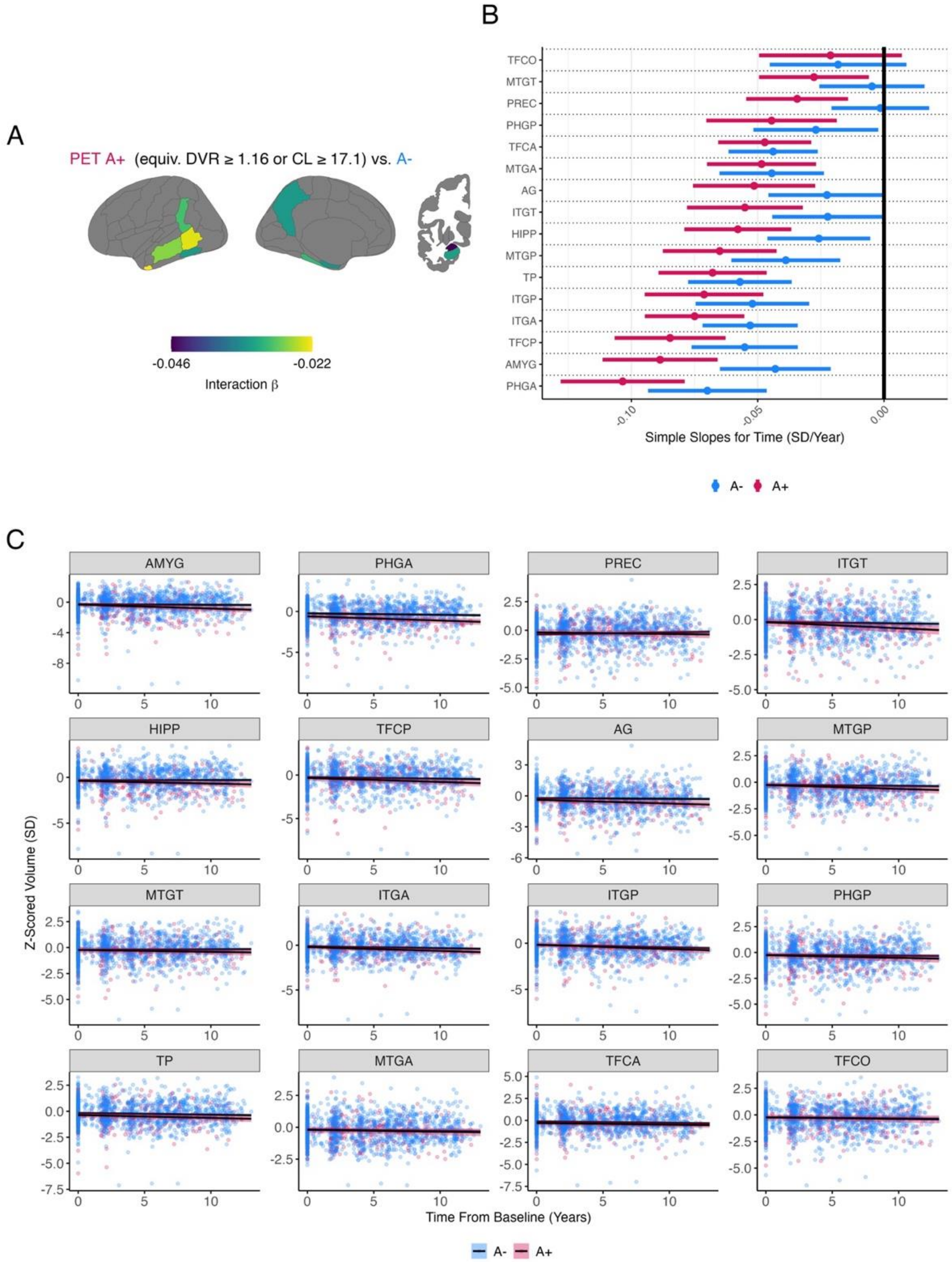


© 2025 Stephenson HG et al. *JAMA Network Open*.

## **eFigure 9.** Regional effects of T+

**A**: β estimates of interactions between T+ and time that survived FDR correction. **B**: Forest plot of simple slopes for time by group arranged by observed magnitude in T+ group. More negative numbers indicate worse effects of time. Points show simple slopes while lines show the lower and upper bounds of 95% confidence intervals. **C**: Interaction plots depicting the effects of time for T+ vs. T- groups.

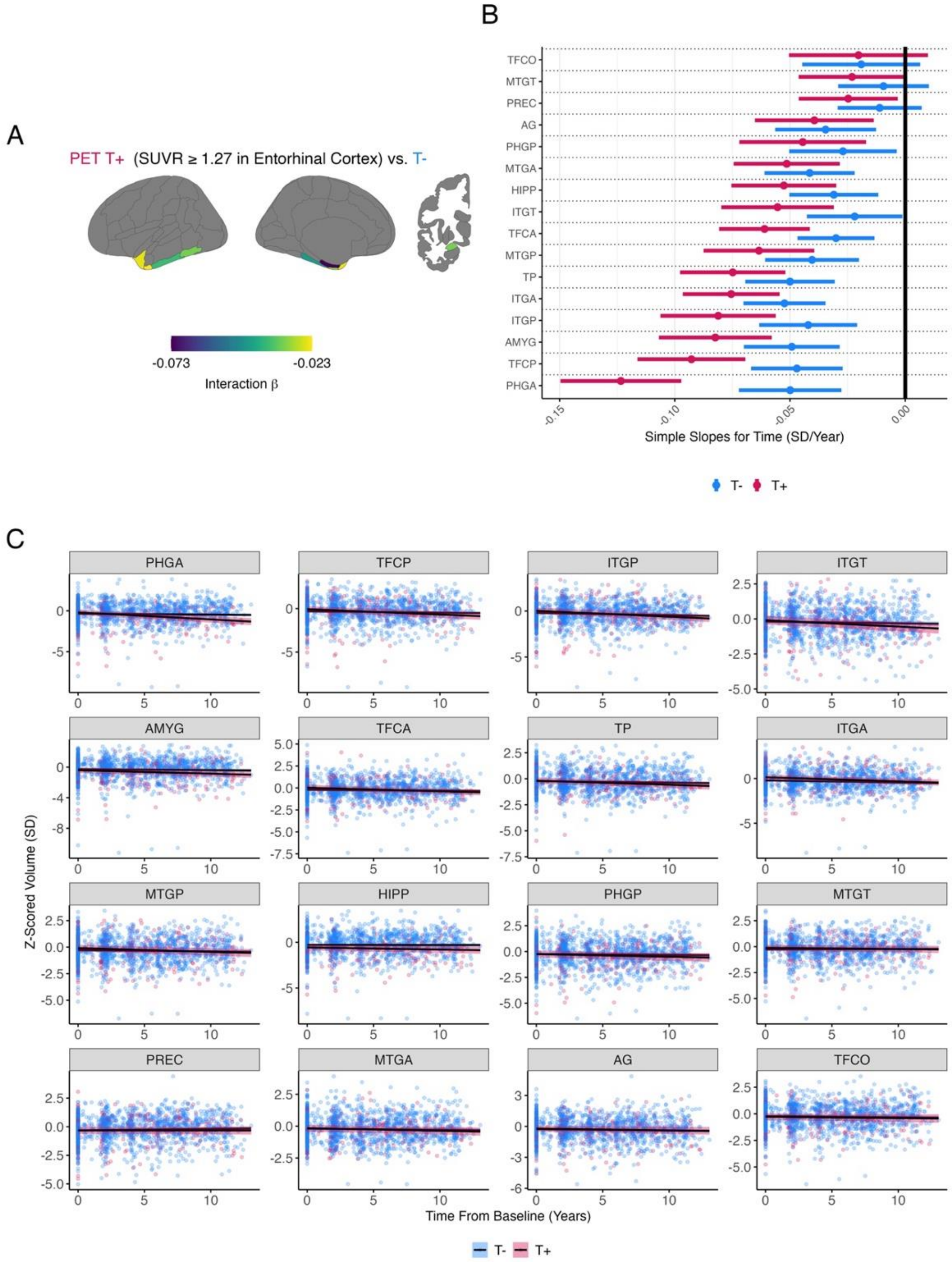


© 2025 Stephenson HG et al. *JAMA Network Open*.

**eTable 7.** Results for each interaction term (A+/- analysis)

Note that uncorrected and FDR-corrected *p*-values may seem identical due to rounding.

| **A+ vs. A- (Interaction)** | | | | | | |
|---|---|---|---|---|---|---|
| **ROI** | Slope | $CI_{lower}$ | $CI_{upper}$ | $p_{uncorr}$ | *pFDR* | Partial η2 |
| AG | -0.029 | -0.051 | -0.007 | 0.01 | 0.023 | 0.019 |
| ITGA | -0.022 | -0.04 | -0.004 | 0.018 | 0.031 | 0.018 |
| ITGP | -0.019 | -0.041 | 0.002 | 0.081 | 0.118 | 0.01 |
| ITGT | -0.033 | -0.054 | -0.012 | 0.002 | 0.009 | 0.029 |
| MTGA | -0.004 | -0.024 | 0.016 | 0.693 | 0.752 | 0 |
| MTGP | -0.026 | -0.047 | -0.005 | 0.014 | 0.027 | 0.018 |
| MTGT | -0.023 | -0.043 | -0.003 | 0.022 | 0.036 | 0.015 |
| PHGA | -0.034 | -0.056 | -0.011 | 0.004 | 0.012 | 0.023 |
| PHGP | -0.018 | -0.041 | 0.006 | 0.147 | 0.197 | 0.006 |
| PREC | -0.033 | -0.051 | -0.015 | $p < 0.001$ | 0.003 | 0.034 |
| TFCA | -0.003 | -0.02 | 0.014 | 0.705 | 0.752 | 0 |
| TFCP | -0.03 | -0.05 | -0.009 | 0.004 | 0.012 | 0.025 |
| TFCO | -0.003 | -0.029 | 0.023 | 0.82 | 0.82 | 0 |
| TP | -0.011 | -0.031 | 0.009 | 0.286 | 0.352 | 0.004 |
| HIPP | -0.032 | -0.052 | -0.012 | 0.002 | 0.009 | 0.025 |
| AMYG | -0.046 | -0.068 | -0.023 | $p < 0.001$ | 0.001 | 0.043 |
| **T+ vs. T- (Interaction)** | | | | | | |
| **ROI** | Slope | $CI_{lower}$ | $CI_{upper}$ | $p_{uncorr}$ | *pFDR* | Partial η2 |
| AG | -0.005 | -0.051 | -0.007 | 0.675 | 0.72 | 0.001 |
| ITGA | -0.023 | -0.04 | -0.004 | 0.017 | 0.039 | 0.019 |
| ITGP | -0.039 | -0.041 | 0.002 | 0.001 | 0.003 | 0.039 |
| ITGT | -0.033 | -0.054 | -0.012 | 0.003 | 0.009 | 0.03 |
| MTGA | -0.01 | -0.024 | 0.016 | 0.353 | 0.403 | 0.003 |
| MTGP | -0.023 | -0.047 | -0.005 | 0.037 | 0.067 | 0.014 |
| MTGT | -0.014 | -0.043 | -0.003 | 0.192 | 0.236 | 0.005 |
| PHGA | -0.073 | -0.056 | -0.011 | $p < 0.001$ | $p < 0.001$ | 0.1 |
| PHGP | -0.017 | -0.041 | 0.006 | 0.167 | 0.223 | 0.006 |
| PREC | -0.014 | -0.051 | -0.015 | 0.156 | 0.223 | 0.006 |
| TFCA | -0.031 | -0.02 | 0.014 | 0.001 | 0.003 | 0.034 |
| TFCP | -0.046 | -0.05 | -0.009 | $p < 0.001$ | $p < 0.001$ | 0.056 |
| TFCO | -0.001 | -0.029 | 0.023 | 0.932 | 0.932 | 0 |
| TP | -0.025 | -0.031 | 0.009 | 0.02 | 0.039 | 0.018 |
| HIPP | -0.022 | -0.052 | -0.012 | 0.051 | 0.082 | 0.011 |
| AMYG | -0.033 | -0.068 | -0.023 | 0.006 | 0.016 | 0.022 |
| **Impaired vs. Unimpaired (Interaction)** | | | | | | |
| **ROI** | Slope | $CI_{lower}$ | $CI_{upper}$ | $p_{uncorr}$ | *pFDR* | Partial η2 |
| AG | -0.032 | -0.051 | -0.007 | 0.118 | 0.171 | 0.005 |
| ITGA | -0.053 | -0.04 | -0.004 | 0.002 | 0.009 | 0.022 |
| ITGP | -0.026 | -0.041 | 0.002 | 0.188 | 0.232 | 0.004 |
| ITGT | -0.003 | -0.054 | -0.012 | 0.893 | 0.893 | 0 |

© 2025 Stephenson HG et al. *JAMA Network Open*.

| | | | | | | |
|---|---|---|---|---|---|---|
| MTGA | -0.066 | -0.024 | 0.016 | $p < 0.001$ | 0.003 | 0.028 |
| MTGP | -0.047 | -0.047 | -0.005 | 0.015 | 0.026 | 0.013 |
| MTGT | -0.013 | -0.043 | -0.003 | 0.482 | 0.551 | 0.001 |
| PHGA | -0.064 | -0.056 | -0.011 | 0.002 | 0.009 | 0.02 |
| PHGP | -0.029 | -0.041 | 0.006 | 0.183 | 0.232 | 0.004 |
| PREC | -0.034 | -0.051 | -0.015 | 0.049 | 0.079 | 0.007 |
| TFCA | -0.041 | -0.02 | 0.014 | 0.01 | 0.02 | 0.014 |
| TFCP | -0.053 | -0.05 | -0.009 | 0.005 | 0.013 | 0.017 |
| TFCO | -0.016 | -0.029 | 0.023 | 0.517 | 0.551 | 0.001 |
| TP | -0.072 | -0.031 | 0.009 | $p < 0.001$ | 0.002 | 0.034 |
| HIPP | -0.047 | -0.052 | -0.012 | 0.009 | 0.02 | 0.015 |
| AMYG | -0.056 | -0.068 | -0.023 | 0.004 | 0.011 | 0.02 |

© 2025 Stephenson HG et al. *JAMA Network Open*.

**eTable 8.** Results for each main effect (A+/- analysis)

Note that uncorrected and FDR-corrected *p*-values may seem identical due to rounding.

| A+ vs. A- (Main Effect) | | | | | | |
|---|---|---|---|---|---|---|
| **ROI** | Slope | $CI_{lower}$ | $CI_{upper}$ | $p_{uncorr}$ | *pFDR* | Partial η2 |
| AG | -0.166 | -0.483 | 0.15 | 0.304 | 0.973 | 0.003 |
| ITGA | -0.086 | -0.41 | 0.239 | 0.606 | 0.973 | 0.001 |
| ITGP | 0.006 | -0.348 | 0.36 | 0.973 | 0.973 | 0 |
| ITGT | -0.016 | -0.339 | 0.308 | 0.924 | 0.973 | 0 |
| MTGA | -0.025 | -0.316 | 0.266 | 0.866 | 0.973 | 0 |
| MTGP | -0.018 | -0.349 | 0.313 | 0.915 | 0.973 | 0 |
| MTGT | -0.009 | -0.337 | 0.318 | 0.955 | 0.973 | 0 |
| PHGA | -0.362 | -0.714 | -0.009 | 0.045 | 0.72 | 0.011 |
| PHGP | -0.032 | -0.378 | 0.313 | 0.854 | 0.973 | 0 |
| PREC | 0.189 | -0.128 | 0.507 | 0.243 | 0.973 | 0.004 |
| TFCA | -0.178 | -0.517 | 0.16 | 0.302 | 0.973 | 0.003 |
| TFCP | -0.074 | -0.445 | 0.296 | 0.695 | 0.973 | 0 |
| TFCO | -0.016 | -0.359 | 0.327 | 0.929 | 0.973 | 0 |
| TP | -0.198 | -0.513 | 0.116 | 0.216 | 0.973 | 0.004 |
| HIPP | -0.058 | -0.404 | 0.287 | 0.741 | 0.973 | 0 |
| AMYG | -0.026 | -0.403 | 0.351 | 0.892 | 0.973 | 0 |
| **T+ vs. T- (Main Effect)** | | | | | | |
| **ROI** | Slope | $CI_{lower}$ | $CI_{upper}$ | $p_{uncorr}$ | *pFDR* | Partial η2 |
| AG | 0.07 | -0.277 | 0.418 | 0.693 | 0.978 | 0 |
| ITGA | 0.351 | -0.005 | 0.707 | 0.054 | 0.799 | 0.01 |
| ITGP | 0.222 | -0.166 | 0.611 | 0.263 | 0.799 | 0.003 |
| ITGT | 0.097 | -0.258 | 0.452 | 0.594 | 0.95 | 0.001 |
| MTGA | 0.023 | -0.296 | 0.343 | 0.886 | 0.99 | 0 |
| MTGP | 0.192 | -0.17 | 0.555 | 0.3 | 0.799 | 0.003 |
| MTGT | 0.135 | -0.224 | 0.495 | 0.462 | 0.924 | 0.001 |
| PHGA | 0.151 | -0.235 | 0.538 | 0.443 | 0.924 | 0.002 |
| PHGP | -0.003 | -0.381 | 0.376 | 0.99 | 0.99 | 0 |
| PREC | -0.004 | -0.352 | 0.344 | 0.982 | 0.99 | 0 |
| TFCA | 0.236 | -0.136 | 0.608 | 0.214 | 0.799 | 0.004 |
| TFCP | 0.241 | -0.165 | 0.647 | 0.245 | 0.799 | 0.004 |
| TFCO | -0.063 | -0.439 | 0.314 | 0.744 | 0.978 | 0 |
| TP | 0.046 | -0.299 | 0.39 | 0.795 | 0.978 | 0 |
| HIPP | -0.281 | -0.66 | 0.098 | 0.148 | 0.799 | 0.006 |
| AMYG | -0.13 | -0.544 | 0.283 | 0.536 | 0.95 | 0.001 |
| **Impaired vs. Unimpaired (Main Effect)** | | | | | | |
| **ROI** | Slope | $CI_{lower}$ | $CI_{upper}$ | $p_{uncorr}$ | *pFDR* | Partial η2 |
| AG | -0.682 | -1.203 | -0.161 | 0.011 | 0.012 | 0.018 |
| ITGA | -1.114 | -1.647 | -0.582 | $p < 0.001$ | $p < 0.001$ | 0.044 |
| ITGP | -1.125 | -1.707 | -0.544 | $p < 0.001$ | $p < 0.001$ | 0.038 |
| ITGT | -0.993 | -1.525 | -0.461 | $p < 0.001$ | 0.001 | 0.035 |

© 2025 Stephenson HG et al. *JAMA Network Open*.

| | | | | | | |
|---|---|---|---|---|---|---|
| MTGA | -0.565 | -1.043 | -0.086 | 0.021 | 0.021 | 0.014 |
| MTGP | -0.793 | -1.336 | -0.251 | 0.004 | 0.006 | 0.022 |
| MTGT | -0.978 | -1.516 | -0.439 | $p < 0.001$ | 0.001 | 0.033 |
| PHGA | -1.24 | -1.818 | -0.662 | $p < 0.001$ | $p < 0.001$ | 0.046 |
| PHGP | -1.156 | -1.723 | -0.589 | $p < 0.001$ | $p < 0.001$ | 0.041 |
| PREC | -0.764 | -1.285 | -0.243 | 0.004 | 0.006 | 0.022 |
| TFCA | -0.809 | -1.366 | -0.253 | 0.005 | 0.006 | 0.022 |
| TFCP | -1.597 | -2.204 | -0.99 | $p < 0.001$ | $p < 0.001$ | 0.067 |
| TFCO | -1.111 | -1.675 | -0.547 | $p < 0.001$ | $p < 0.001$ | 0.039 |
| TP | -0.662 | -1.177 | -0.146 | 0.012 | 0.013 | 0.017 |
| HIPP | -1.279 | -1.845 | -0.712 | $p < 0.001$ | $p < 0.001$ | 0.051 |
| AMYG | -1.295 | -1.912 | -0.678 | $p < 0.001$ | $p < 0.001$ | 0.044 |

© 2025 Stephenson HG et al. *JAMA Network Open*.

**eFigure 10.** Regional effects of A+ duration

**A**: β estimates of interactions between that survived FDR correction. **B**: Interaction plots depicting the effects of A+ duration groups.

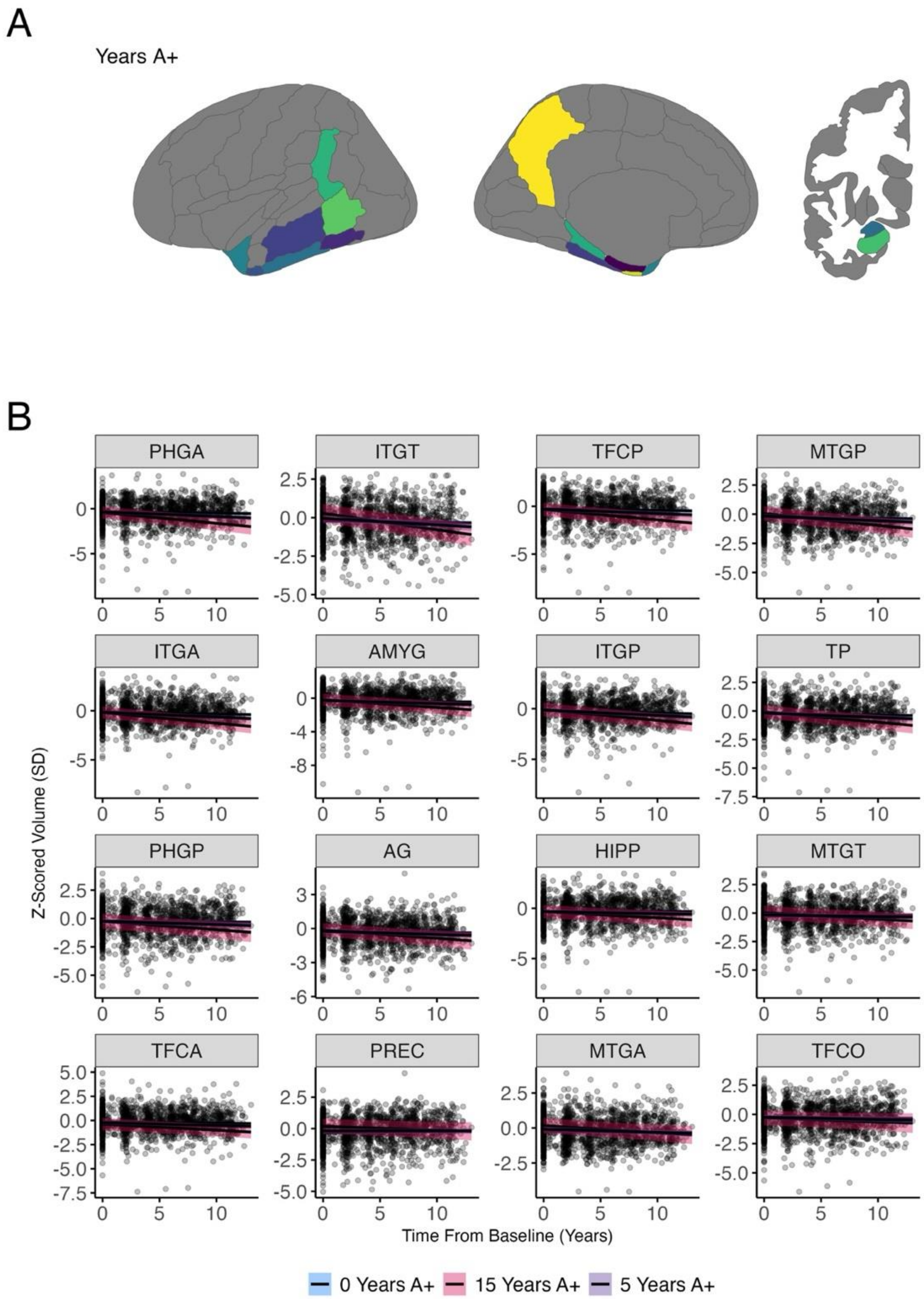


© 2025 Stephenson HG et al. *JAMA Network Open*.

## **eFigure 11.** Regional effects of T+ from the A+ duration analysis

**A**: β estimates of interactions between T+ and time that survived FDR correction. **B**: Forest plot of simple slopes for time by group arranged by observed magnitude in T+ group. More negative numbers indicate worse effects of time. Points show simple slopes while lines show the lower and upper bounds of 95% confidence intervals. **C**: Interaction plots depicting the effects of time for T+ vs. T- groups.

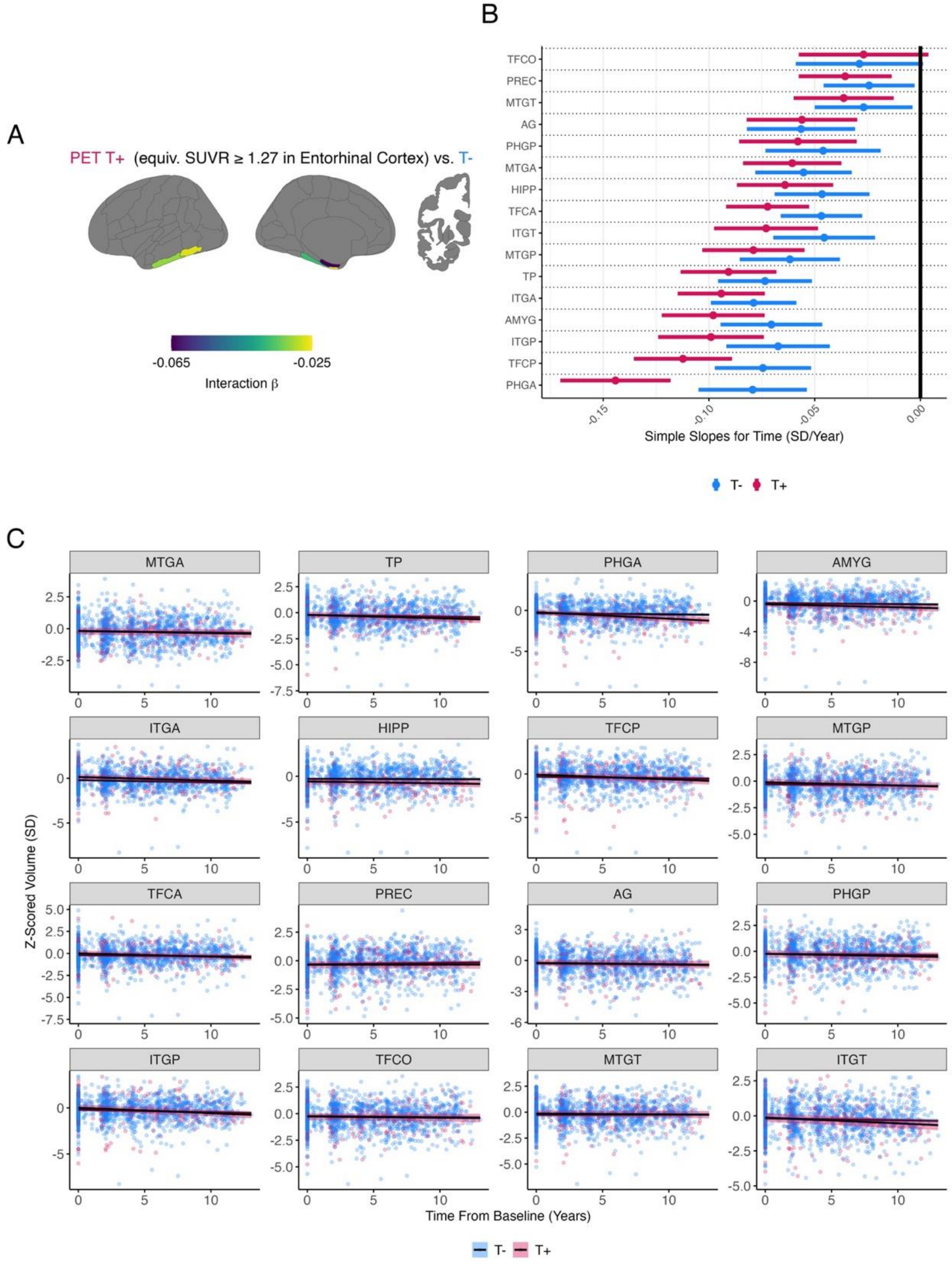


© 2025 Stephenson HG et al. *JAMA Network Open*.

**eTable 9.** Results for each interaction term (A+ duration analysis)

Note that uncorrected and FDR-corrected *p*-values may seem identical due to rounding.

| A+ Duration (Interaction) | | | | | | |
|---|---|---|---|---|---|---|
| **ROI** | Slope | $CI_{lower}$ | $CI_{upper}$ | $p_{uncorr}$ | *pFDR* | Partial η2 |
| AG | -0.004 | -0.008 | -0.001 | 0.006 | 0.009 | 0.019 |
| ITGA | -0.006 | -0.008 | -0.003 | $p < 0.001$ | $p < 0.001$ | 0.056 |
| ITGP | -0.005 | -0.008 | -0.002 | 0.001 | 0.001 | 0.035 |
| ITGT | -0.006 | -0.009 | -0.003 | $p < 0.001$ | $p < 0.001$ | 0.05 |
| MTGA | -0.003 | -0.006 | 0 | 0.055 | 0.059 | 0.011 |
| MTGP | -0.006 | -0.009 | -0.003 | $p < 0.001$ | $p < 0.001$ | 0.045 |
| MTGT | -0.004 | -0.007 | -0.001 | 0.006 | 0.009 | 0.02 |
| PHGA | -0.007 | -0.01 | -0.004 | $p < 0.001$ | $p < 0.001$ | 0.044 |
| PHGP | -0.005 | -0.008 | -0.001 | 0.009 | 0.011 | 0.018 |
| PREC | -0.003 | -0.006 | 0 | 0.023 | 0.027 | 0.013 |
| TFCA | -0.003 | -0.006 | -0.001 | 0.007 | 0.009 | 0.019 |
| TFCP | -0.006 | -0.009 | -0.003 | $p < 0.001$ | $p < 0.001$ | 0.048 |
| TFCO | -0.002 | -0.005 | 0.002 | 0.375 | 0.375 | 0.002 |
| TP | -0.005 | -0.008 | -0.002 | $p < 0.001$ | $p < 0.001$ | 0.037 |
| HIPP | -0.004 | -0.007 | -0.001 | 0.005 | 0.008 | 0.021 |
| AMYG | -0.005 | -0.009 | -0.002 | 0.001 | 0.001 | 0.03 |
| **T+ vs. T- (Interaction)** | | | | | | |
| **ROI** | Slope | $CI_{lower}$ | $CI_{upper}$ | $p_{uncorr}$ | *pFDR* | Partial η2 |
| AG | -0.005 | -0.008 | -0.001 | 0.694 | 0.793 | 0 |
| ITGA | -0.013 | -0.008 | -0.003 | 0.164 | 0.291 | 0.006 |
| ITGP | -0.029 | -0.008 | -0.002 | 0.01 | 0.037 | 0.023 |
| ITGT | -0.028 | -0.009 | -0.003 | 0.012 | 0.037 | 0.021 |
| MTGA | -0.002 | -0.006 | 0 | 0.878 | 0.878 | 0 |
| MTGP | -0.014 | -0.009 | -0.003 | 0.182 | 0.291 | 0.006 |
| MTGT | -0.011 | -0.007 | -0.001 | 0.268 | 0.371 | 0.004 |
| PHGA | -0.066 | -0.01 | -0.004 | $p < 0.001$ | $p < 0.001$ | 0.083 |
| PHGP | -0.01 | -0.008 | -0.001 | 0.42 | 0.517 | 0.002 |
| PREC | -0.021 | -0.006 | 0 | 0.031 | 0.065 | 0.014 |
| TFCA | -0.02 | -0.006 | -0.001 | 0.025 | 0.065 | 0.016 |
| TFCP | -0.039 | -0.009 | -0.003 | $p < 0.001$ | 0.002 | 0.042 |
| TFCO | 0.004 | -0.005 | 0.002 | 0.796 | 0.849 | 0 |
| TP | -0.011 | -0.008 | -0.002 | 0.278 | 0.371 | 0.004 |
| HIPP | -0.024 | -0.007 | -0.001 | 0.033 | 0.065 | 0.013 |
| AMYG | -0.038 | -0.009 | -0.002 | 0.002 | 0.009 | 0.029 |
| **Impaired vs. Unimpaired (Interaction)** | | | | | | |
| **ROI** | Slope | $CI_{lower}$ | $CI_{upper}$ | $p_{uncorr}$ | *pFDR* | Partial η2 |
| AG | -0.027 | -0.008 | -0.001 | 0.193 | 0.281 | 0.003 |
| ITGA | -0.043 | -0.008 | -0.003 | 0.011 | 0.049 | 0.015 |
| ITGP | -0.017 | -0.008 | -0.002 | 0.399 | 0.491 | 0.002 |
| ITGT | 0.007 | -0.009 | -0.003 | 0.726 | 0.726 | 0 |

© 2025 Stephenson HG et al. *JAMA Network Open*.

| | | | | | | |
|---|---|---|---|---|---|---|
| MTGA | -0.06 | -0.006 | 0 | 0.001 | 0.01 | 0.024 |
| MTGP | -0.037 | -0.009 | -0.003 | 0.056 | 0.099 | 0.008 |
| MTGT | -0.008 | -0.007 | -0.001 | 0.673 | 0.717 | 0 |
| PHGA | -0.052 | -0.01 | -0.004 | 0.013 | 0.049 | 0.014 |
| PHGP | -0.021 | -0.008 | -0.001 | 0.331 | 0.442 | 0.002 |
| PREC | -0.032 | -0.006 | 0 | 0.064 | 0.103 | 0.006 |
| TFCA | -0.034 | -0.006 | -0.001 | 0.033 | 0.066 | 0.01 |
| TFCP | -0.043 | -0.009 | -0.003 | 0.023 | 0.061 | 0.011 |
| TFCO | -0.012 | -0.005 | 0.002 | 0.615 | 0.703 | 0 |
| TP | -0.061 | -0.008 | -0.002 | 0.001 | 0.01 | 0.025 |
| HIPP | -0.04 | -0.007 | -0.001 | 0.028 | 0.064 | 0.011 |
| AMYG | -0.048 | -0.009 | -0.002 | 0.015 | 0.049 | 0.014 |

© 2025 Stephenson HG et al. *JAMA Network Open*.

**eTable 10.** Results for each main effect (A+ duration analysis)

Note that uncorrected and FDR-corrected *p*-values may seem identical due to rounding.

| A+ Duration (Main Effect) | | | | | | |
|---|---|---|---|---|---|---|
| **ROI** | Slope | $CI_{lower}$ | $CI_{upper}$ | $p_{uncorr}$ | *pFDR* | Partial η2 |
| AG | 0.011 | -0.032 | 0.053 | 0.628 | 0.987 | 0.001 |
| ITGA | -0.003 | -0.047 | 0.041 | 0.886 | 0.987 | 0 |
| ITGP | 0.005 | -0.043 | 0.053 | 0.829 | 0.987 | 0 |
| ITGT | 0.033 | -0.011 | 0.076 | 0.144 | 0.987 | 0.006 |
| MTGA | 0.025 | -0.014 | 0.065 | 0.209 | 0.987 | 0.004 |
| MTGP | 0.021 | -0.024 | 0.066 | 0.356 | 0.987 | 0.002 |
| MTGT | 0.018 | -0.026 | 0.062 | 0.421 | 0.987 | 0.002 |
| PHGA | -0.007 | -0.055 | 0.041 | 0.775 | 0.987 | 0 |
| PHGP | 0 | -0.047 | 0.046 | 0.987 | 0.987 | 0 |
| PREC | 0.036 | -0.007 | 0.079 | 0.099 | 0.987 | 0.007 |
| TFCA | -0.016 | -0.062 | 0.03 | 0.5 | 0.987 | 0.001 |
| TFCP | -0.005 | -0.055 | 0.045 | 0.847 | 0.987 | 0 |
| TFCO | -0.005 | -0.051 | 0.042 | 0.841 | 0.987 | 0 |
| TP | 0.01 | -0.033 | 0.053 | 0.644 | 0.987 | 0.001 |
| HIPP | 0.001 | -0.046 | 0.048 | 0.975 | 0.987 | 0 |
| AMYG | 0.009 | -0.042 | 0.06 | 0.735 | 0.987 | 0 |
| **T+ vs. T- (Main Effect)** | | | | | | |
| **ROI** | Slope | $CI_{lower}$ | $CI_{upper}$ | $p_{uncorr}$ | *pFDR* | Partial η2 |
| AG | -0.04 | -0.387 | 0.307 | 0.821 | 0.963 | 0 |
| ITGA | 0.322 | -0.033 | 0.678 | 0.077 | 0.895 | 0.009 |
| ITGP | 0.207 | -0.18 | 0.595 | 0.295 | 0.945 | 0.003 |
| ITGT | -0.018 | -0.372 | 0.335 | 0.919 | 0.963 | 0 |
| MTGA | -0.075 | -0.393 | 0.243 | 0.644 | 0.963 | 0.001 |
| MTGP | 0.113 | -0.248 | 0.475 | 0.539 | 0.963 | 0.001 |
| MTGT | 0.072 | -0.287 | 0.43 | 0.695 | 0.963 | 0 |
| PHGA | 0.009 | -0.379 | 0.397 | 0.963 | 0.963 | 0 |
| PHGP | -0.016 | -0.394 | 0.362 | 0.933 | 0.963 | 0 |
| PREC | -0.035 | -0.382 | 0.311 | 0.842 | 0.963 | 0 |
| TFCA | 0.204 | -0.167 | 0.575 | 0.283 | 0.945 | 0.003 |
| TFCP | 0.225 | -0.181 | 0.63 | 0.278 | 0.945 | 0.003 |
| TFCO | -0.054 | -0.43 | 0.321 | 0.777 | 0.963 | 0 |
| TP | -0.081 | -0.426 | 0.263 | 0.644 | 0.963 | 0.001 |
| HIPP | -0.308 | -0.686 | 0.071 | 0.112 | 0.895 | 0.007 |
| AMYG | -0.17 | -0.582 | 0.243 | 0.42 | 0.963 | 0.002 |
| **Impaired vs. Unimpaired (Main Effect)** | | | | | | |
| **ROI** | Slope | $CI_{lower}$ | $CI_{upper}$ | $p_{uncorr}$ | *pFDR* | Partial η2 |
| AG | -0.726 | -1.262 | -0.189 | 0.008 | 0.01 | 0.019 |
| ITGA | -1.106 | -1.655 | -0.557 | $p < 0.001$ | $p < 0.001$ | 0.041 |
| ITGP | -1.133 | -1.732 | -0.535 | $p < 0.001$ | $p < 0.001$ | 0.036 |
| ITGT | -1.086 | -1.632 | -0.54 | $p < 0.001$ | $p < 0.001$ | 0.04 |

© 2025 Stephenson HG et al. *JAMA Network Open*.

| | | | | | | |
|---|---|---|---|---|---|---|
| MTGA | -0.643 | -1.135 | -0.151 | 0.011 | 0.011 | 0.017 |
| MTGP | -0.852 | -1.41 | -0.294 | 0.003 | 0.004 | 0.024 |
| MTGT | -1.028 | -1.582 | -0.474 | $p < 0.001$ | 0.001 | 0.035 |
| PHGA | -1.251 | -1.849 | -0.653 | $p < 0.001$ | $p < 0.001$ | 0.044 |
| PHGP | -1.153 | -1.736 | -0.569 | $p < 0.001$ | $p < 0.001$ | 0.039 |
| PREC | -0.846 | -1.382 | -0.311 | 0.002 | 0.003 | 0.025 |
| TFCA | -0.777 | -1.35 | -0.204 | 0.008 | 0.01 | 0.019 |
| TFCP | -1.581 | -2.206 | -0.956 | $p < 0.001$ | $p < 0.001$ | 0.063 |
| TFCO | -1.096 | -1.677 | -0.515 | $p < 0.001$ | $p < 0.001$ | 0.036 |
| TP | -0.71 | -1.242 | -0.178 | 0.009 | 0.01 | 0.018 |
| HIPP | -1.284 | -1.867 | -0.7 | $p < 0.001$ | $p < 0.001$ | 0.048 |
| AMYG | -1.321 | -1.956 | -0.685 | $p < 0.001$ | $p < 0.001$ | 0.043 |

© 2025 Stephenson HG et al. *JAMA Network Open*.

**eFigure 12.** Regional effects of A+ with CU individuals only

**A**: β estimates of interactions between A+ and time that survived FDR correction. **B**: Forest plot of simple slopes for time by group arranged by observed magnitude in A+ group. More negative numbers indicate worse effects of time. Points show simple slopes while lines show the lower and upper bounds of 95% confidence intervals. **C**: Interaction plots depicting the effects of time for A+ vs. A- groups.

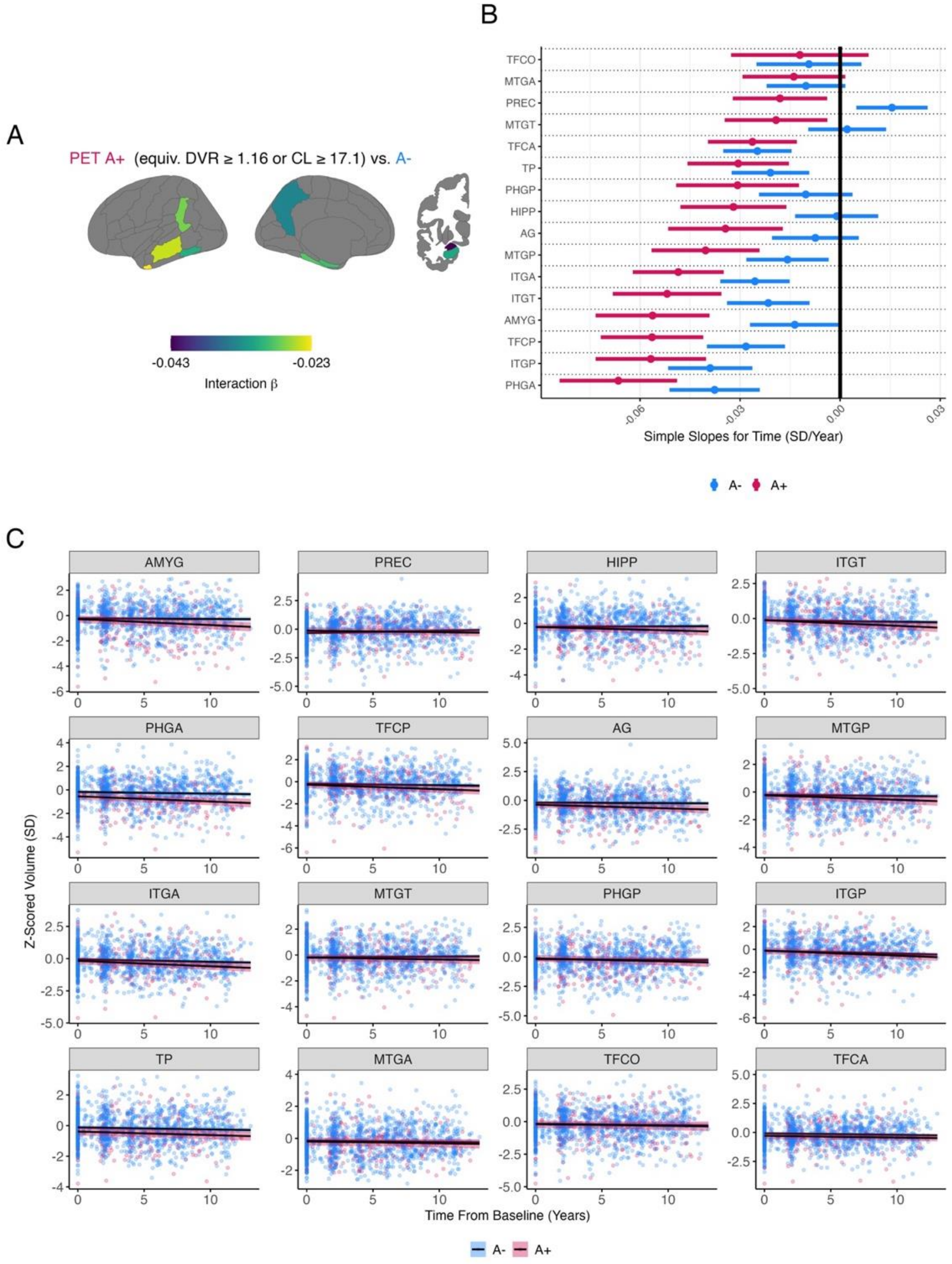


© 2025 Stephenson HG et al. *JAMA Network Open*.

**eFigure 13.** Regional effects of T+ with CU individuals only

**A**: β estimates of interactions between T+ and time that survived FDR correction. **B**: Forest plot of simple slopes for time by group arranged by observed magnitude in T+ group. More negative numbers indicate worse effects of time. Points show simple slopes while lines show the lower and upper bounds of 95% confidence intervals. **C**: Interaction plots depicting the effects of time for T+ vs. T- groups.

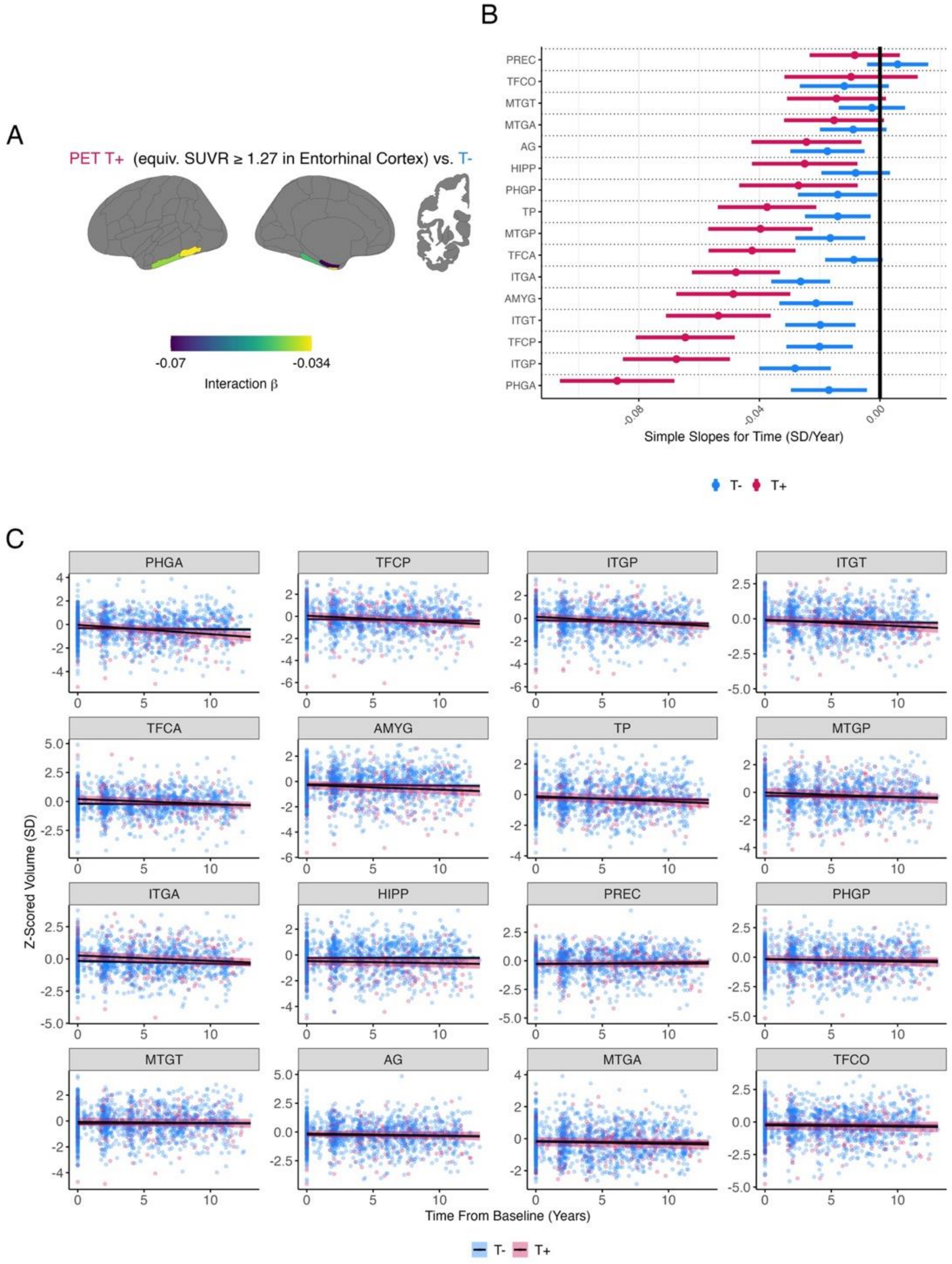


© 2025 Stephenson HG et al. *JAMA Network Open*.

**eTable 11.** Results for each interaction term (A+/- analysis) with CU individuals only

Note that uncorrected and FDR-corrected *p*-values may seem identical due to rounding.

| A+ vs. A- (Interaction) | | | | | | |
|---|---|---|---|---|---|---|
| **ROI** | Slope | $CI_{lower}$ | $CI_{upper}$ | $p_{uncorr}$ | *pFDR* | Partial η2 |
| AG | -0.027 | -0.049 | -0.005 | 0.019 | 0.038 | 0.016 |
| ITGA | -0.023 | -0.041 | -0.005 | 0.012 | 0.031 | 0.019 |
| ITGP | -0.018 | -0.039 | 0.004 | 0.107 | 0.142 | 0.008 |
| ITGT | -0.03 | -0.052 | -0.009 | 0.005 | 0.02 | 0.025 |
| MTGA | -0.004 | -0.024 | 0.016 | 0.724 | 0.827 | 0 |
| MTGP | -0.025 | -0.046 | -0.003 | 0.023 | 0.041 | 0.016 |
| MTGT | -0.021 | -0.041 | -0.001 | 0.038 | 0.06 | 0.012 |
| PHGA | -0.029 | -0.052 | -0.006 | 0.014 | 0.033 | 0.017 |
| PHGP | -0.02 | -0.044 | 0.004 | 0.096 | 0.139 | 0.008 |
| PREC | -0.034 | -0.052 | -0.015 | $p < 0.001$ | 0.003 | 0.035 |
| TFCA | -0.002 | -0.019 | 0.016 | 0.865 | 0.865 | 0 |
| TFCP | -0.028 | -0.048 | -0.008 | 0.006 | 0.02 | 0.023 |
| TFCO | -0.003 | -0.03 | 0.024 | 0.842 | 0.865 | 0 |
| TP | -0.01 | -0.029 | 0.01 | 0.34 | 0.418 | 0.003 |
| HIPP | -0.031 | -0.052 | -0.01 | 0.004 | 0.019 | 0.023 |
| AMYG | -0.043 | -0.065 | -0.02 | $p < 0.001$ | 0.003 | 0.038 |
| **T+ vs. T- (Interaction)** | | | | | | |
| **ROI** | Slope | $CI_{lower}$ | $CI_{upper}$ | $p_{uncorr}$ | *pFDR* | Partial η2 |
| AG | -0.007 | -0.049 | -0.005 | 0.558 | 0.596 | 0.001 |
| ITGA | -0.021 | -0.041 | -0.005 | 0.024 | 0.055 | 0.017 |
| ITGP | -0.039 | -0.039 | 0.004 | 0.001 | 0.003 | 0.04 |
| ITGT | -0.034 | -0.052 | -0.009 | 0.003 | 0.009 | 0.031 |
| MTGA | -0.006 | -0.024 | 0.016 | 0.553 | 0.596 | 0.001 |
| MTGP | -0.023 | -0.046 | -0.003 | 0.04 | 0.071 | 0.014 |
| MTGT | -0.012 | -0.041 | -0.001 | 0.271 | 0.361 | 0.004 |
| PHGA | -0.07 | -0.052 | -0.006 | $p < 0.001$ | $p < 0.001$ | 0.095 |
| PHGP | -0.013 | -0.044 | 0.004 | 0.308 | 0.379 | 0.003 |
| PREC | -0.014 | -0.052 | -0.015 | 0.146 | 0.212 | 0.006 |
| TFCA | -0.034 | -0.019 | 0.016 | $p < 0.001$ | 0.002 | 0.041 |
| TFCP | -0.045 | -0.048 | -0.008 | $p < 0.001$ | $p < 0.001$ | 0.055 |
| TFCO | 0.002 | -0.03 | 0.024 | 0.876 | 0.876 | 0 |
| TP | -0.023 | -0.029 | 0.01 | 0.027 | 0.055 | 0.017 |
| HIPP | -0.017 | -0.052 | -0.01 | 0.133 | 0.212 | 0.007 |
| AMYG | -0.027 | -0.065 | -0.02 | 0.024 | 0.055 | 0.015 |

© 2025 Stephenson HG et al. *JAMA Network Open*.

**eTable 12.** Results for each main effect (A+/- analysis) with CU individuals only

Note that uncorrected and FDR-corrected *p*-values may seem identical due to rounding.

| A+ vs. A- (Main Effect) | | | | | | |
|---|---|---|---|---|---|---|
| **ROI** | Slope | $CI_{lower}$ | $CI_{upper}$ | $p_{uncorr}$ | *pFDR* | Partial η2 |
| AG | -0.186 | -0.502 | 0.13 | 0.25 | 0.8 | 0.004 |
| ITGA | -0.116 | -0.438 | 0.206 | 0.48 | 0.985 | 0.001 |
| ITGP | 0.005 | -0.347 | 0.357 | 0.978 | 0.985 | 0 |
| ITGT | 0.019 | -0.305 | 0.343 | 0.909 | 0.985 | 0 |
| MTGA | -0.049 | -0.345 | 0.248 | 0.748 | 0.985 | 0 |
| MTGP | -0.029 | -0.361 | 0.304 | 0.865 | 0.985 | 0 |
| MTGT | -0.008 | -0.334 | 0.317 | 0.96 | 0.985 | 0 |
| PHGA | -0.376 | -0.703 | -0.048 | 0.025 | 0.402 | 0.014 |
| PHGP | 0.074 | -0.267 | 0.414 | 0.672 | 0.985 | 0.001 |
| PREC | 0.21 | -0.113 | 0.532 | 0.203 | 0.8 | 0.005 |
| TFCA | -0.214 | -0.542 | 0.113 | 0.201 | 0.8 | 0.005 |
| TFCP | -0.08 | -0.443 | 0.283 | 0.666 | 0.985 | 0.001 |
| TFCO | -0.024 | -0.369 | 0.322 | 0.893 | 0.985 | 0 |
| TP | -0.27 | -0.573 | 0.033 | 0.082 | 0.653 | 0.009 |
| HIPP | -0.003 | -0.335 | 0.329 | 0.985 | 0.985 | 0 |
| AMYG | -0.054 | -0.402 | 0.294 | 0.762 | 0.985 | 0 |
| **T+ vs. T- (Main Effect)** | | | | | | |
| **ROI** | Slope | $CI_{lower}$ | $CI_{upper}$ | $p_{uncorr}$ | *pFDR* | Partial η2 |
| AG | 0.112 | -0.237 | 0.46 | 0.531 | 0.845 | 0.001 |
| ITGA | 0.426 | 0.071 | 0.782 | 0.019 | 0.253 | 0.016 |
| ITGP | 0.3 | -0.088 | 0.689 | 0.131 | 0.421 | 0.007 |
| ITGT | 0.074 | -0.284 | 0.432 | 0.686 | 0.845 | 0 |
| MTGA | -0.033 | -0.36 | 0.295 | 0.845 | 0.845 | 0 |
| MTGP | 0.234 | -0.134 | 0.601 | 0.213 | 0.487 | 0.004 |
| MTGT | 0.142 | -0.218 | 0.502 | 0.44 | 0.845 | 0.002 |
| PHGA | 0.279 | -0.083 | 0.64 | 0.132 | 0.421 | 0.007 |
| PHGP | 0.038 | -0.338 | 0.414 | 0.842 | 0.845 | 0 |
| PREC | 0.036 | -0.32 | 0.392 | 0.842 | 0.845 | 0 |
| TFCA | 0.4 | 0.037 | 0.763 | 0.032 | 0.253 | 0.013 |
| TFCP | 0.31 | -0.091 | 0.711 | 0.13 | 0.421 | 0.007 |
| TFCO | -0.112 | -0.493 | 0.27 | 0.567 | 0.845 | 0.001 |
| TP | 0.093 | -0.241 | 0.428 | 0.585 | 0.845 | 0.001 |
| HIPP | -0.239 | -0.605 | 0.127 | 0.202 | 0.487 | 0.005 |
| AMYG | -0.049 | -0.433 | 0.335 | 0.802 | 0.845 | 0 |

© 2025 Stephenson HG et al. *JAMA Network Open*.

**eFigure 14.** Regional effects of A+ duration with CU individuals only

**A**: β estimates of interactions between that survived FDR correction. **B**: Interaction plots depicting the effects of A+ duration groups.

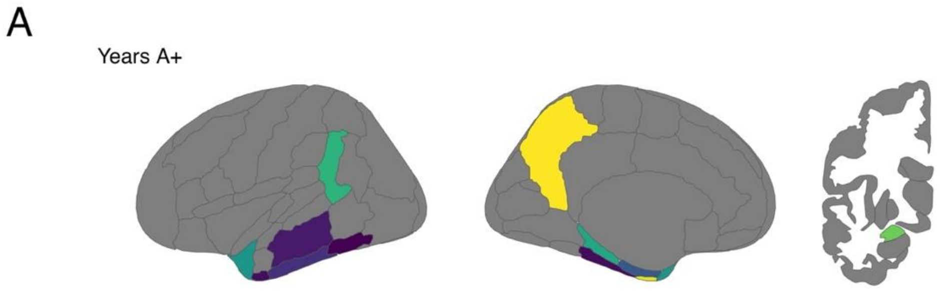


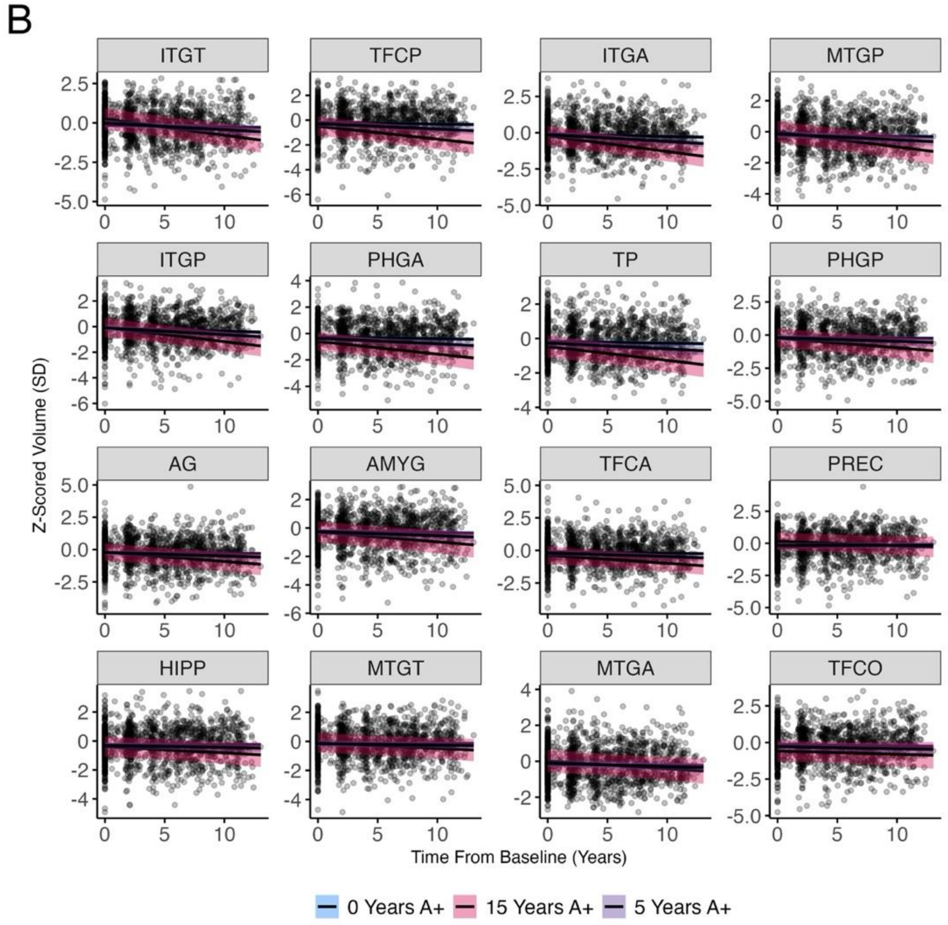


© 2025 Stephenson HG et al. *JAMA Network Open*.

**eFigure 15.** Regional effects of T+ from the A+ duration analysis with CU individuals only

**A**: β estimates of interactions between T+ and time that survived FDR correction. **B**: Forest plot of simple slopes for time by group arranged by observed magnitude in T+ group. More negative numbers indicate worse effects of time. Points show simple slopes while lines show the lower and upper bounds of 95% confidence intervals. **C**: Interaction plots depicting the effects of time for T+ vs. T- groups.

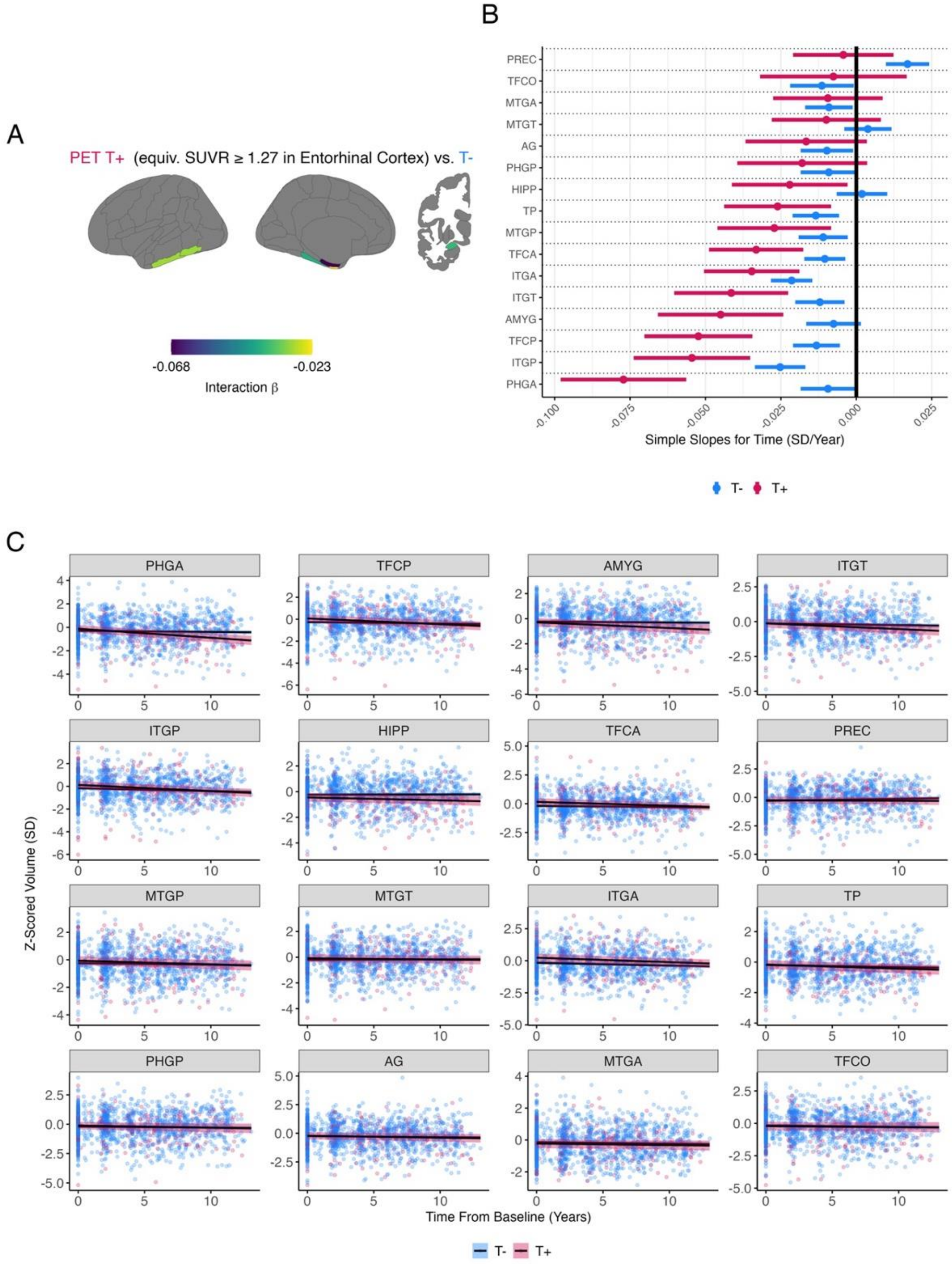

**eTable 13.** Results for each interaction term (A+ duration analysis) in CU individuals only

Note that uncorrected and FDR-corrected *p*-values may seem identical due to rounding.

| A+ Duration (Interaction) | | | | | | |
|---|---|---|---|---|---|---|
| **ROI** | Slope | $CI_{lower}$ | $CI_{upper}$ | $p_{uncorr}$ | *pFDR* | Partial η2 |
| AG | -0.004 | -0.008 | -0.001 | 0.011 | 0.019 | 0.019 |
| ITGA | -0.006 | -0.009 | -0.003 | $p < 0.001$ | $p < 0.001$ | 0.061 |
| ITGP | -0.006 | -0.009 | -0.003 | $p < 0.001$ | 0.001 | 0.04 |
| ITGT | -0.006 | -0.009 | -0.003 | $p < 0.001$ | 0.001 | 0.047 |
| MTGA | -0.002 | -0.005 | 0.001 | 0.124 | 0.132 | 0.008 |
| MTGP | -0.006 | -0.009 | -0.003 | $p < 0.001$ | 0.001 | 0.043 |
| MTGT | -0.003 | -0.006 | 0 | 0.064 | 0.075 | 0.01 |
| PHGA | -0.005 | -0.009 | -0.002 | 0.002 | 0.005 | 0.028 |
| PHGP | -0.004 | -0.008 | -0.001 | 0.014 | 0.022 | 0.018 |
| PREC | -0.003 | -0.006 | -0.001 | 0.018 | 0.026 | 0.016 |
| TFCA | -0.003 | -0.006 | -0.001 | 0.009 | 0.018 | 0.02 |
| TFCP | -0.006 | -0.009 | -0.003 | $p < 0.001$ | 0.001 | 0.048 |
| TFCO | -0.001 | -0.005 | 0.003 | 0.677 | 0.677 | 0 |
| TP | -0.005 | -0.008 | -0.002 | 0.002 | 0.005 | 0.031 |
| HIPP | -0.003 | -0.006 | 0 | 0.066 | 0.075 | 0.01 |
| AMYG | -0.004 | -0.007 | -0.001 | 0.022 | 0.029 | 0.015 |
| **T+ vs. T- (Interaction)** | | | | | | |
| **ROI** | Slope | $CI_{lower}$ | $CI_{upper}$ | $p_{uncorr}$ | *pFDR* | Partial η2 |
| AG | -0.007 | -0.008 | -0.001 | 0.555 | 0.634 | 0.001 |
| ITGA | -0.013 | -0.009 | -0.003 | 0.149 | 0.238 | 0.007 |
| ITGP | -0.029 | -0.009 | -0.003 | 0.009 | 0.029 | 0.024 |
| ITGT | -0.029 | -0.009 | -0.003 | 0.007 | 0.029 | 0.025 |
| MTGA | 0 | -0.005 | 0.001 | 0.973 | 0.973 | 0 |
| MTGP | -0.016 | -0.009 | -0.003 | 0.138 | 0.238 | 0.007 |
| MTGT | -0.014 | -0.006 | 0 | 0.188 | 0.274 | 0.005 |
| PHGA | -0.068 | -0.009 | -0.002 | $p < 0.001$ | $p < 0.001$ | 0.091 |
| PHGP | -0.009 | -0.008 | -0.001 | 0.477 | 0.588 | 0.002 |
| PREC | -0.021 | -0.006 | -0.001 | 0.028 | 0.063 | 0.015 |
| TFCA | -0.023 | -0.006 | -0.001 | 0.012 | 0.032 | 0.02 |
| TFCP | -0.039 | -0.009 | -0.003 | $p < 0.001$ | 0.001 | 0.044 |
| TFCO | 0.004 | -0.005 | 0.003 | 0.786 | 0.839 | 0 |
| TP | -0.013 | -0.008 | -0.002 | 0.218 | 0.29 | 0.005 |
| HIPP | -0.024 | -0.006 | 0 | 0.031 | 0.063 | 0.014 |
| AMYG | -0.037 | -0.007 | -0.001 | 0.002 | 0.01 | 0.029 |

**eTable 14.** Results for each main effect (A+ duration analysis) in CU individuals only

Note that uncorrected and FDR-corrected *p*-values may seem identical due to rounding.

| A+ Duration (Main Effect) | | | | | | |
|---|---|---|---|---|---|---|
| **ROI** | Slope | $CI_{lower}$ | $CI_{upper}$ | $p_{uncorr}$ | *pFDR* | Partial $\eta 2$ |
| AG | 0.002 | -0.045 | 0.05 | 0.926 | 0.926 | 0 |
| ITGA | -0.009 | -0.057 | 0.039 | 0.721 | 0.926 | 0 |
| ITGP | 0.003 | -0.049 | 0.056 | 0.899 | 0.926 | 0 |
| ITGT | 0.027 | -0.022 | 0.075 | 0.278 | 0.926 | 0.003 |
| MTGA | 0.012 | -0.033 | 0.056 | 0.611 | 0.926 | 0.001 |
| MTGP | 0.015 | -0.035 | 0.064 | 0.563 | 0.926 | 0.001 |
| MTGT | 0.006 | -0.042 | 0.055 | 0.799 | 0.926 | 0 |
| PHGA | -0.024 | -0.073 | 0.026 | 0.349 | 0.926 | 0.003 |
| PHGP | -0.003 | -0.054 | 0.048 | 0.898 | 0.926 | 0 |
| PREC | 0.032 | -0.017 | 0.08 | 0.2 | 0.926 | 0.005 |
| TFCA | -0.017 | -0.066 | 0.032 | 0.496 | 0.926 | 0.001 |
| TFCP | -0.02 | -0.075 | 0.034 | 0.464 | 0.926 | 0.002 |
| TFCO | -0.029 | -0.08 | 0.023 | 0.275 | 0.926 | 0.003 |
| TP | -0.02 | -0.065 | 0.026 | 0.395 | 0.926 | 0.002 |
| HIPP | -0.008 | -0.058 | 0.042 | 0.748 | 0.926 | 0 |
| AMYG | -0.003 | -0.055 | 0.049 | 0.915 | 0.926 | 0 |
| **T+ vs. T- (Main Effect)** | | | | | | |
| **ROI** | Slope | $CI_{lower}$ | $CI_{upper}$ | $p_{uncorr}$ | *pFDR* | Partial $\eta 2$ |
| AG | 0.024 | -0.322 | 0.37 | 0.892 | 0.951 | 0 |
| ITGA | 0.398 | 0.046 | 0.749 | 0.027 | 0.439 | 0.014 |
| ITGP | 0.29 | -0.094 | 0.674 | 0.139 | 0.557 | 0.006 |
| ITGT | 0.009 | -0.344 | 0.361 | 0.962 | 0.962 | 0 |
| MTGA | -0.088 | -0.411 | 0.236 | 0.595 | 0.951 | 0.001 |
| MTGP | 0.179 | -0.183 | 0.542 | 0.333 | 0.764 | 0.003 |
| MTGT | 0.123 | -0.232 | 0.479 | 0.497 | 0.951 | 0.001 |
| PHGA | 0.177 | -0.182 | 0.536 | 0.334 | 0.764 | 0.003 |
| PHGP | 0.079 | -0.293 | 0.45 | 0.679 | 0.951 | 0 |
| PREC | 0.045 | -0.306 | 0.397 | 0.8 | 0.951 | 0 |
| TFCA | 0.346 | -0.013 | 0.705 | 0.06 | 0.478 | 0.01 |
| TFCP | 0.33 | -0.066 | 0.726 | 0.104 | 0.554 | 0.008 |
| TFCO | -0.044 | -0.42 | 0.333 | 0.819 | 0.951 | 0 |
| TP | 0.025 | -0.307 | 0.356 | 0.884 | 0.951 | 0 |
| HIPP | -0.215 | -0.577 | 0.147 | 0.245 | 0.764 | 0.004 |
| AMYG | -0.063 | -0.442 | 0.317 | 0.746 | 0.951 | 0 |

© 2025 Stephenson HG et al. *JAMA Network Open*.

## **eFigure 16.** Regional effects of A+ with baseline volumes subtracted

**A**: β estimates of interactions between A+ and time that survived FDR correction. **B**: Forest plot of simple slopes for time by group arranged by observed magnitude in A+ group. More negative numbers indicate worse effects of time. Points show simple slopes while lines show the lower and upper bounds of 95% confidence intervals. **C**: Interaction plots depicting the effects of time for A+ vs. A- groups.

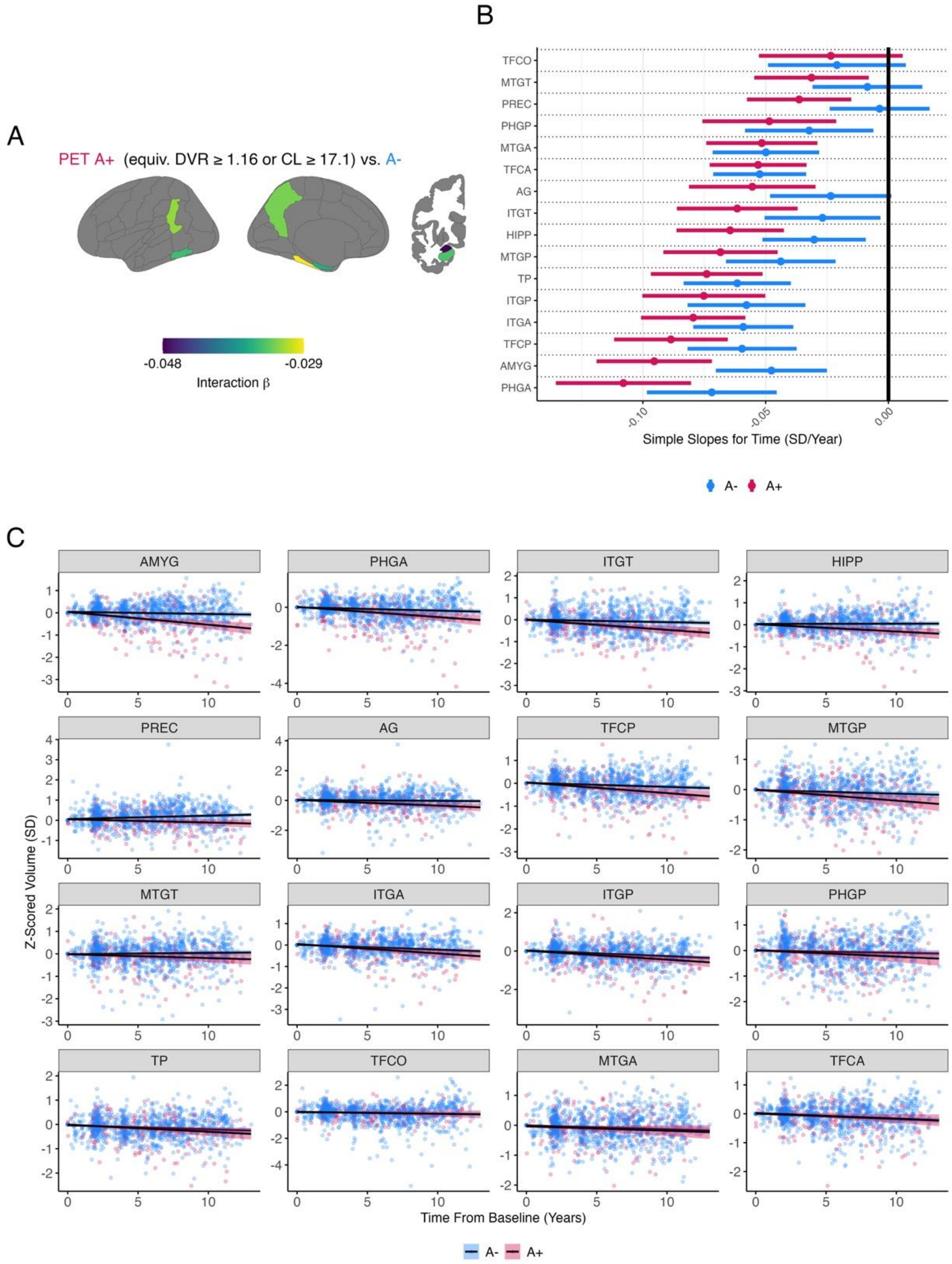


© 2025 Stephenson HG et al. *JAMA Network Open*.

**eFigure 17.** Regional effects of T+ with baseline volumes subtracted

**A**: β estimates of interactions between T+ and time that survived FDR correction. **B**: Forest plot of simple slopes for time by group arranged by observed magnitude in T+ group. More negative numbers indicate worse effects of time. Points show simple slopes while lines show the lower and upper bounds of 95% confidence intervals. **C**: Interaction plots depicting the effects of time for T+ vs. T- groups.

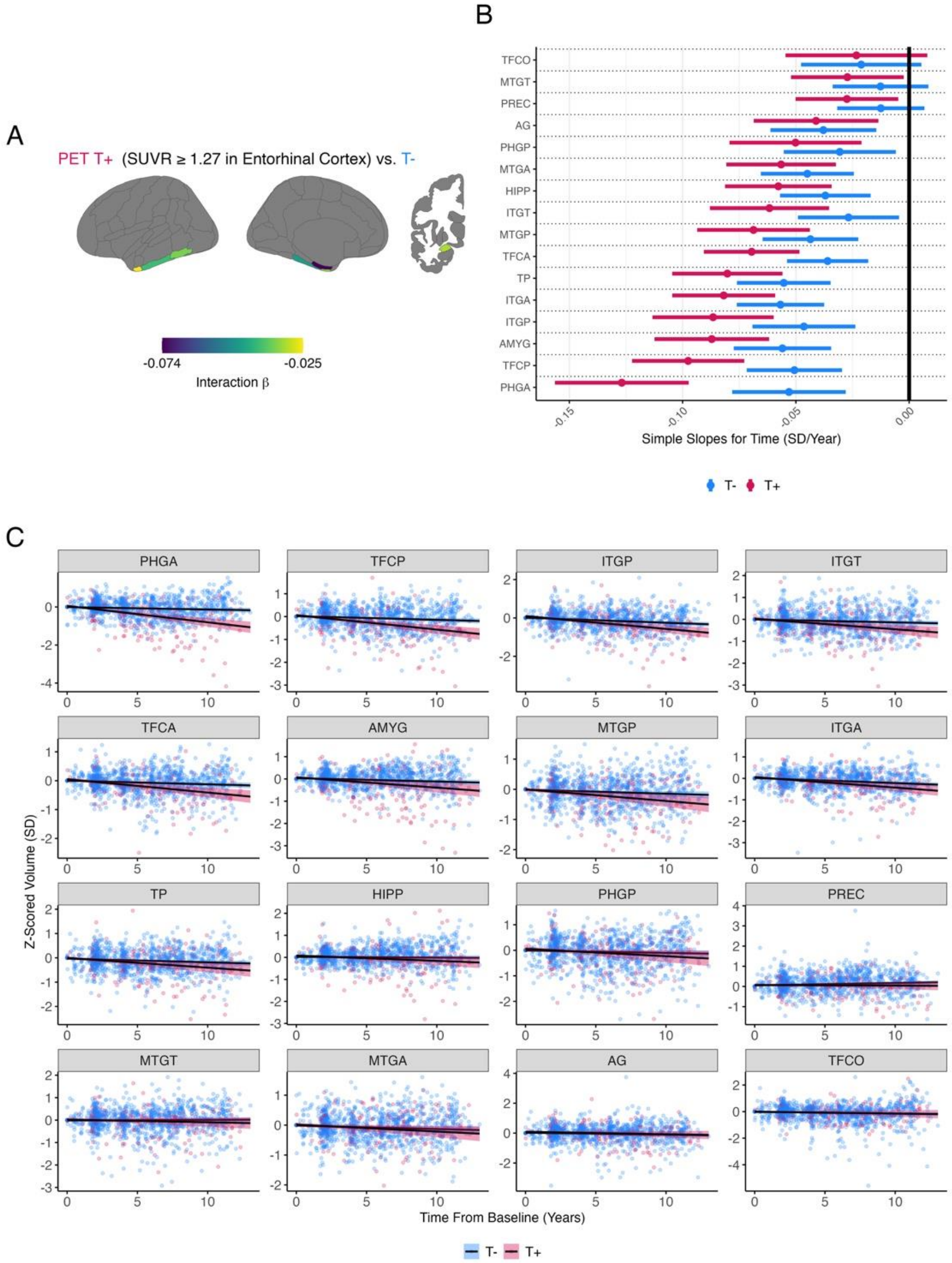


© 2025 Stephenson HG et al. *JAMA Network Open*.

**eTable 15.** Results for each interaction term (A+/- analysis) with baseline volumes subtracted

Note that uncorrected and FDR-corrected *p*-values may seem identical due to rounding.

| A+ vs. A- (Interaction) | | | | | | |
|---|---|---|---|---|---|---|
| **ROI** | Slope | $CI_{lower}$ | $CI_{upper}$ | $p_{uncorr}$ | *pFDR* | Partial η2 |
| AG | -0.032 | -0.056 | -0.008 | 0.009 | 0.021 | 0.018 |
| ITGA | -0.02 | -0.04 | 0 | 0.046 | 0.074 | 0.012 |
| ITGP | -0.017 | -0.041 | 0.006 | 0.146 | 0.212 | 0.006 |
| ITGT | -0.035 | -0.058 | -0.012 | 0.003 | 0.013 | 0.025 |
| MTGA | -0.002 | -0.023 | 0.02 | 0.878 | 0.936 | 0 |
| MTGP | -0.025 | -0.046 | -0.003 | 0.027 | 0.055 | 0.013 |
| MTGT | -0.023 | -0.044 | -0.001 | 0.039 | 0.069 | 0.011 |
| PHGA | -0.036 | -0.062 | -0.01 | 0.008 | 0.021 | 0.023 |
| PHGP | -0.016 | -0.042 | 0.009 | 0.212 | 0.283 | 0.004 |
| PREC | -0.033 | -0.052 | -0.013 | 0.001 | 0.009 | 0.029 |
| TFCA | -0.001 | -0.019 | 0.018 | 0.947 | 0.947 | 0 |
| TFCP | -0.029 | -0.051 | -0.007 | 0.009 | 0.021 | 0.019 |
| TFCO | -0.002 | -0.03 | 0.025 | 0.858 | 0.936 | 0 |
| TP | -0.012 | -0.034 | 0.009 | 0.256 | 0.315 | 0.004 |
| HIPP | -0.034 | -0.055 | -0.013 | 0.002 | 0.009 | 0.027 |
| AMYG | -0.048 | -0.071 | -0.025 | $p < 0.001$ | 0.001 | 0.044 |
| **T+ vs. T- (Interaction)** | | | | | | |
| **ROI** | Slope | $CI_{lower}$ | $CI_{upper}$ | $p_{uncorr}$ | *pFDR* | Partial η2 |
| AG | -0.003 | -0.056 | -0.008 | 0.799 | 0.853 | 0 |
| ITGA | -0.025 | -0.04 | 0 | 0.02 | 0.046 | 0.017 |
| ITGP | -0.04 | -0.041 | 0.006 | 0.002 | 0.006 | 0.031 |
| ITGT | -0.035 | -0.058 | -0.012 | 0.005 | 0.016 | 0.025 |
| MTGA | -0.012 | -0.023 | 0.02 | 0.31 | 0.354 | 0.003 |
| MTGP | -0.025 | -0.046 | -0.003 | 0.032 | 0.056 | 0.014 |
| MTGT | -0.015 | -0.044 | -0.001 | 0.205 | 0.253 | 0.004 |
| PHGA | -0.074 | -0.062 | -0.01 | $p < 0.001$ | $p < 0.001$ | 0.085 |
| PHGP | -0.02 | -0.042 | 0.009 | 0.154 | 0.205 | 0.006 |
| PREC | -0.015 | -0.052 | -0.013 | 0.151 | 0.205 | 0.006 |
| TFCA | -0.034 | -0.019 | 0.018 | 0.001 | 0.005 | 0.034 |
| TFCP | -0.047 | -0.051 | -0.007 | $p < 0.001$ | 0.001 | 0.048 |
| TFCO | -0.002 | -0.03 | 0.025 | 0.885 | 0.885 | 0 |
| TP | -0.025 | -0.034 | 0.009 | 0.031 | 0.056 | 0.015 |
| HIPP | -0.021 | -0.055 | -0.013 | 0.074 | 0.118 | 0.01 |
| AMYG | -0.031 | -0.071 | -0.025 | 0.013 | 0.035 | 0.018 |
| **Impaired vs. Unimpaired (Interaction)** | | | | | | |
| **ROI** | Slope | $CI_{lower}$ | $CI_{upper}$ | $p_{uncorr}$ | *pFDR* | Partial η2 |
| AG | -0.036 | -0.056 | -0.008 | 0.1 | 0.132 | 0.005 |
| ITGA | -0.061 | -0.04 | 0 | 0.001 | 0.004 | 0.026 |
| ITGP | -0.034 | -0.041 | 0.006 | 0.107 | 0.132 | 0.006 |

| | | | | | | |
|---|---|---|---|---|---|---|
| ITGT | -0.01 | -0.058 | -0.012 | 0.64 | 0.64 | 0 |
| MTGA | -0.073 | -0.023 | 0.02 | $p$ < 0.001 | 0.001 | 0.033 |
| MTGP | -0.053 | -0.046 | -0.003 | 0.008 | 0.014 | 0.015 |
| MTGT | -0.021 | -0.044 | -0.001 | 0.296 | 0.339 | 0.002 |
| PHGA | -0.069 | -0.062 | -0.01 | 0.003 | 0.007 | 0.023 |
| PHGP | -0.037 | -0.042 | 0.009 | 0.107 | 0.132 | 0.006 |
| PREC | -0.034 | -0.052 | -0.013 | 0.058 | 0.093 | 0.007 |
| TFCA | -0.051 | -0.019 | 0.018 | 0.002 | 0.007 | 0.021 |
| TFCP | -0.059 | -0.051 | -0.007 | 0.003 | 0.007 | 0.02 |
| TFCO | -0.018 | -0.03 | 0.025 | 0.458 | 0.489 | 0.001 |
| TP | -0.082 | -0.034 | 0.009 | $p$ < 0.001 | $p$ < 0.001 | 0.041 |
| HIPP | -0.054 | -0.055 | -0.013 | 0.004 | 0.007 | 0.021 |
| AMYG | -0.062 | -0.071 | -0.025 | 0.002 | 0.007 | 0.023 |

© 2025 Stephenson HG et al. *JAMA Network Open*.

**eTable 16.** Results for each main effect (A+/- analysis) with baseline volumes subtracted

Note that uncorrected and FDR-corrected *p*-values may seem identical due to rounding.

| A+ vs. A- (Main Effect) | | | | | | |
|---|---|---|---|---|---|---|
| **ROI** | Slope | $CI_{lower}$ | $CI_{upper}$ | $p_{uncorr}$ | *pFDR* | Partial η2 |
| AG | -0.002 | -0.085 | 0.08 | 0.959 | 0.959 | 0 |
| ITGA | 0.031 | -0.028 | 0.09 | 0.301 | 0.959 | 0.001 |
| ITGP | -0.003 | -0.075 | 0.07 | 0.944 | 0.959 | 0 |
| ITGT | -0.005 | -0.079 | 0.069 | 0.899 | 0.959 | 0 |
| MTGA | -0.029 | -0.094 | 0.036 | 0.384 | 0.959 | 0.001 |
| MTGP | 0.012 | -0.056 | 0.081 | 0.724 | 0.959 | 0 |
| MTGT | -0.003 | -0.08 | 0.074 | 0.935 | 0.959 | 0 |
| PHGA | 0.023 | -0.042 | 0.087 | 0.492 | 0.959 | 0 |
| PHGP | 0.013 | -0.067 | 0.092 | 0.754 | 0.959 | 0 |
| PREC | -0.011 | -0.083 | 0.061 | 0.759 | 0.959 | 0 |
| TFCA | 0.02 | -0.033 | 0.072 | 0.462 | 0.959 | 0.001 |
| TFCP | 0.017 | -0.049 | 0.082 | 0.62 | 0.959 | 0 |
| TFCO | 0.021 | -0.064 | 0.106 | 0.632 | 0.959 | 0 |
| TP | 0.026 | -0.038 | 0.09 | 0.424 | 0.959 | 0.001 |
| HIPP | -0.01 | -0.054 | 0.034 | 0.663 | 0.959 | 0 |
| AMYG | -0.007 | -0.05 | 0.036 | 0.756 | 0.959 | 0 |
| **T+ vs. T- (Main Effect)** | | | | | | |
| **ROI** | Slope | $CI_{lower}$ | $CI_{upper}$ | $p_{uncorr}$ | *pFDR* | Partial η2 |
| AG | 0.062 | -0.029 | 0.152 | 0.182 | 0.416 | 0.002 |
| ITGA | 0.041 | -0.024 | 0.106 | 0.214 | 0.428 | 0.002 |
| ITGP | 0.079 | 0 | 0.158 | 0.051 | 0.344 | 0.004 |
| ITGT | 0.034 | -0.047 | 0.115 | 0.408 | 0.502 | 0.001 |
| MTGA | 0.031 | -0.04 | 0.103 | 0.392 | 0.502 | 0.001 |
| MTGP | 0.01 | -0.065 | 0.086 | 0.789 | 0.789 | 0 |
| MTGT | 0.041 | -0.044 | 0.125 | 0.344 | 0.502 | 0.001 |
| PHGA | 0.064 | -0.007 | 0.134 | 0.078 | 0.344 | 0.003 |
| PHGP | 0.066 | -0.022 | 0.153 | 0.141 | 0.376 | 0.003 |
| PREC | 0.02 | -0.059 | 0.098 | 0.627 | 0.717 | 0 |
| TFCA | 0.05 | -0.007 | 0.108 | 0.086 | 0.344 | 0.003 |
| TFCP | 0.042 | -0.029 | 0.114 | 0.249 | 0.442 | 0.002 |
| TFCO | 0.02 | -0.073 | 0.113 | 0.678 | 0.724 | 0 |
| TP | 0.03 | -0.04 | 0.1 | 0.398 | 0.502 | 0.001 |
| HIPP | 0.058 | 0.01 | 0.106 | 0.018 | 0.295 | 0.006 |
| AMYG | 0.037 | -0.011 | 0.084 | 0.128 | 0.376 | 0.002 |
| **Impaired vs. Unimpaired (Main Effect)** | | | | | | |
| **ROI** | Slope | $CI_{lower}$ | $CI_{upper}$ | $p_{uncorr}$ | *pFDR* | Partial η2 |
| AG | -0.015 | -0.157 | 0.128 | 0.842 | 0.898 | 0 |
| ITGA | -0.098 | -0.2 | 0.005 | 0.061 | 0.196 | 0.004 |
| ITGP | -0.153 | -0.278 | -0.029 | 0.016 | 0.086 | 0.006 |

| | | | | | | |
|---|---|---|---|---|---|---|
| ITGT | -0.056 | -0.184 | 0.071 | 0.385 | 0.561 | 0.001 |
| MTGA | -0.001 | -0.114 | 0.112 | 0.982 | 0.982 | 0 |
| MTGP | -0.017 | -0.136 | 0.101 | 0.776 | 0.886 | 0 |
| MTGT | -0.023 | -0.155 | 0.11 | 0.738 | 0.886 | 0 |
| PHGA | -0.148 | -0.259 | -0.036 | 0.009 | 0.076 | 0.005 |
| PHGP | -0.095 | -0.232 | 0.043 | 0.178 | 0.356 | 0.002 |
| PREC | -0.07 | -0.194 | 0.054 | 0.267 | 0.447 | 0.001 |
| TFCA | -0.092 | -0.182 | -0.001 | 0.047 | 0.19 | 0.004 |
| TFCP | -0.177 | -0.29 | -0.064 | 0.002 | 0.035 | 0.01 |
| TFCO | -0.041 | -0.188 | 0.106 | 0.586 | 0.781 | 0 |
| TP | -0.061 | -0.17 | 0.049 | 0.28 | 0.447 | 0.001 |
| HIPP | -0.067 | -0.143 | 0.009 | 0.084 | 0.224 | 0.003 |
| AMYG | -0.061 | -0.136 | 0.014 | 0.109 | 0.248 | 0.002 |

© 2025 Stephenson HG et al. *JAMA Network Open*.

**eFigure 18.** Regional effects of A+ duration with baseline volumes subtracted

**A**: β estimates of interactions between that survived FDR correction. **B**: Interaction plots depicting the effects of A+ duration groups.

A

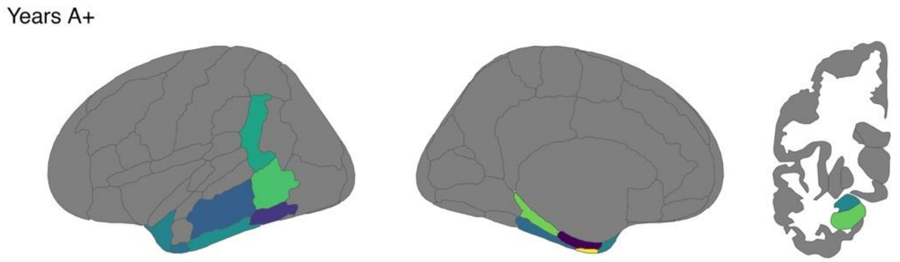


B

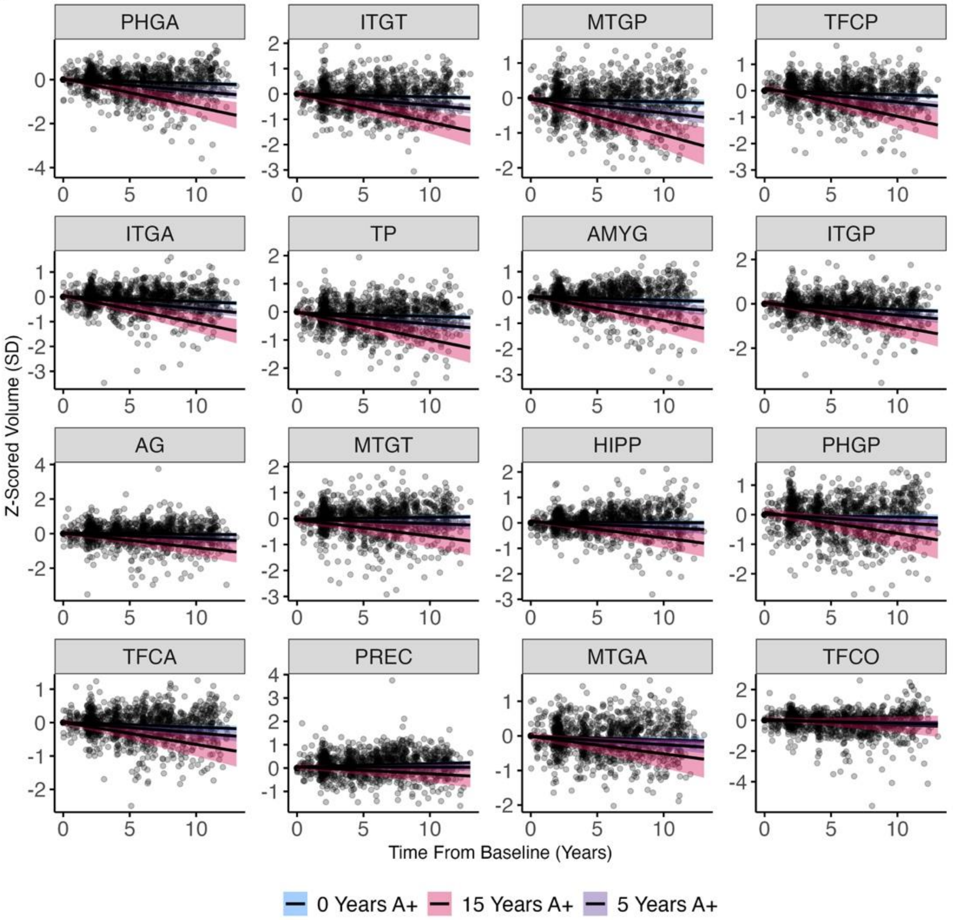


© 2025 Stephenson HG et al. *JAMA Network Open*.

## **eFigure 19.** Regional effects of T+ from the A+ duration analysis with baseline volumes subtracted

**A**: β estimates of interactions between T+ and time that survived FDR correction. **B**: Forest plot of simple slopes for time by group arranged by observed magnitude in T+ group. More negative numbers indicate worse effects of time. Points show simple slopes while lines show the lower and upper bounds of 95% confidence intervals. **C**: Interaction plots depicting the effects of time for T+ vs. T- groups.

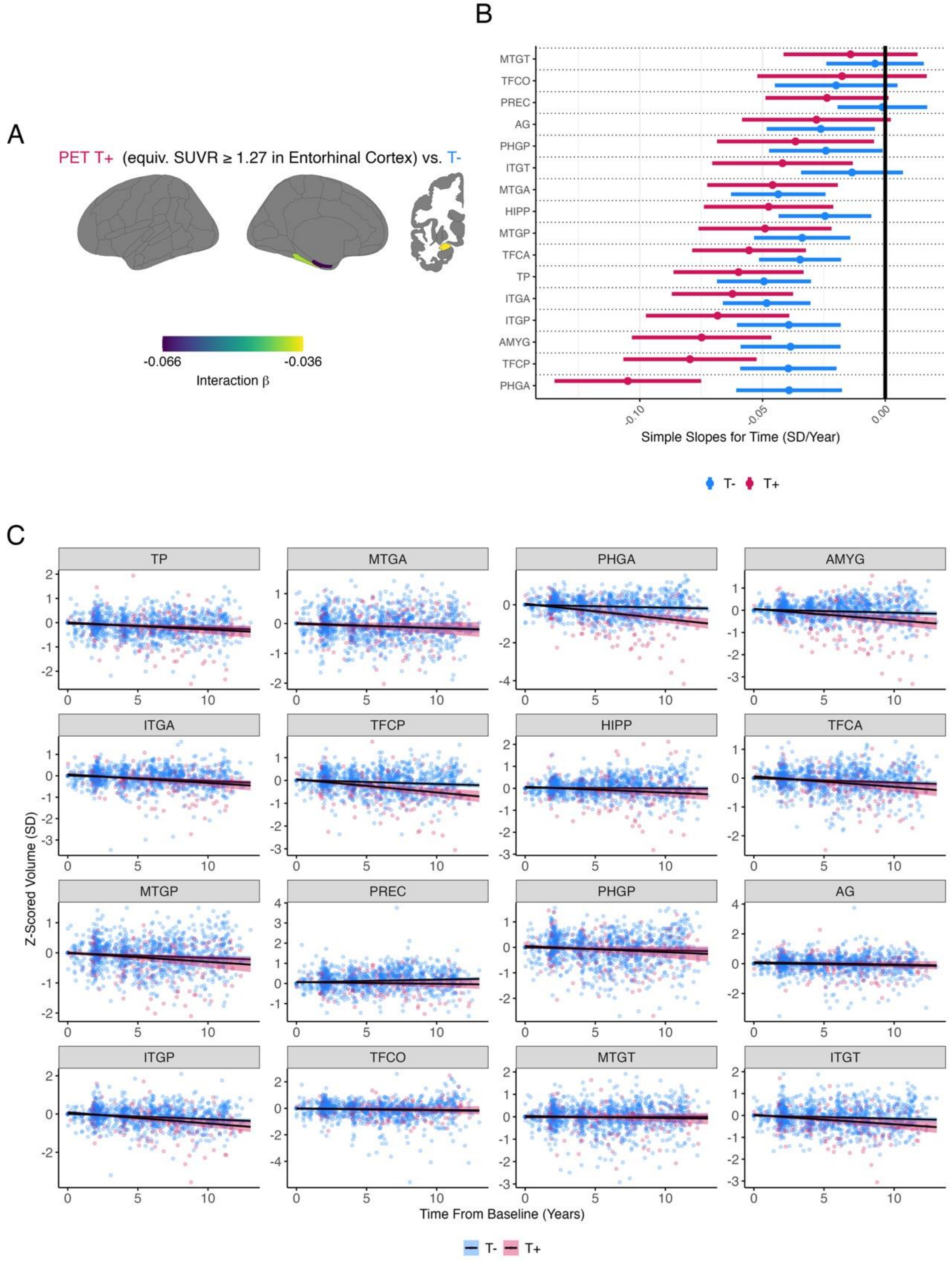


© 2025 Stephenson HG et al. *JAMA Network Open*.

**eTable 17.** Results for each interaction term (A+ duration analysis) with baseline volumes subtracted

Note that uncorrected and FDR-corrected *p*-values may seem identical due to rounding.

| **A+ Duration (Interaction)** | | | | | | |
|---|---|---|---|---|---|---|
| **ROI** | Slope | $CI_{lower}$ | $CI_{upper}$ | $p_{uncorr}$ | *pFDR* | Partial $\eta$2 |
| AG | -0.005 | -0.008 | -0.002 | 0.004 | 0.006 | 0.021 |
| ITGA | -0.006 | -0.009 | -0.003 | $p < 0.001$ | $p < 0.001$ | 0.045 |
| ITGP | -0.005 | -0.009 | -0.002 | 0.001 | 0.003 | 0.028 |
| ITGT | -0.007 | -0.01 | -0.004 | $p < 0.001$ | $p < 0.001$ | 0.045 |
| MTGA | -0.003 | -0.006 | 0 | 0.086 | 0.092 | 0.008 |
| MTGP | -0.006 | -0.009 | -0.003 | $p < 0.001$ | $p < 0.001$ | 0.04 |
| MTGT | -0.005 | -0.008 | -0.001 | 0.004 | 0.006 | 0.02 |
| PHGA | -0.007 | -0.011 | -0.004 | $p < 0.001$ | $p < 0.001$ | 0.048 |
| PHGP | -0.004 | -0.008 | -0.001 | 0.021 | 0.026 | 0.014 |
| PREC | -0.003 | -0.006 | 0 | 0.046 | 0.053 | 0.01 |
| TFCA | -0.003 | -0.006 | -0.001 | 0.011 | 0.015 | 0.018 |
| TFCP | -0.006 | -0.009 | -0.003 | $p < 0.001$ | $p < 0.001$ | 0.039 |
| TFCO | -0.002 | -0.005 | 0.002 | 0.432 | 0.432 | 0.001 |
| TP | -0.006 | -0.009 | -0.003 | $p < 0.001$ | 0.001 | 0.037 |
| HIPP | -0.004 | -0.007 | -0.001 | 0.005 | 0.007 | 0.021 |
| AMYG | -0.006 | -0.009 | -0.002 | 0.001 | 0.002 | 0.03 |
| **T+ vs. T- (Interaction)** | | | | | | |
| **ROI** | Slope | $CI_{lower}$ | $CI_{upper}$ | $p_{uncorr}$ | *pFDR* | Partial $\eta$2 |
| AG | -0.002 | -0.008 | -0.002 | 0.888 | 0.888 | 0 |
| ITGA | -0.014 | -0.009 | -0.003 | 0.183 | 0.296 | 0.006 |
| ITGP | -0.029 | -0.009 | -0.002 | 0.019 | 0.061 | 0.017 |
| ITGT | -0.028 | -0.01 | -0.004 | 0.019 | 0.061 | 0.017 |
| MTGA | -0.002 | -0.006 | 0 | 0.837 | 0.888 | 0 |
| MTGP | -0.015 | -0.009 | -0.003 | 0.185 | 0.296 | 0.005 |
| MTGT | -0.01 | -0.008 | -0.001 | 0.381 | 0.469 | 0.002 |
| PHGA | -0.066 | -0.011 | -0.004 | $p < 0.001$ | $p < 0.001$ | 0.076 |
| PHGP | -0.012 | -0.008 | -0.001 | 0.359 | 0.469 | 0.003 |
| PREC | -0.023 | -0.006 | 0 | 0.03 | 0.081 | 0.014 |
| TFCA | -0.021 | -0.006 | -0.001 | 0.035 | 0.081 | 0.014 |
| TFCP | -0.04 | -0.009 | -0.003 | 0.001 | 0.004 | 0.036 |
| TFCO | 0.002 | -0.005 | 0.002 | 0.868 | 0.888 | 0 |
| TP | -0.01 | -0.009 | -0.003 | 0.356 | 0.469 | 0.003 |
| HIPP | -0.023 | -0.007 | -0.001 | 0.046 | 0.093 | 0.012 |
| AMYG | -0.036 | -0.009 | -0.002 | 0.004 | 0.021 | 0.025 |
| **Impaired vs. Unimpaired (Interaction)** | | | | | | |
| **ROI** | Slope | $CI_{lower}$ | $CI_{upper}$ | $p_{uncorr}$ | *pFDR* | Partial $\eta$2 |
| AG | -0.029 | -0.008 | -0.002 | 0.2 | 0.283 | 0.003 |
| ITGA | -0.049 | -0.009 | -0.003 | 0.006 | 0.03 | 0.017 |
| ITGP | -0.023 | -0.009 | -0.002 | 0.277 | 0.342 | 0.003 |

| | | | | | | |
|---|---|---|---|---|---|---|
| ITGT | 0.002 | -0.01 | -0.004 | 0.916 | 0.916 | 0 |
| MTGA | -0.066 | -0.006 | 0 | 0.001 | 0.006 | 0.028 |
| MTGP | -0.041 | -0.009 | -0.003 | 0.04 | 0.07 | 0.009 |
| MTGT | -0.013 | -0.008 | -0.001 | 0.514 | 0.584 | 0.001 |
| PHGA | -0.055 | -0.011 | -0.004 | 0.011 | 0.03 | 0.015 |
| PHGP | -0.029 | -0.008 | -0.001 | 0.213 | 0.283 | 0.003 |
| PREC | -0.032 | -0.006 | 0 | 0.081 | 0.129 | 0.007 |
| TFCA | -0.043 | -0.006 | -0.001 | 0.012 | 0.03 | 0.015 |
| TFCP | -0.048 | -0.009 | -0.003 | 0.015 | 0.03 | 0.014 |
| TFCO | -0.015 | -0.005 | 0.002 | 0.547 | 0.584 | 0.001 |
| TP | -0.069 | -0.009 | -0.003 | $p < 0.001$ | 0.006 | 0.03 |
| HIPP | -0.047 | -0.007 | -0.001 | 0.013 | 0.03 | 0.015 |
| AMYG | -0.053 | -0.009 | -0.002 | 0.01 | 0.03 | 0.017 |

© 2025 Stephenson HG et al. *JAMA Network Open*.

**eTable 18.** Results for each main effect (A+ duration analysis) with baseline volumes subtracted

Note that uncorrected and FDR-corrected *p*-values may seem identical due to rounding.

| A+ Duration (Main Effect) | | | | | | |
|---|---|---|---|---|---|---|
| **ROI** | Slope | $CI_{lower}$ | $CI_{upper}$ | $p_{uncorr}$ | *pFDR* | Partial η2 |
| AG | -0.001 | -0.012 | 0.01 | 0.875 | 0.957 | 0 |
| ITGA | 0.002 | -0.006 | 0.01 | 0.656 | 0.957 | 0 |
| ITGP | 0.003 | -0.007 | 0.013 | 0.556 | 0.957 | 0 |
| ITGT | 0.001 | -0.009 | 0.011 | 0.818 | 0.957 | 0 |
| MTGA | 0 | -0.009 | 0.008 | 0.921 | 0.957 | 0 |
| MTGP | -0.001 | -0.01 | 0.009 | 0.86 | 0.957 | 0 |
| MTGT | -0.002 | -0.013 | 0.008 | 0.659 | 0.957 | 0 |
| PHGA | 0.003 | -0.006 | 0.012 | 0.488 | 0.957 | 0 |
| PHGP | 0.006 | -0.005 | 0.017 | 0.282 | 0.957 | 0.001 |
| PREC | 0 | -0.01 | 0.01 | 0.957 | 0.957 | 0 |
| TFCA | 0 | -0.007 | 0.007 | 0.931 | 0.957 | 0 |
| TFCP | 0.006 | -0.003 | 0.015 | 0.193 | 0.957 | 0.002 |
| TFCO | 0.006 | -0.006 | 0.017 | 0.326 | 0.957 | 0.001 |
| TP | 0.001 | -0.007 | 0.01 | 0.791 | 0.957 | 0 |
| HIPP | 0.003 | -0.003 | 0.009 | 0.274 | 0.957 | 0.001 |
| AMYG | 0.003 | -0.003 | 0.009 | 0.364 | 0.957 | 0.001 |
| **T+ vs. T- (Main Effect)** | | | | | | |
| **ROI** | Slope | $CI_{lower}$ | $CI_{upper}$ | $p_{uncorr}$ | *pFDR* | Partial η2 |
| AG | 0.066 | -0.025 | 0.156 | 0.156 | 0.416 | 0.003 |
| ITGA | 0.05 | -0.015 | 0.115 | 0.134 | 0.416 | 0.003 |
| ITGP | 0.068 | -0.012 | 0.147 | 0.094 | 0.377 | 0.003 |
| ITGT | 0.029 | -0.052 | 0.11 | 0.481 | 0.641 | 0.001 |
| MTGA | 0.019 | -0.053 | 0.091 | 0.605 | 0.693 | 0 |
| MTGP | 0.02 | -0.055 | 0.095 | 0.606 | 0.693 | 0 |
| MTGT | 0.049 | -0.036 | 0.133 | 0.259 | 0.482 | 0.002 |
| PHGA | 0.063 | -0.009 | 0.134 | 0.088 | 0.377 | 0.003 |
| PHGP | 0.051 | -0.036 | 0.139 | 0.25 | 0.482 | 0.002 |
| PREC | 0.018 | -0.061 | 0.097 | 0.649 | 0.693 | 0 |
| TFCA | 0.06 | 0.003 | 0.118 | 0.041 | 0.377 | 0.005 |
| TFCP | 0.03 | -0.041 | 0.102 | 0.407 | 0.593 | 0.001 |
| TFCO | 0.009 | -0.084 | 0.102 | 0.847 | 0.847 | 0 |
| TP | 0.038 | -0.032 | 0.108 | 0.288 | 0.482 | 0.001 |
| HIPP | 0.042 | -0.006 | 0.09 | 0.086 | 0.377 | 0.003 |
| AMYG | 0.025 | -0.022 | 0.072 | 0.301 | 0.482 | 0.001 |
| **Impaired vs. Unimpaired (Main Effect)** | | | | | | |
| **ROI** | Slope | $CI_{lower}$ | $CI_{upper}$ | $p_{uncorr}$ | *pFDR* | Partial η2 |
| AG | -0.006 | -0.151 | 0.14 | 0.939 | 0.968 | 0 |
| ITGA | -0.094 | -0.199 | 0.011 | 0.08 | 0.192 | 0.003 |
| ITGP | -0.158 | -0.286 | -0.03 | 0.015 | 0.117 | 0.006 |

© 2025 Stephenson HG et al. *JAMA Network Open*.

| | | | | | | |
|---|---|---|---|---|---|---|
| ITGT | -0.054 | -0.185 | 0.077 | 0.418 | 0.608 | 0.001 |
| MTGA | -0.002 | -0.118 | 0.113 | 0.968 | 0.968 | 0 |
| MTGP | -0.007 | -0.128 | 0.115 | 0.914 | 0.968 | 0 |
| MTGT | -0.01 | -0.146 | 0.125 | 0.882 | 0.968 | 0 |
| PHGA | -0.136 | -0.252 | -0.02 | 0.022 | 0.117 | 0.005 |
| PHGP | -0.107 | -0.249 | 0.034 | 0.137 | 0.273 | 0.003 |
| PREC | -0.066 | -0.193 | 0.061 | 0.307 | 0.523 | 0.001 |
| TFCA | -0.085 | -0.178 | 0.008 | 0.072 | 0.192 | 0.003 |
| TFCP | -0.187 | -0.303 | -0.072 | 0.002 | 0.025 | 0.011 |
| TFCO | -0.054 | -0.205 | 0.096 | 0.479 | 0.638 | 0.001 |
| TP | -0.056 | -0.169 | 0.056 | 0.327 | 0.523 | 0.001 |
| HIPP | -0.076 | -0.154 | 0.002 | 0.056 | 0.192 | 0.004 |
| AMYG | -0.068 | -0.145 | 0.009 | 0.084 | 0.192 | 0.003 |

© 2025 Stephenson HG et al. *JAMA Network Open*.

## **eTable 19.** Sample characteristics for the OASIS-3 dataset used for replication analysis

*P*-values taken from unpaired samples t-tests or chi-squared tests comparing A+/- groups, as appropriate for each variable and distribution. Age, sex, and ICV were added as covariates when comparing hippocampal volumes in these models. Age and sex were added as covariates when comparing entorhinal AV-1451 SUVR values. White vs. nonwhite was assigned based on each participants' self-reported primary race. Mean(SD) was used for normally-distributed variables while median[IQR] was used for non-normally-distributed variables.

| | Whole Sample | A- | A+ | *p*-value |
|---|---|---|---|---|
| N | 191 | 159 | 32 | NA |
| Number Female (%) | 113 | 90 | 23 | 0.16 |
| Number White (%) | 183 | 152 | 31 | 1 |
| Number APOE4+ (%) | 71 | 48 | 23 | $p < 0.001$ |
| Number Impaired (%) | 9 | 4 | 5 | 0.006 |
| Number T+ (%) | 31 | 15 | 16 | $p < 0.001$ |
| Baseline A+ Duration, median[IQR] | -9.23[1.65-8.09] | -10.60[-13.89--7.55] | 4.79[1.65-8.09] | NA |
| Aβ CL used for SILA, median[IQR] | 6.86[59.33-97.19] | 3.94[0.24-12.25] | 78.52[59.33-97.19] | NA |
| Age at Baseline MRI(Years), mean(SD) | 64.82(7.74) | 63.79(7.69) | 69.91(5.78) | $p < 0.001$ |
| Length of Follow-up (Years), median[IQR] | 6.11[3.54-8.96] | 6.13[3.46-9.02] | 5.88[3.84-8.54] | 0.74 |
| Number of Visits, median[IQR] | 3.00[2.00-4.00] | 3.00[2.00-5.00] | 3.00[2.00-4.00] | 0.66 |
| Entorhinal AV-1451 SUVR, median[IQR] | 1.02[0.89-1.19] | 0.98[0.88-1.12] | 1.30[1.04-1.80] | $p < 0.001$ |
| Baseline Hippocampal Volume (mL), median[IQR] | 3.47[3.21-3.70] | 3.50[3.25-3.71] | 3.33[3.17-3.47] | 0.88 |
| Aβ PET Age Difference/Baseline MRI (Years), median[IQR] | 5.98[3.41-8.72] | 6.05[3.41-8.76] | 5.88[3.29-8.13] | 0.33 |
| Tau PET Age Difference/Baseline MRI (Years), median[IQR] | 6.06[3.46-9.00] | 6.07[3.42-9.09] | 5.84[3.83-8.71] | 0.82 |
| Aβ PET Age Difference/Final MRI (Years), median[IQR] | 0.00[0.00-0.00] | 0.00[0.00-0.00] | 0.00[0.00-0.00] | 0.03 |
| Tau PET Age Difference/Final MRI (Years), median[IQR] | 0.00[-0.05-0.04] | 0.00[-0.05-0.03] | 0.00[-0.03-0.11] | 0.14 |
| Diagnosis/MRI Age Difference (Years), median[IQR] | 1.02[-0.17-1.77] | 0.97[-0.18-1.75] | 1.47[-0.05-1.82] | 0.17 |

© 2025 Stephenson HG et al. *JAMA Network Open*.

**eTable 20.** Results from initial z-scoring model in the OASIS-3 dataset

Bolded values signify significance at $p < 0.05$ (FDR-corrected).

| ROI | Unadjusted Volume (SD) | $\beta_{int}$ | $\beta_{sex}$ | $\beta_{ICV}$ | $\beta_{age}$ | RMSE |
|---|---|---|---|---|---|---|
| AG | 3.798(0.48) | **2.271** | -0.1 | **0.002** | **-0.026** | 0.331 |
| ITGA | 1.362(0.19) | **0.663** | 0.015 | **0.001** | **-0.007** | 0.139 |
| ITGP | 3.72(0.48) | **1.587** | -0.109 | **0.002** | **-0.021** | 0.331 |
| ITGT | 3.076(0.37) | **1.237** | -0.102 | **0.002** | **-0.016** | 0.229 |
| MTGA | 1.543(0.26) | 0.462 | -0.061 | **0.001** | **-0.011** | 0.186 |
| MTGP | 4.801(0.64) | **2.165** | -0.248 | **0.003** | **-0.035** | 0.398 |
| MTGT | 3.453(0.43) | **1.478** | -0.175 | **0.002** | **-0.02** | 0.28 |
| PHGA | 2.612(0.31) | **0.942** | 0.023 | **0.002** | **-0.011** | 0.181 |
| PHGP | 1.349(0.18) | **0.447** | 0.01 | **0.001** | **-0.006** | 0.114 |
| PREC | 9.068(1.1) | **4.555** | -0.393 | **0.006** | **-0.052** | 0.772 |
| TFCA | 1.467(0.17) | **0.434** | 0.046 | **0.001** | **-0.003** | 0.093 |
| TFCP | 3.517(0.37) | **1.455** | 0.06 | **0.002** | **-0.013** | 0.192 |
| TFCO | 2.927(0.32) | **0.829** | 0.027 | **0.002** | **-0.008** | 0.156 |
| TP | 7.368(1.13) | **4.711** | 0.143 | **0.004** | **-0.052** | 0.864 |
| HIPP | 3.486(0.38) | **1.812** | -0.006 | **0.002** | **-0.019** | 0.205 |
| AMYG | 1.709(0.19) | **0.665** | -0.023 | **0.001** | **-0.008** | 0.107 |

**eTable 21.** Results from final robust z-scoring model in the OASIS-3 dataset

Bolded values signify significance at $p < 0.05$ (FDR-corrected).

| ROI | Unadjusted Volume (SD) | $\beta_{int}$ | $\beta_{sex}$ | $\beta_{ICV}$ | $\beta_{age}$ | $\beta_{age\ x\ sex}$ | RMSE |
|---|---|---|---|---|---|---|---|
| AG | 3.881(0.452) | **2.281** | -0.087 | **0.002** | **-0.025** | | 0.284 |
| ITGA | 1.396(0.173) | **0.734** | 0.052 | **0.001** | **-0.007** | | 0.105 |
| ITGP | 3.812(0.451) | **1.758** | -0.047 | **0.002** | **-0.022** | | 0.258 |
| ITGT | 3.137(0.346) | **1.135** | -0.057 | **0.002** | **-0.014** | | 0.176 |
| MTGA | 1.589(0.238) | **0.721** | -0.008 | **0.001** | **-0.012** | | 0.148 |
| MTGP | 4.918(0.598) | **2.568** | -0.146 | **0.003** | **-0.036** | | 0.319 |
| MTGT | 3.526(0.398) | **1.507** | -0.117 | **0.002** | **-0.018** | | 0.231 |
| PHGA | 2.664(0.3) | **0.697** | 0.032 | **0.002** | **-0.009** | | 0.134 |
| PHGP | 1.379(0.176) | **0.334** | 0.025 | **0.001** | **-0.005** | | 0.098 |
| PREC | 9.254(1.042) | **4.449** | -0.404 | **0.005** | **-0.047** | | 0.685 |
| TFCA | 1.492(0.164) | **0.349** | **0.057** | **0.001** | **-0.003** | | 0.069 |
| TFCP | 3.568(0.374) | **1.179** | 0.082 | **0.002** | **-0.01** | | 0.164 |
| TFCO | 2.971(0.311) | **0.627** | 0.053 | **0.002** | **-0.005** | | 0.127 |
| TP | 7.578(1.06) | **5.814** | **-3.686** | **0.004** | **-0.073** | **0.059** | 0.682 |
| HIPP | 3.541(0.366) | **1.551** | 0.01 | **0.002** | **-0.016** | | 0.166 |
| AMYG | 1.737(0.189) | **0.445** | -0.022 | **0.001** | **-0.006** | | 0.081 |

© 2025 Stephenson HG et al. *JAMA Network Open*.

**eFigure 20.** Regional counts of N+ among A- individuals in the OASIS-3 dataset

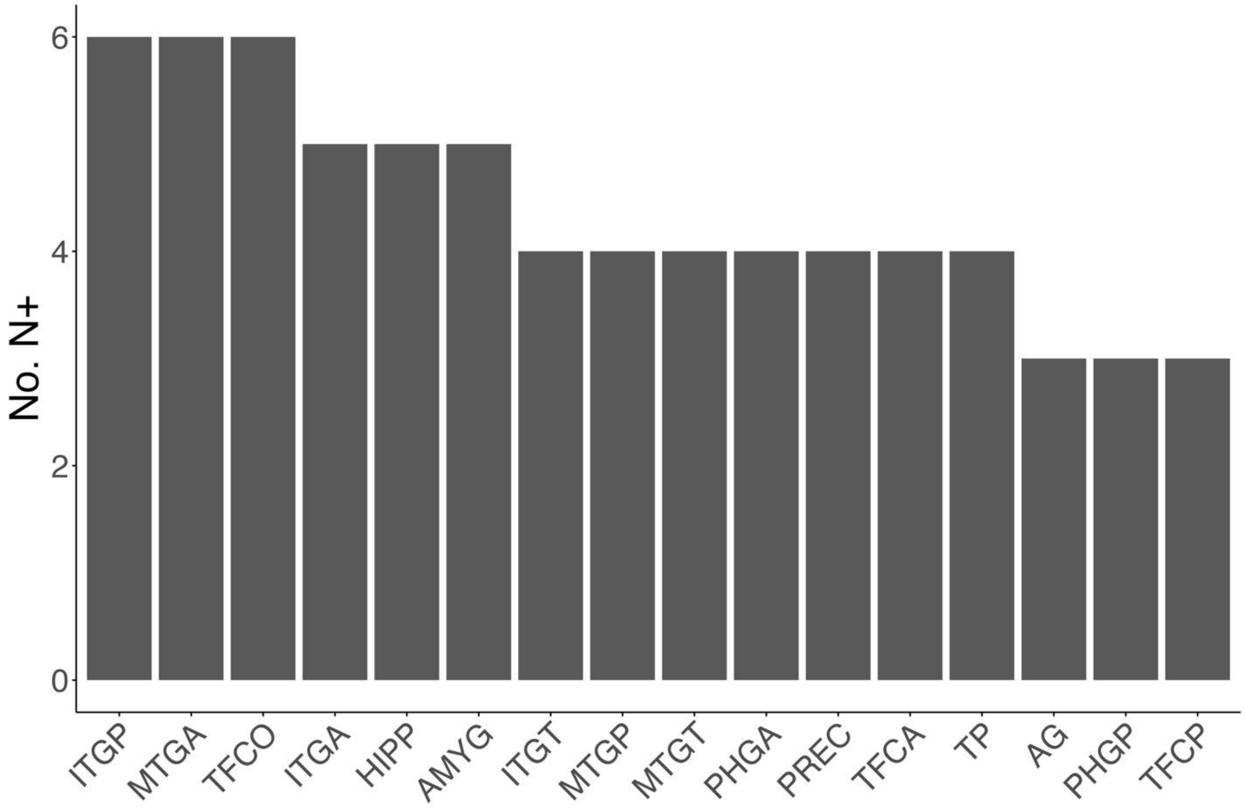


© 2025 Stephenson HG et al. *JAMA Network Open*.

**eFigure 21.** Unadjusted volumes in the final z-scoring sample in the OASIS-3 dataset

Separate lines and colors denote different participants.

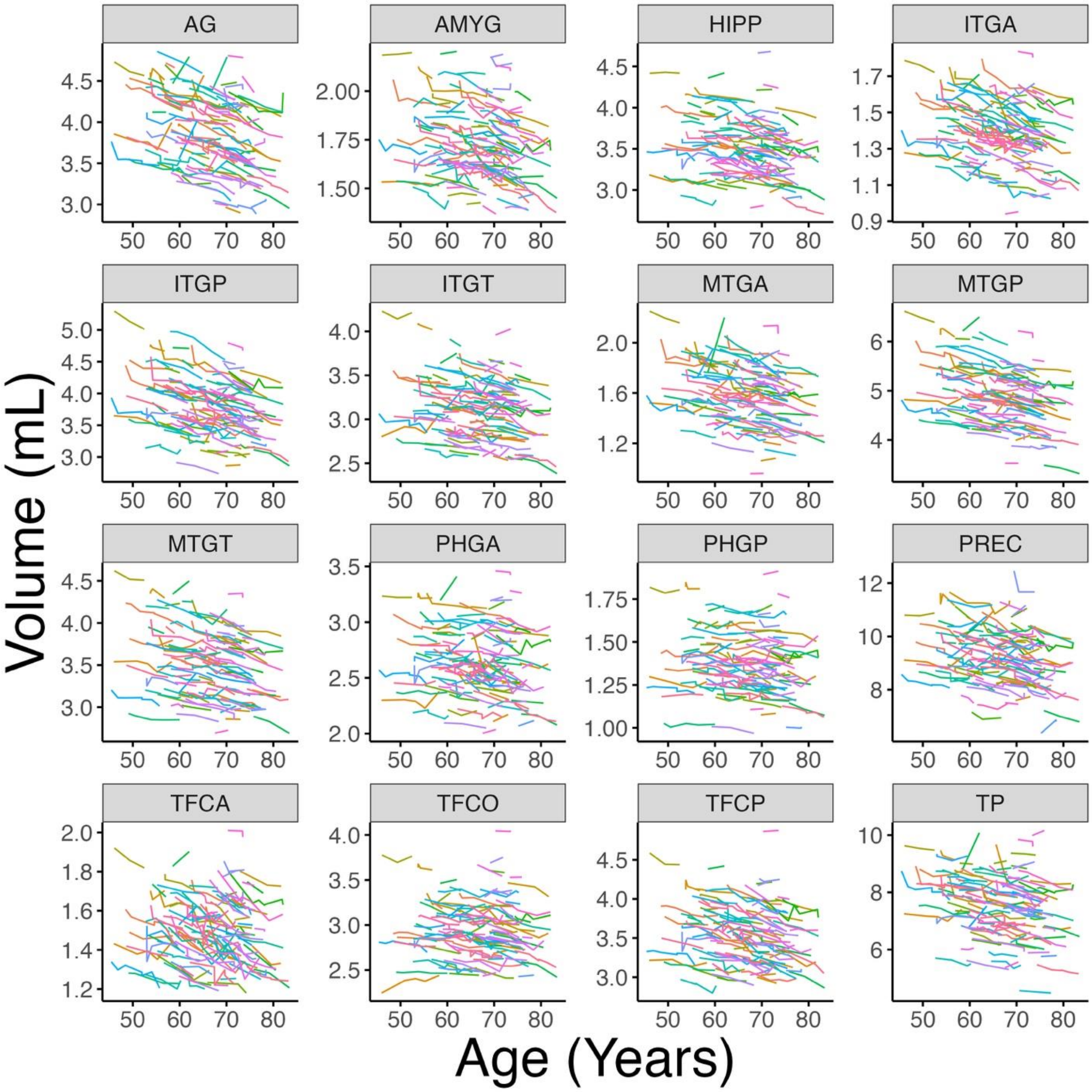


© 2025 Stephenson HG et al. *JAMA Network Open*.

**eFigure 22.** Z-scored volumes in the final z-scoring sample in the OASIS-3 dataset

Separate lines and colors denote different participants.

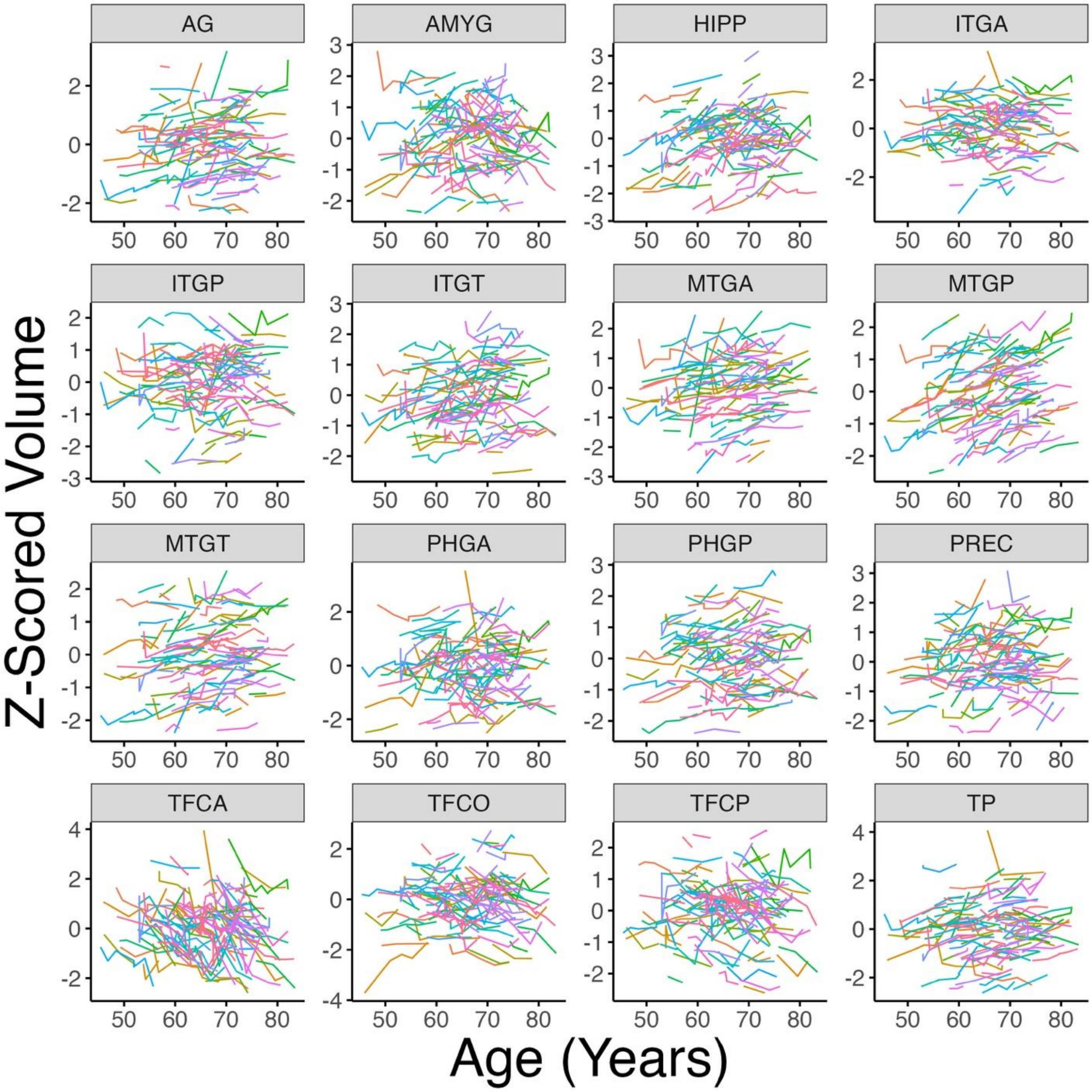


© 2025 Stephenson HG et al. *JAMA Network Open*.

**eFigure 23.** Regional effects of A+ and T+ in the OASIS-3 dataset

**A**: β estimates of interactions between A+ and time that survived FDR correction. **B**: β estimates of interactions between T+ and time that survived FDR correction.

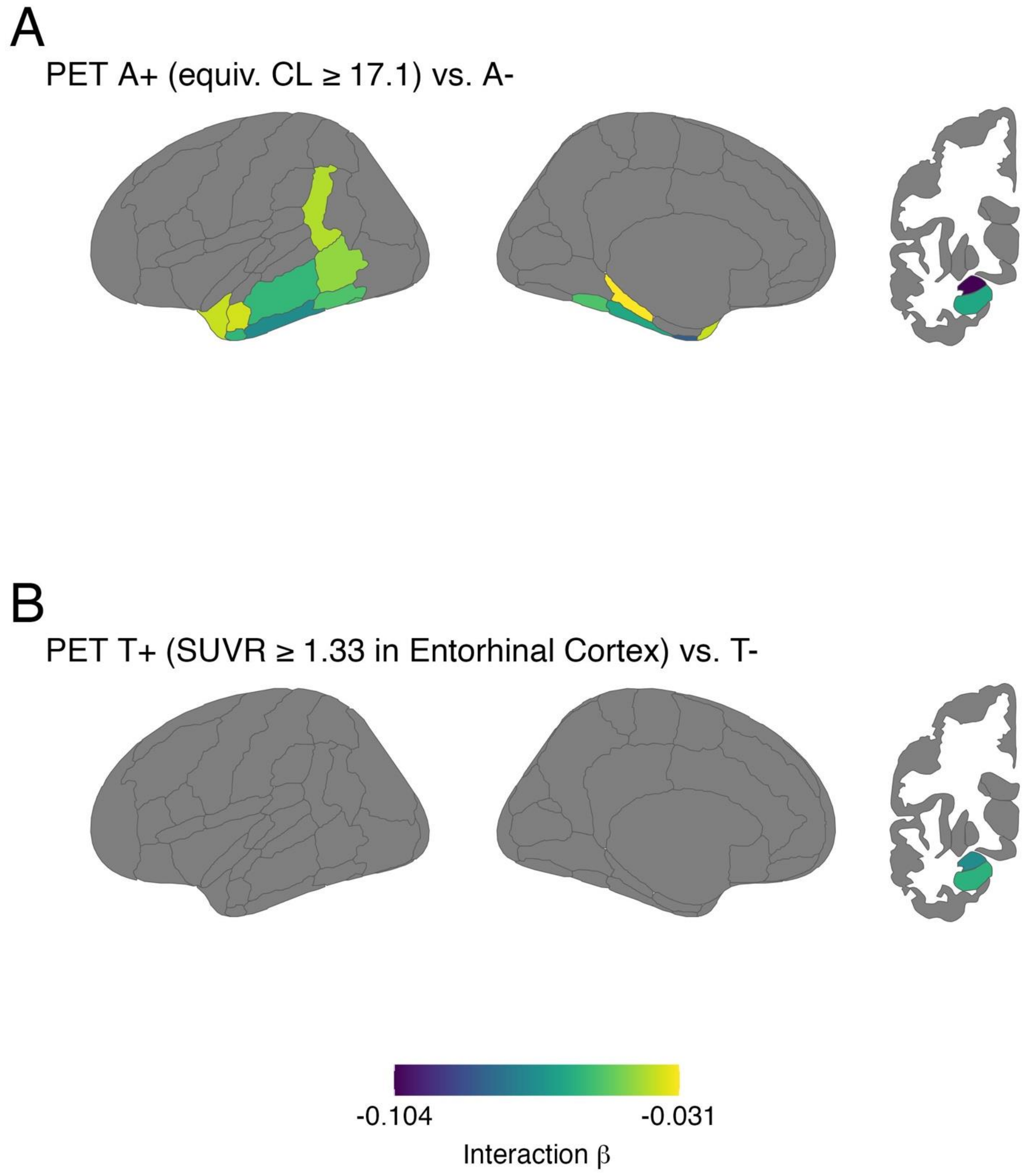


© 2025 Stephenson HG et al. *JAMA Network Open*.

**eFigure 24.** Regional effects of A+ duration and T+ in the OASIS-3 dataset

**A-B**: β estimates of interactions between A+ duration and time that survived FDR correction. **C**: β estimates of interactions between that survived FDR correction for T+.

A

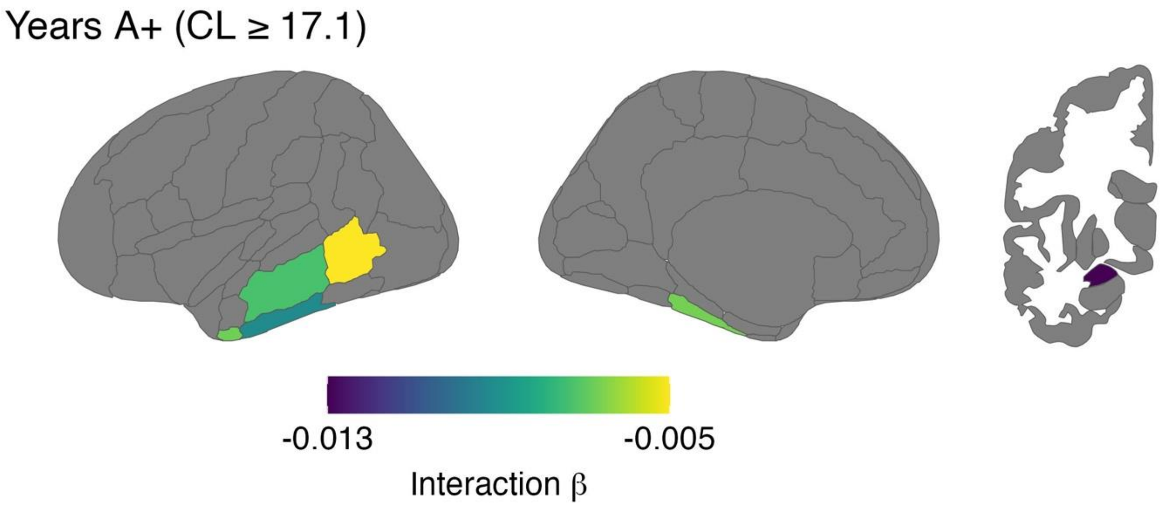


B

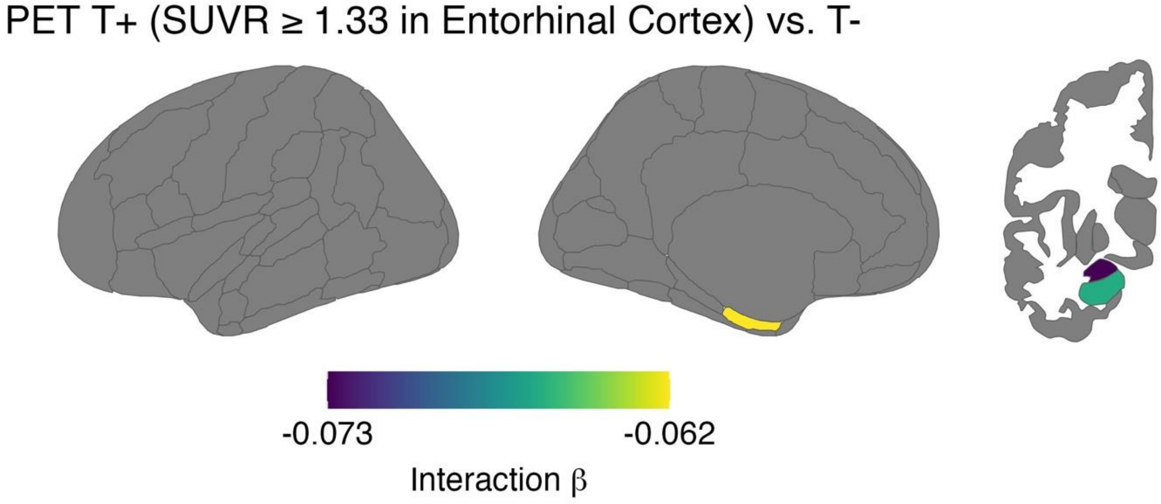


© 2025 Stephenson HG et al. *JAMA Network Open*.

**eTable 22.** Results for each interaction term (A+/- analysis) in the OASIS-3 dataset

Note that uncorrected and FDR-corrected *p*-values may seem identical due to rounding.

| A+ vs. A- (Interaction; OASIS-3) | | | | | | |
|---|---|---|---|---|---|---|
| **ROI** | Slope | $CI_{lower}$ | $CI_{upper}$ | $p_{uncorr}$ | *pFDR* | Partial η2 |
| AG | -0.04 | -0.072 | -0.008 | 0.018 | 0.022 | 0.087 |
| ITGA | -0.057 | -0.087 | -0.027 | $p < 0.001$ | 0.001 | 0.094 |
| ITGP | -0.069 | -0.095 | -0.043 | $p < 0.001$ | $p < 0.001$ | 0.159 |
| ITGT | -0.051 | -0.081 | -0.021 | 0.001 | 0.002 | 0.079 |
| MTGA | -0.036 | -0.067 | -0.005 | 0.024 | 0.027 | 0.062 |
| MTGP | -0.056 | -0.078 | -0.034 | $p < 0.001$ | $p < 0.001$ | 0.184 |
| MTGT | -0.043 | -0.066 | -0.021 | $p < 0.001$ | 0.001 | 0.175 |
| PHGA | -0.041 | -0.085 | 0.003 | 0.067 | 0.072 | 0.021 |
| PHGP | -0.031 | -0.053 | -0.009 | 0.006 | 0.009 | 0.051 |
| PREC | -0.018 | -0.04 | 0.004 | 0.119 | 0.119 | 0.022 |
| TFCA | -0.083 | -0.136 | -0.029 | 0.003 | 0.004 | 0.064 |
| TFCP | -0.061 | -0.09 | -0.031 | $p < 0.001$ | $p < 0.001$ | 0.13 |
| TFCO | -0.051 | -0.088 | -0.015 | 0.007 | 0.009 | 0.062 |
| TP | -0.038 | -0.062 | -0.014 | 0.002 | 0.004 | 0.064 |
| HIPP | -0.06 | -0.096 | -0.024 | 0.001 | 0.002 | 0.07 |
| AMYG | -0.103 | -0.151 | -0.054 | $p < 0.001$ | $p < 0.001$ | 0.097 |
| **T+ vs. T- (Interaction; OASIS-3)** | | | | | | |
| **ROI** | Slope | $CI_{lower}$ | $CI_{upper}$ | $p_{uncorr}$ | *pFDR* | Partial η2 |
| AG | -0.015 | -0.072 | -0.008 | 0.362 | 0.555 | 0.014 |
| ITGA | 0 | -0.087 | -0.027 | 0.981 | 0.981 | 0 |
| ITGP | 0 | -0.095 | -0.043 | 0.971 | 0.981 | 0 |
| ITGT | -0.016 | -0.081 | -0.021 | 0.288 | 0.555 | 0.009 |
| MTGA | -0.016 | -0.067 | -0.005 | 0.306 | 0.555 | 0.014 |
| MTGP | -0.014 | -0.078 | -0.034 | 0.194 | 0.555 | 0.017 |
| MTGT | -0.01 | -0.066 | -0.021 | 0.379 | 0.555 | 0.013 |
| PHGA | -0.055 | -0.085 | 0.003 | 0.013 | 0.07 | 0.04 |
| PHGP | -0.013 | -0.053 | -0.009 | 0.23 | 0.555 | 0.011 |
| PREC | -0.006 | -0.04 | 0.004 | 0.6 | 0.8 | 0.003 |
| TFCA | -0.003 | -0.136 | -0.029 | 0.913 | 0.981 | 0 |
| TFCP | -0.013 | -0.09 | -0.031 | 0.381 | 0.555 | 0.008 |
| TFCO | 0.002 | -0.088 | -0.015 | 0.905 | 0.981 | 0 |
| TP | -0.013 | -0.062 | -0.014 | 0.282 | 0.555 | 0.009 |
| HIPP | -0.057 | -0.096 | -0.024 | 0.002 | 0.032 | 0.065 |
| AMYG | -0.068 | -0.151 | -0.054 | 0.006 | 0.049 | 0.046 |
| **Impaired vs. Unimpaired (Interaction; OASIS-3)** | | | | | | |
| **ROI** | Slope | $CI_{lower}$ | $CI_{upper}$ | $p_{uncorr}$ | *pFDR* | Partial η2 |
| AG | -0.043 | -0.072 | -0.008 | 0.121 | 0.129 | 0.036 |
| ITGA | -0.044 | -0.087 | -0.027 | 0.084 | 0.096 | 0.022 |
| ITGP | -0.051 | -0.095 | -0.043 | 0.023 | 0.036 | 0.038 |
| ITGT | -0.067 | -0.081 | -0.021 | 0.009 | 0.018 | 0.049 |

| | | | | | | |
|---|---|---|---|---|---|---|
| MTGA | -0.035 | -0.067 | -0.005 | 0.183 | 0.183 | 0.021 |
| MTGP | -0.04 | -0.078 | -0.034 | 0.032 | 0.046 | 0.041 |
| MTGT | -0.047 | -0.066 | -0.021 | 0.014 | 0.026 | 0.083 |
| PHGA | -0.121 | -0.085 | 0.003 | 0.001 | 0.004 | 0.062 |
| PHGP | -0.094 | -0.053 | -0.009 | $p < 0.001$ | $p < 0.001$ | 0.151 |
| PREC | -0.038 | -0.04 | 0.004 | 0.047 | 0.058 | 0.038 |
| TFCA | -0.137 | -0.136 | -0.029 | 0.003 | 0.006 | 0.065 |
| TFCP | -0.085 | -0.09 | -0.031 | 0.001 | 0.004 | 0.101 |
| TFCO | -0.1 | -0.088 | -0.015 | 0.002 | 0.004 | 0.08 |
| TP | -0.041 | -0.062 | -0.014 | 0.046 | 0.058 | 0.027 |
| HIPP | -0.161 | -0.096 | -0.024 | $p < 0.001$ | $p < 0.001$ | 0.155 |
| AMYG | -0.182 | -0.151 | -0.054 | $p < 0.001$ | $p < 0.001$ | 0.105 |

© 2025 Stephenson HG et al. *JAMA Network Open*.

**eTable 23.** Results for each main effect (A+/- analysis) in the OASIS-3 dataset

Note that uncorrected and FDR-corrected *p*-values may seem identical due to rounding.

| **A+ vs. A- (Main Effect; OASIS-3)** | | | | | | |
|---|---|---|---|---|---|---|
| **ROI** | Slope | $CI_{lower}$ | $CI_{upper}$ | $p_{uncorr}$ | *pFDR* | Partial η2 |
| AG | 0.108 | -0.445 | 0.661 | 0.703 | 0.75 | 0.001 |
| ITGA | 1.135 | 0.514 | 1.755 | p < 0.001 | 0.007 | 0.064 |
| ITGP | 0.763 | 0.158 | 1.367 | 0.014 | 0.056 | 0.032 |
| ITGT | 0.274 | -0.347 | 0.895 | 0.388 | 0.588 | 0.004 |
| MTGA | 0.829 | 0.279 | 1.379 | 0.004 | 0.028 | 0.045 |
| MTGP | 0.85 | 0.254 | 1.446 | 0.006 | 0.031 | 0.04 |
| MTGT | 0.322 | -0.263 | 0.906 | 0.282 | 0.501 | 0.006 |
| PHGA | 0.305 | -0.41 | 1.019 | 0.404 | 0.588 | 0.004 |
| PHGP | -0.156 | -0.709 | 0.397 | 0.581 | 0.715 | 0.002 |
| PREC | 0.054 | -0.436 | 0.545 | 0.829 | 0.829 | 0 |
| TFCA | 0.656 | 0.023 | 1.29 | 0.044 | 0.116 | 0.022 |
| TFCP | 0.21 | -0.377 | 0.797 | 0.485 | 0.647 | 0.003 |
| TFCO | -0.129 | -0.741 | 0.483 | 0.679 | 0.75 | 0.001 |
| TP | 0.726 | 0.132 | 1.32 | 0.018 | 0.056 | 0.03 |
| HIPP | 0.451 | -0.204 | 1.107 | 0.179 | 0.358 | 0.01 |
| AMYG | 0.411 | -0.164 | 0.985 | 0.163 | 0.358 | 0.01 |
| **T+ vs. T- (Main Effect; OASIS-3)** | | | | | | |
| **ROI** | Slope | $CI_{lower}$ | $CI_{upper}$ | $p_{uncorr}$ | *pFDR* | Partial η2 |
| AG | 0.069 | -0.457 | 0.595 | 0.796 | 0.948 | 0 |
| ITGA | 0.042 | -0.548 | 0.632 | 0.89 | 0.948 | 0 |
| ITGP | 0.032 | -0.543 | 0.608 | 0.913 | 0.948 | 0 |
| ITGT | -0.046 | -0.637 | 0.544 | 0.878 | 0.948 | 0 |
| MTGA | 0.316 | -0.207 | 0.839 | 0.238 | 0.948 | 0.007 |
| MTGP | 0.132 | -0.435 | 0.698 | 0.649 | 0.948 | 0.001 |
| MTGT | 0.05 | -0.505 | 0.606 | 0.859 | 0.948 | 0 |
| PHGA | -0.379 | -1.058 | 0.3 | 0.275 | 0.948 | 0.006 |
| PHGP | 0.152 | -0.374 | 0.678 | 0.572 | 0.948 | 0.002 |
| PREC | -0.097 | -0.564 | 0.369 | 0.683 | 0.948 | 0.001 |
| TFCA | -0.137 | -0.739 | 0.465 | 0.656 | 0.948 | 0.001 |
| TFCP | -0.149 | -0.707 | 0.409 | 0.602 | 0.948 | 0.001 |
| TFCO | 0.084 | -0.497 | 0.665 | 0.778 | 0.948 | 0 |
| TP | -0.04 | -0.605 | 0.524 | 0.889 | 0.948 | 0 |
| HIPP | -0.46 | -1.083 | 0.163 | 0.149 | 0.948 | 0.011 |
| AMYG | -0.018 | -0.565 | 0.528 | 0.948 | 0.948 | 0 |
| **Impaired vs. Unimpaired (Main Effect; OASIS-3)** | | | | | | |
| **ROI** | Slope | $CI_{lower}$ | $CI_{upper}$ | $p_{uncorr}$ | *pFDR* | Partial η2 |
| AG | 0.321 | -0.537 | 1.178 | 0.464 | 0.619 | 0.003 |
| ITGA | -0.584 | -1.545 | 0.378 | 0.236 | 0.472 | 0.008 |
| ITGP | -0.361 | -1.299 | 0.577 | 0.452 | 0.619 | 0.003 |
| ITGT | -0.405 | -1.368 | 0.557 | 0.41 | 0.619 | 0.004 |

© 2025 Stephenson HG et al. *JAMA Network Open*.

| | | | | | | |
|---|---|---|---|---|---|---|
| MTGA | -0.529 | -1.381 | 0.324 | 0.226 | 0.472 | 0.008 |
| MTGP | -0.195 | -1.118 | 0.728 | 0.679 | 0.836 | 0.001 |
| MTGT | -0.074 | -0.98 | 0.832 | 0.873 | 0.911 | 0 |
| PHGA | -1.092 | -2.198 | 0.015 | 0.055 | 0.218 | 0.02 |
| PHGP | -0.467 | -1.324 | 0.391 | 0.287 | 0.511 | 0.006 |
| PREC | -0.049 | -0.809 | 0.711 | 0.899 | 0.911 | 0 |
| TFCA | -0.659 | -1.642 | 0.324 | 0.19 | 0.472 | 0.009 |
| TFCP | -0.974 | -1.884 | -0.064 | 0.037 | 0.198 | 0.023 |
| TFCO | -0.723 | -1.671 | 0.225 | 0.137 | 0.437 | 0.012 |
| TP | -0.053 | -0.973 | 0.867 | 0.911 | 0.911 | 0 |
| HIPP | -1.27 | -2.285 | -0.254 | 0.015 | 0.121 | 0.031 |
| AMYG | -1.169 | -2.06 | -0.278 | 0.011 | 0.121 | 0.034 |

© 2025 Stephenson HG et al. *JAMA Network Open*.

**eTable 24.** Results for each interaction term (A+ duration analysis) in the OASIS-3 dataset

Note that uncorrected and FDR-corrected *p*-values may seem identical due to rounding.

| A+ Duration (Interaction; OASIS-3) | | | | | | |
|---|---|---|---|---|---|---|
| **ROI** | Slope | $CI_{lower}$ | $CI_{upper}$ | $p_{uncorr}$ | *pFDR* | Partial η2 |
| AG | -0.005 | -0.011 | 0 | 0.051 | 0.101 | 0.045 |
| ITGA | -0.007 | -0.012 | -0.002 | 0.006 | 0.022 | 0.049 |
| ITGP | -0.009 | -0.014 | -0.005 | $p < 0.001$ | 0.001 | 0.1 |
| ITGT | -0.005 | -0.01 | 0 | 0.045 | 0.101 | 0.026 |
| MTGA | -0.004 | -0.009 | 0.001 | 0.131 | 0.161 | 0.023 |
| MTGP | -0.008 | -0.011 | -0.004 | $p < 0.001$ | 0.001 | 0.104 |
| MTGT | -0.005 | -0.009 | -0.001 | 0.008 | 0.023 | 0.072 |
| PHGA | -0.004 | -0.011 | 0.003 | 0.275 | 0.294 | 0.007 |
| PHGP | -0.003 | -0.006 | 0.001 | 0.166 | 0.189 | 0.011 |
| PREC | 0 | -0.004 | 0.004 | 0.992 | 0.992 | 0 |
| TFCA | -0.008 | -0.017 | 0 | 0.065 | 0.104 | 0.021 |
| TFCP | -0.007 | -0.012 | -0.002 | 0.008 | 0.023 | 0.052 |
| TFCO | -0.005 | -0.011 | 0.001 | 0.095 | 0.127 | 0.02 |
| TP | -0.004 | -0.008 | 0 | 0.072 | 0.104 | 0.02 |
| HIPP | -0.006 | -0.012 | 0 | 0.062 | 0.104 | 0.02 |
| AMYG | -0.013 | -0.021 | -0.005 | 0.001 | 0.006 | 0.055 |
| **T+ vs. T- (Interaction; OASIS-3)** | | | | | | |
| **ROI** | Slope | $CI_{lower}$ | $CI_{upper}$ | $p_{uncorr}$ | *pFDR* | Partial η2 |
| AG | -0.017 | -0.011 | 0 | 0.314 | 0.418 | 0.016 |
| ITGA | -0.004 | -0.012 | -0.002 | 0.78 | 0.791 | 0.001 |
| ITGP | -0.005 | -0.014 | -0.005 | 0.741 | 0.791 | 0.001 |
| ITGT | -0.023 | -0.01 | 0 | 0.139 | 0.319 | 0.018 |
| MTGA | -0.02 | -0.009 | 0.001 | 0.209 | 0.347 | 0.019 |
| MTGP | -0.017 | -0.011 | -0.004 | 0.14 | 0.319 | 0.022 |
| MTGT | -0.015 | -0.009 | -0.001 | 0.223 | 0.347 | 0.021 |
| PHGA | -0.061 | -0.011 | 0.003 | 0.007 | 0.039 | 0.047 |
| PHGP | -0.019 | -0.006 | 0.001 | 0.089 | 0.319 | 0.023 |
| PREC | -0.013 | -0.004 | 0.004 | 0.239 | 0.347 | 0.016 |
| TFCA | -0.015 | -0.017 | 0 | 0.582 | 0.716 | 0.003 |
| TFCP | -0.02 | -0.012 | -0.002 | 0.188 | 0.347 | 0.018 |
| TFCO | -0.005 | -0.011 | 0.001 | 0.791 | 0.791 | 0.001 |
| TP | -0.019 | -0.008 | 0 | 0.134 | 0.319 | 0.018 |
| HIPP | -0.066 | -0.012 | 0 | 0.001 | 0.01 | 0.077 |
| AMYG | -0.073 | -0.021 | -0.005 | 0.004 | 0.036 | 0.049 |
| **Impaired vs. Unimpaired (Interaction; OASIS-3)** | | | | | | |
| **ROI** | Slope | $CI_{lower}$ | $CI_{upper}$ | $p_{uncorr}$ | *pFDR* | Partial η2 |
| AG | -0.044 | -0.011 | 0 | 0.114 | 0.122 | 0.033 |
| ITGA | -0.047 | -0.012 | -0.002 | 0.07 | 0.08 | 0.024 |
| ITGP | -0.053 | -0.014 | -0.005 | 0.021 | 0.034 | 0.039 |

© 2025 Stephenson HG et al. *JAMA Network Open*.

| | | | | | | |
|---|---|---|---|---|---|---|
| ITGT | -0.073 | -0.01 | 0 | 0.006 | 0.012 | 0.052 |
| MTGA | -0.039 | -0.009 | 0.001 | 0.145 | 0.145 | 0.023 |
| MTGP | -0.042 | -0.011 | -0.004 | 0.032 | 0.041 | 0.038 |
| MTGT | -0.051 | -0.009 | -0.001 | 0.013 | 0.023 | 0.073 |
| PHGA | -0.126 | -0.011 | 0.003 | 0.001 | 0.003 | 0.064 |
| PHGP | -0.098 | -0.006 | 0.001 | p < 0.001 | p < 0.001 | 0.156 |
| PREC | -0.041 | -0.004 | 0.004 | 0.033 | 0.041 | 0.043 |
| TFCA | -0.143 | -0.017 | 0 | 0.002 | 0.005 | 0.066 |
| TFCP | -0.09 | -0.012 | -0.002 | 0.001 | 0.003 | 0.098 |
| TFCO | -0.105 | -0.011 | 0.001 | 0.001 | 0.003 | 0.082 |
| TP | -0.045 | -0.008 | 0 | 0.033 | 0.041 | 0.031 |
| HIPP | -0.169 | -0.012 | 0 | p < 0.001 | p < 0.001 | 0.154 |
| AMYG | -0.187 | -0.021 | -0.005 | p < 0.001 | p < 0.001 | 0.103 |

© 2025 Stephenson HG et al. *JAMA Network Open*.

**eTable 25.** Results for each main effect (A+ duration analysis) in the OASIS-3 dataset

Note that uncorrected and FDR-corrected *p*-values may seem identical due to rounding.

| **A+ Duration (Main Effect; OASIS-3)** | | | | | | |
|---|---|---|---|---|---|---|
| **ROI** | Slope | $CI_{lower}$ | $CI_{upper}$ | $p_{uncorr}$ | *pFDR* | Partial η2 |
| AG | 0.012 | -0.07 | 0.095 | 0.774 | 0.884 | 0 |
| ITGA | 0.103 | 0.008 | 0.197 | 0.034 | 0.552 | 0.024 |
| ITGP | 0.054 | -0.038 | 0.145 | 0.25 | 0.629 | 0.007 |
| ITGT | 0.034 | -0.058 | 0.127 | 0.468 | 0.738 | 0.003 |
| MTGA | 0.064 | -0.02 | 0.147 | 0.136 | 0.622 | 0.012 |
| MTGP | 0.069 | -0.021 | 0.159 | 0.136 | 0.622 | 0.012 |
| MTGT | 0.04 | -0.047 | 0.127 | 0.372 | 0.662 | 0.004 |
| PHGA | -0.004 | -0.111 | 0.102 | 0.937 | 0.937 | 0 |
| PHGP | -0.028 | -0.11 | 0.055 | 0.507 | 0.738 | 0.002 |
| PREC | 0.015 | -0.058 | 0.088 | 0.684 | 0.884 | 0.001 |
| TFCA | 0.069 | -0.026 | 0.164 | 0.156 | 0.622 | 0.011 |
| TFCP | 0.053 | -0.034 | 0.141 | 0.235 | 0.629 | 0.008 |
| TFCO | 0.014 | -0.077 | 0.105 | 0.766 | 0.884 | 0 |
| TP | 0.049 | -0.041 | 0.138 | 0.289 | 0.629 | 0.006 |
| HIPP | 0.05 | -0.048 | 0.148 | 0.314 | 0.629 | 0.005 |
| AMYG | 0.006 | -0.08 | 0.092 | 0.887 | 0.937 | 0 |
| **T+ vs. T- (Main Effect; OASIS-3)** | | | | | | |
| **ROI** | Slope | $CI_{lower}$ | $CI_{upper}$ | $p_{uncorr}$ | *pFDR* | Partial η2 |
| AG | 0.08 | -0.451 | 0.611 | 0.767 | 0.942 | 0 |
| ITGA | 0.196 | -0.412 | 0.804 | 0.528 | 0.942 | 0.002 |
| ITGP | 0.18 | -0.408 | 0.769 | 0.549 | 0.942 | 0.002 |
| ITGT | -0.03 | -0.626 | 0.566 | 0.921 | 0.982 | 0 |
| MTGA | 0.456 | -0.081 | 0.992 | 0.098 | 0.942 | 0.015 |
| MTGP | 0.268 | -0.312 | 0.849 | 0.366 | 0.942 | 0.004 |
| MTGT | 0.071 | -0.49 | 0.633 | 0.804 | 0.942 | 0 |
| PHGA | -0.258 | -0.944 | 0.428 | 0.463 | 0.942 | 0.003 |
| PHGP | 0.166 | -0.364 | 0.697 | 0.539 | 0.942 | 0.002 |
| PREC | -0.114 | -0.584 | 0.356 | 0.635 | 0.942 | 0.001 |
| TFCA | -0.069 | -0.679 | 0.541 | 0.824 | 0.942 | 0 |
| TFCP | -0.205 | -0.767 | 0.356 | 0.475 | 0.942 | 0.003 |
| TFCO | 0.004 | -0.583 | 0.591 | 0.99 | 0.99 | 0 |
| TP | 0.102 | -0.474 | 0.678 | 0.729 | 0.942 | 0.001 |
| HIPP | -0.425 | -1.055 | 0.205 | 0.188 | 0.942 | 0.009 |
| AMYG | 0.114 | -0.44 | 0.668 | 0.687 | 0.942 | 0.001 |
| **Impaired vs. Unimpaired (Main Effect; OASIS-3)** | | | | | | |
| **ROI** | Slope | $CI_{lower}$ | $CI_{upper}$ | $p_{uncorr}$ | *pFDR* | Partial η2 |
| AG | 0.333 | -0.519 | 1.185 | 0.445 | 0.647 | 0.003 |
| ITGA | -0.387 | -1.362 | 0.588 | 0.438 | 0.647 | 0.003 |
| ITGP | -0.196 | -1.14 | 0.747 | 0.684 | 0.912 | 0.001 |
| ITGT | -0.38 | -1.336 | 0.577 | 0.437 | 0.647 | 0.003 |

© 2025 Stephenson HG et al. *JAMA Network Open*.

| | | | | | | |
|---|---|---|---|---|---|---|
| MTGA | -0.36 | -1.221 | 0.501 | 0.414 | 0.647 | 0.004 |
| MTGP | -0.031 | -0.962 | 0.899 | 0.948 | 0.948 | 0 |
| MTGT | -0.041 | -0.942 | 0.86 | 0.929 | 0.948 | 0 |
| PHGA | -0.973 | -2.074 | 0.127 | 0.085 | 0.306 | 0.016 |
| PHGP | -0.469 | -1.319 | 0.382 | 0.282 | 0.644 | 0.006 |
| PREC | -0.067 | -0.821 | 0.688 | 0.862 | 0.948 | 0 |
| TFCA | -0.573 | -1.553 | 0.408 | 0.254 | 0.644 | 0.007 |
| TFCP | -1.014 | -1.915 | -0.113 | 0.029 | 0.153 | 0.025 |
| TFCO | -0.805 | -1.746 | 0.137 | 0.096 | 0.306 | 0.015 |
| TP | 0.109 | -0.815 | 1.033 | 0.817 | 0.948 | 0 |
| HIPP | -1.215 | -2.225 | -0.205 | 0.019 | 0.153 | 0.029 |
| AMYG | -1.036 | -1.925 | -0.147 | 0.023 | 0.153 | 0.027 |

© 2025 Stephenson HG et al. *JAMA Network Open*.

**eReferences.**


1. Ashburner J, Ridgway GR. Symmetric diffeomorphic modeling of longitudinal structural MRI. Front Neurosci. 2012;6:197. doi:10.3389/fnins.2012.00197

2. Ashburner J, Friston KJ. Unified segmentation. Neuroimage. 2005;26(3):839-851. doi:10.1016/j.neuroimage.2005.02.018

3. Ashburner J. A fast diffeomorphic image registration algorithm. Neuroimage. 2007;38(1):95-113. doi:10.1016/j.neuroimage.2007.07.007

4. Fjell AM, Westlye LT, Grydeland H, et al. Critical ages in the life course of the adult brain: nonlinear subcortical aging. Neurobiol Aging. 2013;34(10):2239-2247. doi:10.1016/j.neurobiolaging.2013.04.006

5. Crivello F, Tzourio-Mazoyer N, Tzourio C, Mazoyer B. Longitudinal assessment of global and regional rate of grey matter atrophy in 1,172 healthy older adults: modulation by sex and age. PLoS One. 2014;9(12):e114478. doi:10.1371/journal.pone.0114478

6. Benaglia T, Chauveau D, Hunter DR, Young D. mixtools: An R Package for Analyzing Finite Mixture Models. Journal of Statistical Software. 2009;32(6):1-29.

7. LaMontagne PJ, Benzinger TLS, Morris JC, et al. OASIS-3: Longitudinal Neuroimaging, Clinical, and Cognitive Dataset for Normal Aging and Alzheimer Disease. medRxiv. Published online January 1, 2019:2019.12.13.19014902. doi:10.1101/2019.12.13.19014902


© 2025 Stephenson HG et al. *JAMA Network Open*.
