# Supplementary material for: Abnormal Amyloid-β Duration, Tau, and Neurodegeneration in Cranial Images
Source: JAMA Netw Open. 2025 Sep 9;8(9):e2531093. doi: 10.1001/jamanetworkopen.2025.31093 (PMC12421349; doi:10.1001/jamanetworkopen.2025.31093)
Supplement: Supplement 2. — Data Sharing Statement [file jamanetwopen-e2531093-s002.pdf]

Data Sharing Statement

Stephenson. Abnormal Amyloid-β Duration, Tau, and Neurodegeneration in Cranial Images. *JAMA Netw Open*. Published September 09, 2025. doi:10.1001/jamanetworkopen.2025.31093

Data
Data available: Yes
Data types: Participant data with identifiers
How to access data: All data are available upon reason request from the Wisconsin ADRC.
When available: With publication

Supporting Documents
Document types: Statistical/analytic code
How to access documents: These may be requested from the primary author at hstephenson2@wisc.edu.
When available: With publication

Additional Information
Who can access the data: Researchers whose proposed use of the data has been approved.
Types of analyses: Any purpose.
Mechanisms of data availability: After approval of a proposal.
